# Synthesis of Bis-Thioacid Derivatives of Diarylethene and Their Photochromic Properties

**DOI:** 10.1021/acsomega.4c05945

**Published:** 2024-11-18

**Authors:** Pramod Aryal, Jonathan Bietsch, Gowri Sankar Grandhi, Richard Chen, Surya B. Adhikari, Ephraiem S. Sarabamoun, Joshua J. Choi, Guijun Wang

**Affiliations:** † Department of Chemistry and Biochemistry, Old Dominion University, Norfolk, Virginia 23529, United States; ‡ Department of Chemical Engineering, University of Virginia, Charlottesville, Virginia 22904, United States

## Abstract

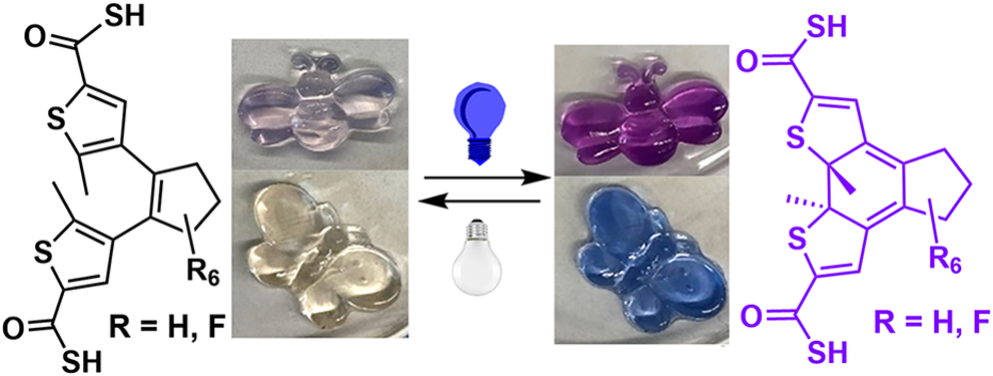

Diarylethenes (DAEs) are an important class of photoswitchable compounds that typically undergo reversible photochemical conversions between the open and closed cyclized forms upon treatment with UV light or visible light. In this study, we introduced thioacid functional groups to several photochromic dithienylethene (DTE) derivatives and established a method that can be used to prepare these photoswitchable thioacids. Four thioacid-functionalized diarylethene derivatives were synthesized through the activation of carboxylic acids with *N*-hydroxysuccinimide, followed by reactions with sodium hydrosulfide with yields over 90%. These derivatives exhibited reversible photoswitching and photochromic properties upon treatment with ultraviolet (UV) and visible lights. The thioacid groups on these compounds can act as reaction sites for attaching other desirable functionalities. The photochromic properties of these new derivatives were characterized by using ultraviolet–visible (UV–vis) spectroscopy. The photocyclizations of one of the derivatives and its potassium salt were also characterized by using nuclear magnetic resonance (NMR) spectroscopy. The anions of the thioacid formed water-soluble photochromic systems, and their applications as colorimetric sensors in agarose hydrogels were demonstrated.

## Introduction

Photoswitchable molecules are important organic compounds with fascinating light-tunable properties and practical applications.^1^ Among the different types of molecular switches, those based on diarylethene (DAE) or dithienylethene (DTE) are especially interesting systems due to the light-controlled reversible photoisomerization between the open and closed forms.^2^ Upon treatment with ultraviolet (UV) light, DAE derivatives undergo photocyclization from a colorless open form to a cyclized closed form, which typically is colored and has distinct UV absorptions. The cyclized forms are thermally stable, and the wavelength of the absorption can be tuned by changing the structures of the DAE derivatives.^3,4^ DAE-based molecular switches have been explored for many applications as molecular devices and in optical electronics.^5,6^ For instance, carboxylic acid functionalized derivatives were utilized to connect quantum dots to obtain hybrid inorganic materials.^7−9^ They have also been developed to form fluorescent sensors for metal ions and for bioimaging and photodynamic therapy.^10,11^ The carboxylic acid functionalized DAEs have been used as a linker for the formation of many different derivatives including ester and amides.^12−15^ Several examples of using DAE carboxylic acid derivatives for the formation of photochromic amides have been reported, including the formation of a hybrid antibacterial agent with fluoroquinolone,^16^ and as building blocks for the formation of cyclic peptides.^17^ DAE dicarboxylic acid was also used for the fabrication of a biosensor, which exhibited colorimetric sensing with silk fibroin and glucose.^18^

In addition to applications in photoelectronic devices, DAE derivatives have been used for the preparation of soft materials, such as supramolecular gels. Feringa et al. have synthesized and studied diarylethene-containing amide derivatives and urea derivatives as photoresponsive organogelators.^19,20^ Several dithienylcyclopentene amides that were prepared from the corresponding diacids were also shown to be organogelators, forming gels in benzene, toluene, and xylene at low concentrations.^21^ A tetrapeptide dithienylcyclopentene derivative formed gels in acetone, acetonitrile, and tetrahydrofuran (THF). The organogels in THF exhibited reversible photochromic properties.^22^ Tian et al. also reported reversible photocontrolled gels using dithienylethene-doped lecithin micelles.^23^ Perfluorinated diarylethene dialdehyde was used as photochromic acceptor in the preparation of photoswitchable organogels.^24^ Recently, both the hydrogenated cyclopentene derivative and hexafluorinated cyclopentene derivatives have been incorporated with a sugar derivative through an ester linkage. These derivatives performed as organogelators in several organic solvents, as well as in aqueous solutions.^25^ These are interesting materials with thermally controlled phase transition properties as well as photochromic or photocontrolled phase transitions.

Thioacids are a useful class of compounds containing the −COSH functional group.^26^ They have been used for the formation of thiocarboxylate derivatives including thioesters,^27^ thionoesters, and dithioesters.^28^ A thiocarboxylate salt was also used in the enantioselective nucleophilic substitution reaction to produce chiral thioesters.^29^ These compounds can be utilized as synthetic intermediates for amide and peptidomimetic synthesis.^30,31^ Recently, thioacid derivatives of amino acids were employed as the intermediates for the synthesis of glycoproteins.^32^ The structures of thiophene-2-thiocarboxylate have been elucidated,^33^ and they are used as ligands for metal ions.^34^ Using Lawesson’s reagent, 10,12-pentacosadiynoic acid (PCDA) was converted to the corresponding thioacid, and the resulting thioacid-functionalized diacetylene lipid was used as a sensor for ethylene.^35^

Several methods can be used to prepare thioacids, typically starting from the corresponding carboxylic acids as the starting materials. Thioacids have been prepared by reaction of the corresponding acid chloride with sodium thiolate.^29^ Thioacids have also been prepared from carboxylic acids using Lawesson’s reagents under MW conditions.^36^ A series of thioacids were synthesized in water using acyl benzotriazoles and sodium hydrosulfide (NaSH) at room temperature in good yields.^37^ A one-step catalytic thiolation to prepare peptide thioacid was reported using potassium thioacetate (KSAc) as the source of sulfur and diacetylsulfide (Ac_2_S) in catalytic amounts through a mixed acetic anhydride intermediate.^38^ Amino acid derivatives have been converted to the corresponding thioacids using thioacetic acid, which were then utilized for peptide coupling reactions.^39^ Peptide thioacids have been prepared by activating carboxylic acids with EDC and treating the resulting activated carboxylic acid with sodium sulfide.^40^ 2-Cyanoethyl thioester was used as a protecting group for thioacids during peptide synthesis.^41^ Peptide thiocarboxylate was also synthesized in water from bis(2-sulfanylethyl)amido peptides and triisopropylsilylthiol.^42^ More recently, α-methylphenacyl thioesters were used as the precursor for thioacid, and this has facilitated peptide synthesis.^43^

**Figure 1:**
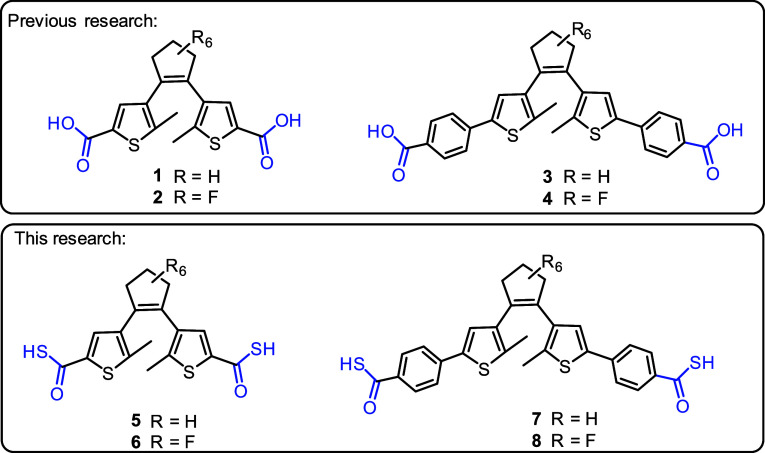
Structures of target thioacid-functionalized diarylethene derivatives.

Among the many classes of diarylethene derivatives, thioacid-functionalized diarylethenes have not garnered much attention yet. In this research, we introduce the thioacid group directly to the thiophene or through a phenyl spacer on the diarylethene. The diarylethene dicarboxylic acids with the perhydrocyclopentene bridge or the perfluorocyclopentene bridge are shown in Figure 1. Compounds **1**–**4** are dicarboxylate-functionalized diarylethene derivatives, which have been reported in the literature and have demonstrated applications for several different systems.^7,8,44−46^ The conversion of the carboxylic acid functions to thioacid functions will lead to the formation of novel diarylethene-based photochromic compounds **5**–**8**. These thiocarboxylic acid derivatives create opportunities for applications as new reagents and as functional materials. These are novel compounds that may exhibit interesting properties and can be used for other applications as new materials.

## Results and Discussions

**Scheme 1:**
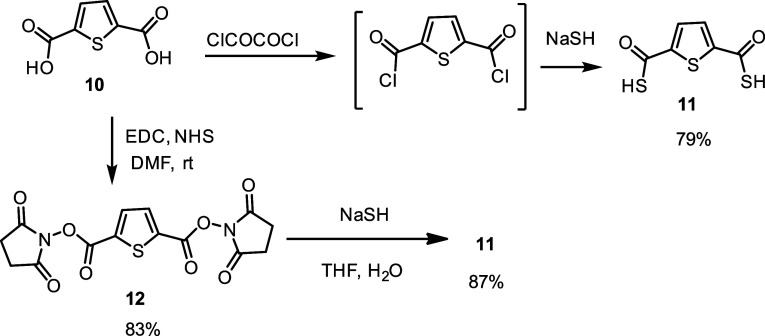
Conditions of the Conversion of Thiophene Diacids to the Dithioacids

Several methods are available for the synthesis of thioacid from the corresponding carboxylic acids, as mentioned above. The dicarboxylic acid derivatives **1** and **2** were synthesized from 2-chloro-5-methyl thiophene in three steps according to the literature.^7,8^ Considering the high cost of these diarylethene diacids, to find a suitable protocol for the synthesis of the dithienylethene dithioacids, we used thiophene-2,5-dicarboxylic acid **10** as the test compound for reaction conditions. As shown in Scheme 1, two methods were used to convert carboxylic acid to thioacid. Oxalyl chloride was utilized to convert the compound to the corresponding acid chloride *in situ* and subsequent reaction with sodium hydrosulfide afforded bis-thioacid **11** in 79% yield. Alternatively, using a two-step procedure, we converted the acid to the corresponding *N*-hydroxysuccinimide (NHS) derivative **12**, which was isolated and purified before it was reacted with sodium hydrosulfide to form the thioacid **11** in 72% overall yield. These reactions demonstrated that the methods are feasible for the preparation of dithioacids.

**Scheme 2:**
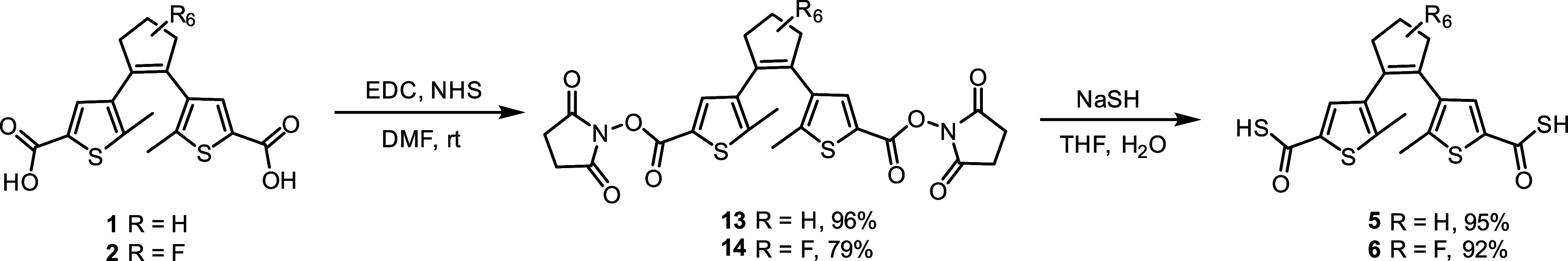
Synthesis of Perfluorinated Diarylethene Thioacids **5** and **6**

With the success of utilizing template compound **10**, we then turned to converting compound **1** to the thioacid derivative **5**. The acid chloride method is a straightforward method to prepare the thioacid in a one-pot manner. This method did not produce the desired thioacid compound **5**. An intractable mixture of undesired products was obtained, and the thioacid was not isolated. We also tried another one-pot reaction method using Lawesson’s reagent under microwave conditions. Despite the method being shown to be useful for the conversion of carboxylic acids to thioacids, for the diarylethene diacid, only a very small amount of product was obtained, and the residual Lawesson’s reagent and many by-products of the reactions made isolation difficult. The diarylethene diacid was converted to complex oligomeric derivatives, and the desired thioacid derivatives could not be obtained. We also treated compound **1** with acetic anhydride and the resulting mixed anhydride intermediate was reacted with NaSH. The procedure worked well for amino acid derivatives as reported in the literature;^39^ however, this method also failed to produce the desired dithioacid **5**. Since several methods failed to generate the thioacids from the carboxylic acids directly, we focused on the two-step method and converted the diacids to *N*-hydroxy succinimide derivatives first, as shown in Scheme 2, which activates the carboxylic acid compound before treating this intermediate with NaSH. A major benefit to using this method is that the stability of the NHS intermediate allows for isolation and purification. We hypothesized better results for synthesizing the corresponding dithioacid derivative. After several unsuccessful attempts, we were able to find a suitable condition for the transformation. The NHS intermediate **13** was converted to the thioacid **5** successfully in 95% yield. After the method was established for the preparation of the bis-thioacid, a similar method was used to prepare other diarylethene derivatives such as the fluorinated derivative **6**.

**Scheme 3:**
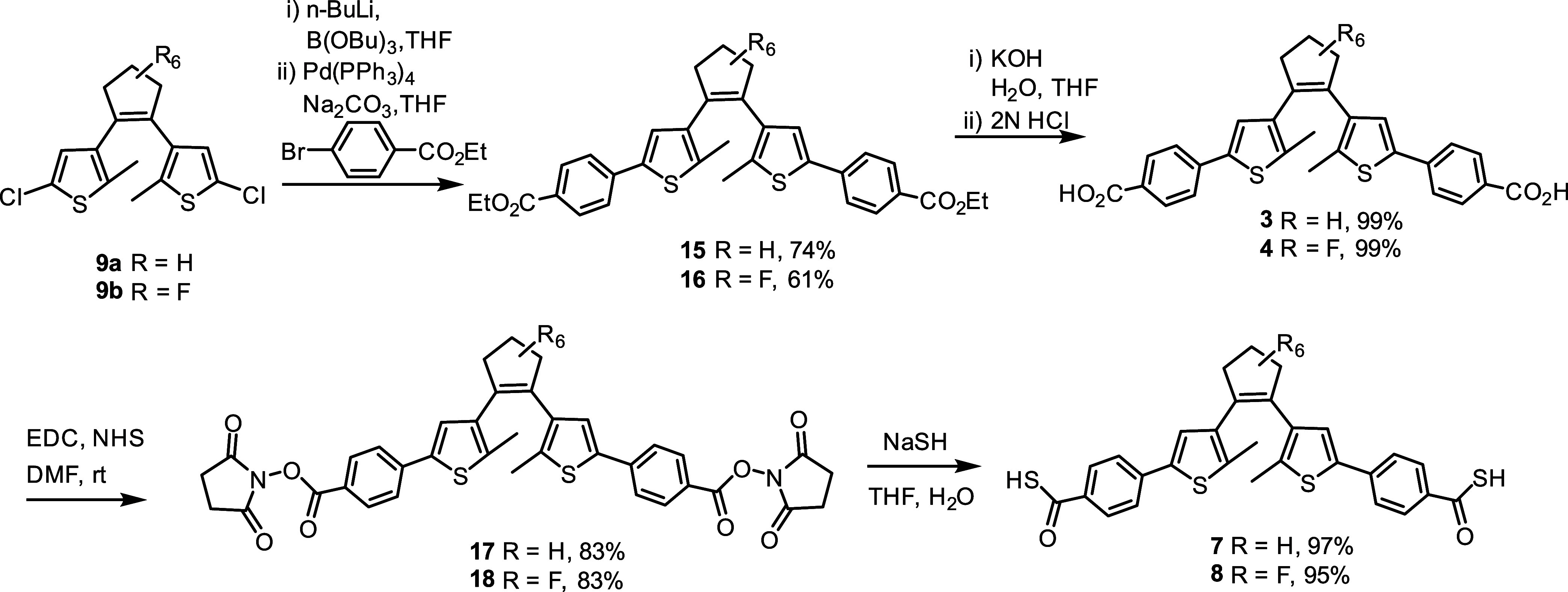
Synthesis of Dithienylethene Dibenzothioacids **7** and **8**

We then extended the method to synthesize diarylethene derivatives **7** and **8**. As shown in Scheme 3, the reaction started from the dichlorothiophene derivative **9a** or the fluorinated compound **9b**. The Suzuki coupling reaction afforded intermediate esters **15** or **16**, respectively, and further hydrolysis afforded the corresponding carboxylic acids **3** or **4**. Then, following the above methods, the acids were converted to the corresponding *N*-hydroxysuccinimide derivatives **17** or **18**, and treatment with NaSH afforded the desired thioacids **7** or **8**.

**Figure 2:**
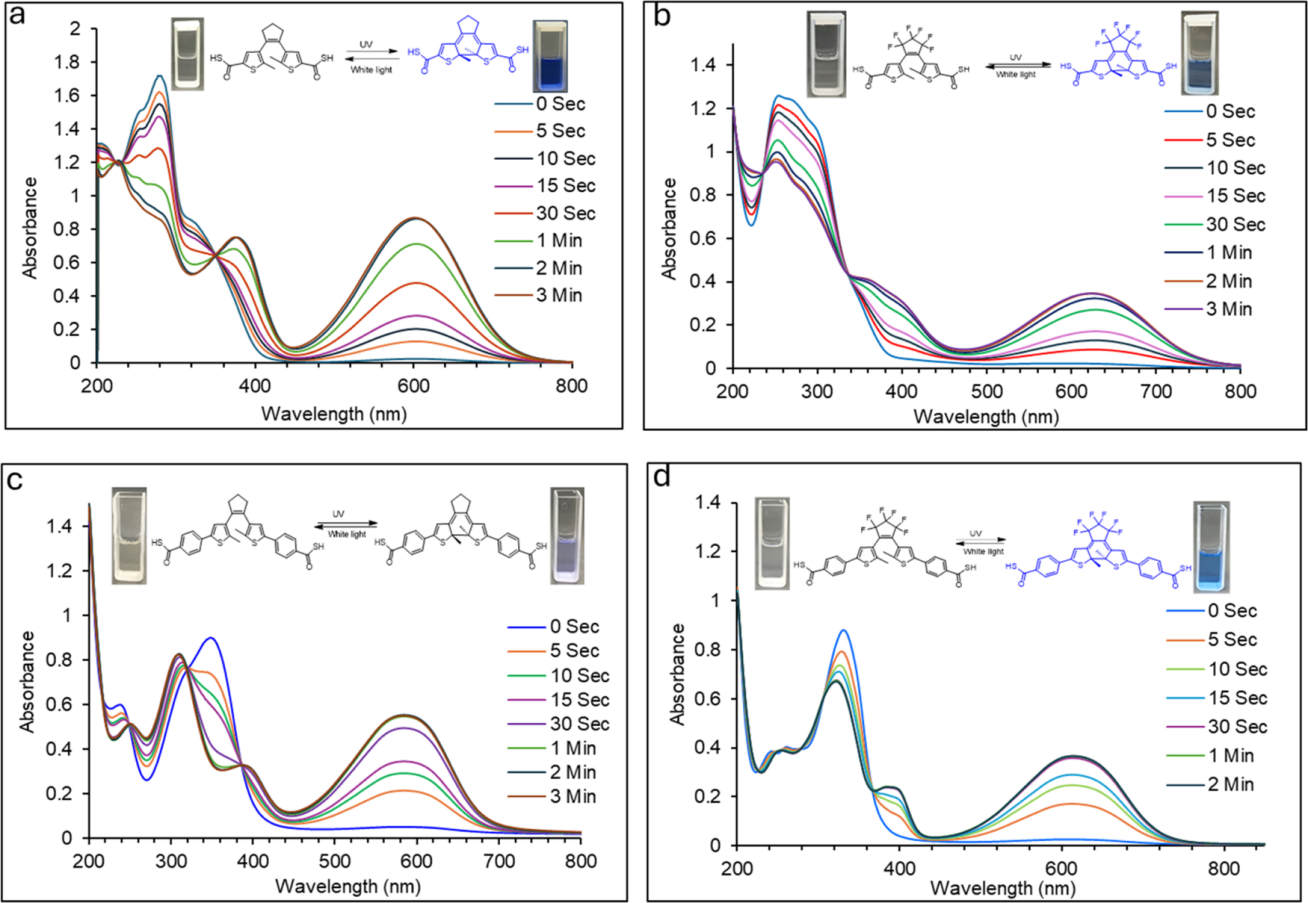
UV–vis spectra of compounds **5**–**8** upon UV irradiation (302 nm, 6W TLC lamp) at different time points: (a) compound **5** in acetonitrile (0.065 mM); (b) compound **6** in acetonitrile (0.10 mM); (c) compound **7** in acetonitrile (0.05 mM); and (d) compound **8** (0.025 mM) in acetonitrile.

After these thioacids were synthesized, we analyzed their photochromic properties, which are included in Figure 2 and ESI Figures S21–S25. All four compounds exhibited photochromic properties upon irradiation with UV light. Compound **5** exhibited solvent-dependent photochromic properties. In both acetonitrile and methanol, UV exposure resulted in cyclization to the closed form, which exhibited purple-violet solutions (Figures 2a and S21). However, the maximum absorptions λ_max_ in the two solvents exhibited significant shifts, as shown in Table 1. In methanol, the open form has one main absorption peak at λ_max_ 258 nm, which diminishes after UV exposure. The closed form exhibited two new peaks at 354 and 555 nm (Figure S21). In methanol, at 2 min of irradiation, the closed form reached the highest intensity. Upon continuous exposure for 3 min, the absorption spectrum overlapped with that at 2 min. Extending irradiation to 5 min resulted in a slightly reduced intensity of the signal at 555 nm and a slight blue-shift in λ_max_. In contrast, in acetonitrile, the open form of compound **5** has one main absorption peak around λ_max_ 280 nm, and the closed form exhibited two new peaks at 378 and 610 nm (Figure 2a). The signals shifted to longer wavelengths in acetonitrile significantly and exhibited solvent-dependent photochromism. This interesting solvent-dependent photoswitching has also been observed and reported recently.^47^

**Table 1:** Λ_max_ of the Thioacids in Open and Closed Forms

compound	solvent	isosbestic point, nm	λ_max_, nm open	λ_max_, nm closed
**5**	MeOH	328	258	354, **555**
**5**	CH_3_CN	350	280	378, **610**
**6**	CH_3_CN	235, 338	255, 277	395, **628**
**7**	CH_3_CN	252, 318, 387	238, 348	310, 395, **590**
**8**	CH_3_CN	252, 368	330	393, **616**

The photochromic properties of compound **6** upon UV irradiation (302 nm) at different time points are shown in Figure 2b. The ultraviolet–visible (UV–vis) spectra are quite different compared to that of the nonfluorinated compound **5**, with λ_max_ exhibiting a significant red-shift to ∼630 nm. The closed form reached the highest intensity at around 2 min of UV irradiation, and the spectrum at 3 min almost completely overlapped with that of 2 min. Longer irradiation resulted in the reduction of the intensity of the closed form, as shown in Figure S22, with λ_max_ shifting to ∼620 nm at 5 min irradiation and ∼608 nm at 10 min. The experiment was repeated at a lower concentration, and the results are included in Figure S23. In a similar observation as mentioned above, the 10 min irradiation time resulted in a blue-shift of the absorption maximum and at reduced intensities.

**Scheme 4:**
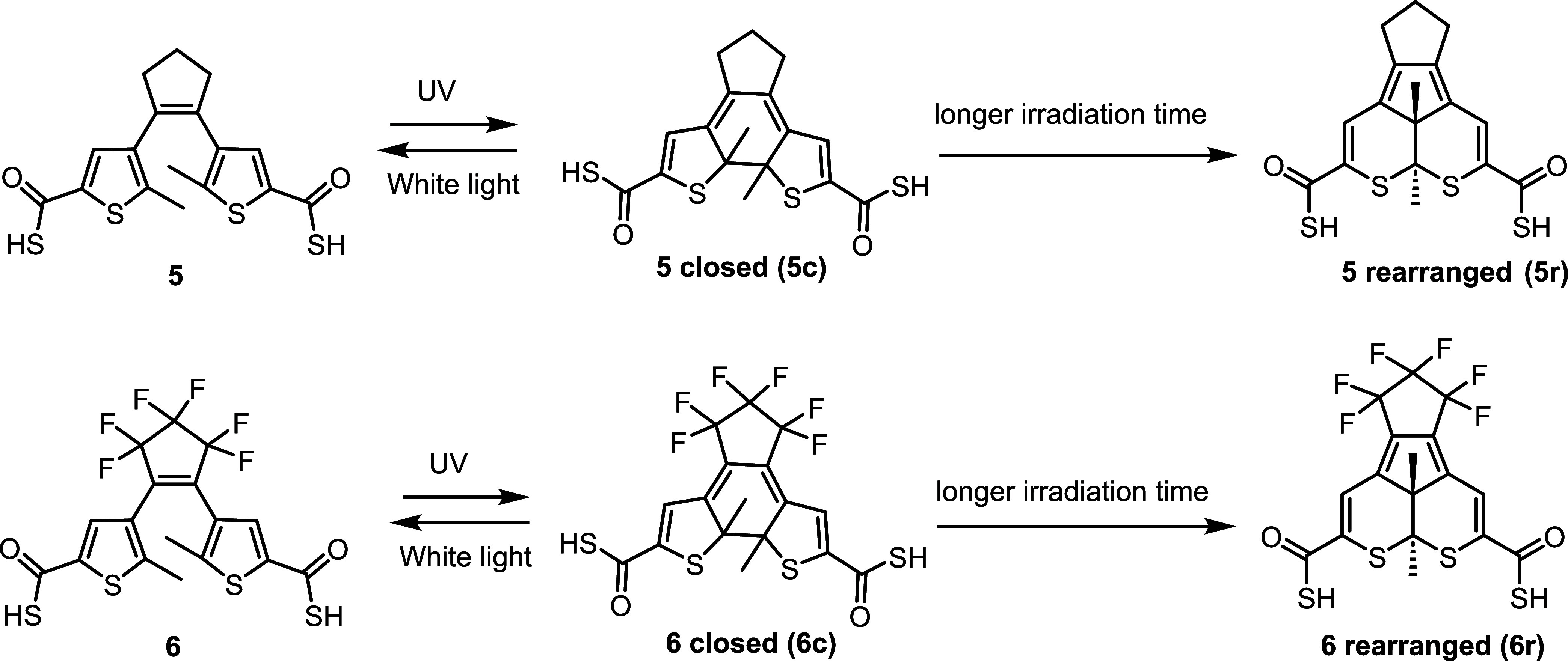
Photoswitching Properties of Compounds **5** and **6** Irradiation with UV or white light leads to the formation of reversible closed forms. Prolonged UV irradiation resulted in the formation of the rearranged annulated by-products. The by-products cannot be converted into open forms.

To further characterize the photochromic properties, we carried out fatigue resistance experiments for thioacids **5** and **6**, and the results are included in the ESI Figures S26 and S27. These two compounds exhibited reversible photoswitching properties for 10 cycles, but they were not 100% reversible. As reported by Hecht,^48,49^ Lvov,^50^ and Irie et al.,^51,52^ formation of a by-product, which is irreversible, could cause the loss of fatigue resistance. As shown in Figures S22 and S23, there is an obvious blue-shift for the closed form at a longer irradiation time. The extended irradiation may have caused the sample to rearrange into a nonreversible configuration. As shown in Scheme 4, the formation of annulated isomers **5r** and **6r** with a dihydrodithiaacenaphthlene (DDA) skeleton is irreversible to the open forms.

The thermal stability of the closed forms of these compounds was evaluated, and the results are shown in Figures S28 and S29. The UV–vis spectra of compound **5c** did not change after heating at 80 °C for 5 min and the spectra overlapped with each other, indicating that the closed form is thermally stable. On the other hand, the UV–vis spectrum of the closed form of **6c** after heating did not overlap with the sample without heating (Figure S30) and the λ_max_ shifted from ∼590 to ∼568 nm after heating, indicating that **6c** is not thermally stable. Heating will cause the peak to blue-shift, which may also indicate the possible rearrangement to form the by-product **6r** under heating conditions.^51,52^

The photoswitching properties for compounds **7** and **8**, which have a benzene spacer in the molecules, were also investigated. These compounds exhibited strong UV–vis absorptions; therefore, relatively lower concentrations were used; 0.05 and 0.025 mM in acetonitrile were used for compounds **7** and **8**, respectively. The UV–vis spectra are included in Figure 2c,d. The UV–vis absorption at different concentrations is included in Figures S24 and S25. The open form of compound **7** exhibited an absorption maximum at ∼350 nm, which diminishes upon UV exposure. The λ_max_ of the closed form at 590 nm reached the highest peak intensity at 1 min irradiation. Longer irradiation times of 2 and 3 min resulted in almost the same UV–vis spectra, and no signal reduction was observed. At a higher concentration (0.10 mM), the UV–vis spectra showed a similar trend (Figure S24), with the λ_max_ of the closed form reaching the highest intensity at 2 min irradiation and longer irradiations times of 3 and 5 min giving the same UV–vis spectra. There was no reduction in intensity or peak position shift.

The photoswitching of thioacid **8** (0.025 mM) in acetonitrile is shown in Figure 2d. The open form has a λ_max_ at 330 nm, and this signal diminished upon UV exposure. At 0.025 mM, the photocyclization reached saturation after 30 s irradiation, and the closed form has a blue color and λ_max_ at approximately 393 and 616 nm. A longer irradiation time (2 min) did not cause any change in the peak intensity and positions. At a higher concentration of 0.10 mM, approximately 3 min was required to reach the maximum intensity; longer irradiation (up to 10 min) did not cause shifts of the peaks (Figure S25).

Compared with the previously synthesized corresponding carboxylic acids, the thioacid derivatives exhibited different colors upon cyclization. Some examples of the photoswitching properties at higher concentrations upon UV irradiation are included in the figures in SI. The isosbestic points and λ_max_ of compounds **5**–**8** are summarized and included in Table 1. The closed forms for both fluorinated derivatives (**6** and **8**) exhibited a red-shift in their absorption compared to the nonfluorinated derivatives (**5** and **7**), suggesting that the fluorine substitution at the cyclopentene ring affects the energy levels.^53^

To further evaluate the photocyclization reactions, compound **5** was used as an example and its switching properties were analyzed by NMR spectroscopy. Using CDCl_3_ as the solvent, thioacid **5** was treated with UV irradiation (302 nm, 6 W) for 30 min. The ^1^H and ^13^C NMR spectra are included in ESI Figures S30 and S31. The sample exhibited partial cyclization, and after irradiation, both the photocyclized and open forms appeared on the NMR spectra. The ^13^C signal of the thioacid’s open form at 180.8 ppm shifted downfield to 183.4 ppm for the closed form. The signals indicated that thioacid functionality was stable upon UV treatment in chloroform.

However, the thioacid was not stable in DMSO, and it hydrolyzed to form the corresponding carboxylic acid instead (Figure S32). Therefore, the thioacid was converted to the corresponding potassium salt, and the stability was analyzed again. The potassium salt of compound **5** was analyzed in D_6_-DMSO, and the NMR spectra indicated that the salt was stable. As shown in Figures S33 and 34, the NMR spectra were taken after 1 week and did not show decomposition.

**Figure 3:**
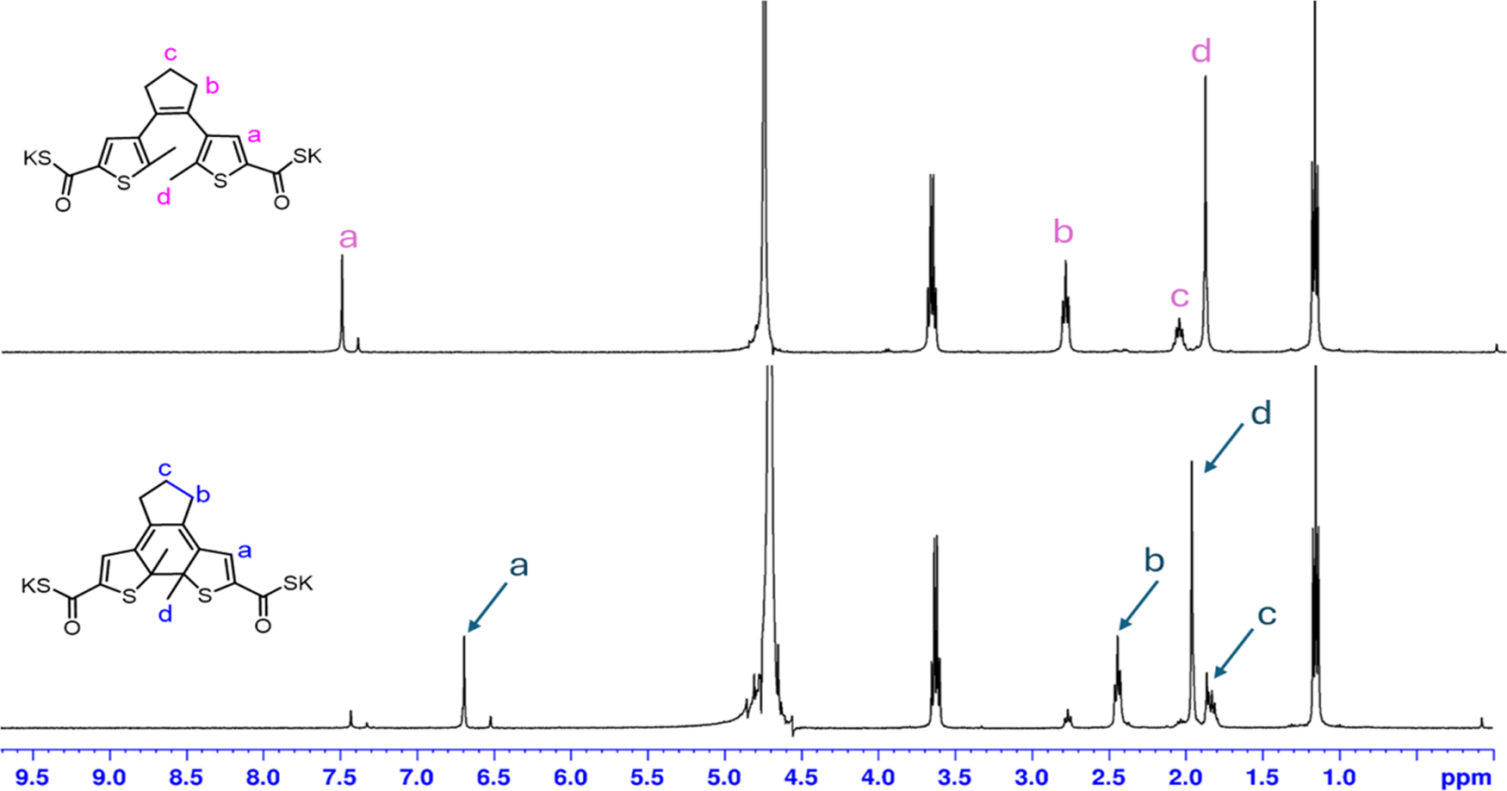
^1^H NMR spectra of the potassium salt of compound **5** in D_2_O, in the presence of ethanol, for the open and closed configurations. The bottom spectrum was obtained by irradiating the sample with UV light (302 nm, 6 W) for 60 min.

**Figure 4:**
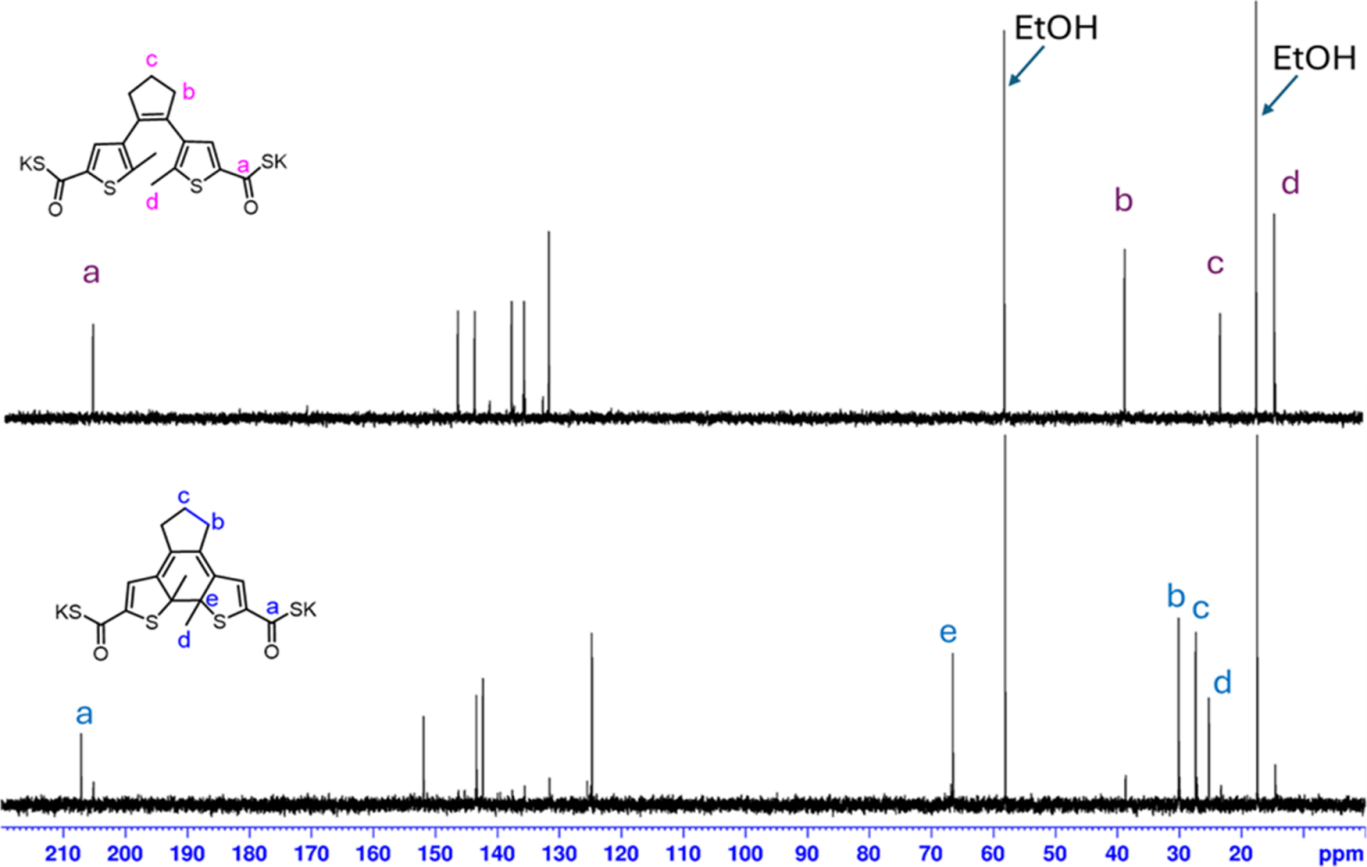
^13^C NMR (D_2_O, 400 MHz) spectrum of the potassium salt of compound **5** in D_2_O for the open-form configuration (ethanol was used as an internal standard). The bottom spectrum is of the same sample in a mostly closed configuration after irradiation with UV light (302 nm, 6W) for 60 min.

In order to analyze whether the compound can form a water-soluble photoswitch, we also prepared the potassium salt of compound **5** and acquired the NMR spectra in D_2_O. The NMR spectra before and after irradiations are shown in Figures 3 and 4. The ^1^H NMR spectra of the potassium salt in D_2_O (Figure 3) exhibited photocyclization after irradiation and a set of new signals for the closed forms. Upon UV irradiation, the anion switched to the closed form readily, with only a small amount of the open form left. The estimated ratio of the open form to the closed form after irradiation is 15:85. The COSK carbonyl signal appeared at 205.1 ppm for the open form of the salt and at 207.1 ppm for the closed form of the salt.

The photochromic properties of the potassium salt of compound **5** were also analyzed and are included in Figures S35–S40. The ionic compound (0.11 mM) in an aqueous solution exhibited photoswitching properties, with the λ_max_ of the closed form at ∼567 nm (Figure S35). Longer irradiation times did not cause a significant change in the peak intensities and positions, with only a slight blue-shift observed at 10 min irradiation. In acetonitrile, the potassium salt of compound **5** exhibited photoswitching properties, and the closed form of the salt had similar absorptions at 350 and 550 nm (Figure S36). The potassium salt of the thioacid is more stable in aqueous solutions and can be applied as a photoresponsive sensor in aqueous solutions. To expand the potential applications, we also carried out photoswitching experiments for the thioacid in the presence of different organic bases, which can form organic complexes or self-assemblies with the photoswitches. The compound exhibited similar photochromic properties upon treatment with UV light. The diisopropylethyl amine mixture is shown in Figures S37 and S38. The complex with *p*-methoxy benzylamine is shown in Figures S39 and S40. These indicate that the thioacid can be used in the presence of either organic bases or inorganic bases and form stable photochromic intermediates in aqueous solutions or in acetonitrile. This can be further explored in the future for potential applications in biomedical research as photoresponsive reporters.

We attempted to compare the photoswitching properties of compound **5** with the known compound **9b**. The quantum yield (QY) of the open to closed form for compound **9b** was reported in the literature as 0.47 with 313 nm light and 0.13 for the reversed reaction under 502 nm light.^54^ Compounds **5** and **9b** were prepared in the same molar concentrations (0.01 mM) in acetonitrile. Both were irradiated with 302 nm light and compared at the same time to estimate the photocyclization yield. The reversed closed-to-open-form conversions were also measured with white light. The UV–vis absorption spectra of compound **9b** are included in Figures S41 and S42.

The spectra of compound **5** at 0.01 mM in acetonitrile under 302 nm UV light were also acquired and are included in Figure S43. The closed form of compound **9b** reached maximum absorbance at 508 nm after 1 min UV irradiation with 302 nm. Compound **5** also reached maximum absorbance (λ_max_ at ∼600 nm) after 1 min irradiation with 302 nm light, with the assumption that both compounds have the similar ratio of closed form versus the open form when they reach the photostationary phase; therefore, the QY for the compound **5** is at the similar range of compound **9b** at 0.47.

The comparison of reversed photoconversion is shown in Figures S44 and S45. The 0.01 mM solution of compound **9b** or compound **5** was exposed to UV light of 302 nm for 1 min to obtain the fully closed forms. Then white light was passed through the cuvette for 1 min. The UV–vis spectra are shown in Figures S44 and S45. The two compounds exhibited similar extent of conversion at 1 min irradiation. Compound **5** may have similar QY to compound **9b** for the closed to open form near 0.13.

**Figure 5:**
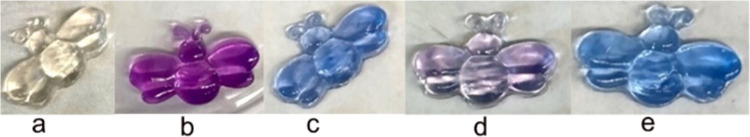
Hydrogels formed by agarose with different photoswitches. (a) Open form of compound **6**, the hydrogels after irradiation, (b) with compound **5**, (c) with compound **6**, (d) with compound **7**, and (e) with compound **8**.

Since these compounds exhibit photoswitching properties and are photochromic, they can be applied as colorimetric sensors for a variety of applications. We prepared the corresponding salts of the thioacids, which are water soluble. These compounds were then used to form hydrogels with agarose. The hydrogels exhibited photochromic properties upon treatment with UV and white light. These are included in Figures 5 and S46–S49. Before UV irradiation, the hydrogel in the open form is typically colorless and transparent. An example is shown in Figure 5a. After UV irradiation, the gels become colorful; the color intensifies with increased concentrations.

## Conclusions

In summary, we have designed and synthesized four photochromic diarylethene bis-thioacid derivatives **5**–**8**. They were synthesized from the corresponding dicarboxylic acids in two steps through an intermediate of the *N*-hydroxy succinimide derivative. The reaction of sodium hydrosulfide with the activated NHS intermediates led to the corresponding thioacids in over 90% yields. These novel thioacids exhibited interesting photoswitching and photochromic properties upon exposure to UV light or visible light. The cyclized forms of the fluorinated derivatives **6** and **8** showed a significant red-shift in their λ_max_ in comparison to the nonfluorinated derivatives **5** and **7**, respectively. The fluorinated compounds exhibit further red-shifts in their cyclized forms, indicating that aliphatic fluorine substitutions affect the photochromic properties. Compound **5** exhibited a solvent-dependent photochromism and formed water-soluble anions, as indicated by UV–vis absorptions and confirmed by NMR spectra. The stability of the thioacid **5** was characterized, and the thioacid is stable in chloroform before and after UV irradiation. The thioacid was not stable in DMSO, with the conversion to the salt resulting in stable anions in DMSO and aqueous solutions. The potassium salt of compound **5** was prepared and the open and closed forms were characterized using NMR spectroscopy in D_2_O. The complexes of compound **5** with organic bases also exhibited photoswitching properties upon treatment with UV light. The potassium salts of these thioacids were soluble in water and able to form hydrogels with agarose. The hydrogels doped with the compounds exhibited reversible color transitions upon irradiation. These thioacids are expected to be applicable for the formation of complexes with other organic or inorganic components and form photochromic and photoswitchable new materials.

## Experimental Section

### General Methods

All reactions, unless otherwise noted, were carried out in oven-dried glassware under a nitrogen atmosphere. Reagents and solvents were purchased from commercial suppliers from Sigma-Aldrich, VWR, Fisher, Ambeed, etc. and used directly without purifications. Purification was mostly conducted by flash chromatography using 230–400 mesh silica gel with a gradient of solvent systems and sometimes recrystallization. Thin-layer chromatography (TLC) analysis was performed with aluminum or glass-backed TLC plates (Sigma-Aldrich) with UV and fluorescence indicators and visualized using a UV lamp at 254 nm and stained with PMA solution. ^1^H NMR and proton-decoupled ^13^C NMR spectra were obtained with Bruker 400 MHz spectrometers in DMSO-*d*_6_ or CDCl_3_. The chemical shifts used CDCl_3_/DMSO-*d*_6_ as internal standards at 7.26:2.50 and 77.0:39.5 ppm, respectively. Some 2D NMR experiments (HSQC, COSY) were also conducted to assist the NMR assignment. UV–vis spectroscopy was done using Shimadzu, UV-1800. High-resolution mass spectra (HRMS) were obtained on a Thermo Scientific LTQ Discovery spectrometer using +ESI and reported for the molecular ion [M + Na]^+^.

### Photoswitching Experiments

The UV irradiation at 302 nm for all samples was done using a 6 W hand-held TLC lamp, model UVM-16 EL Series, with UV 302 nm and white light, which are referred to as 6 W UV light or white light, respectively. For the reverse reaction of cyclized to open form, white light from the same lamp was used for the hydrogel switching experiments. For all other visible-light irradiation experiments, a white LED spotlight lamp of 30 W and 200,000 Lumen for white light was used. This is referred to as a 30 W white LED lamp.

The detailed synthesis procedures of intermediate **13** and thioacid **5** are included here. For all other compounds, only the characterization data are included. Other experimental details are included in the SI file.

### Synthesis of Intermediate **13** and Compound **5**

#### Synthesis of Compound **13**

Compound **1** (126.2 mg, 0.36 mmol, 1.0 equiv) was dissolved in anhydrous DMF (2 mL) in a 50 mL round-bottom flask. The reaction mixture was reduced to 0 °C via an ice bath. Then, *N*-hydroxy succinimide (NHS) (166.9 mg, 1.45 mmol, 4.0 equiv) and EDC·HCl (278.0 mg, 1.45 mmol, 4.0 equiv) were added to the reaction mixture. After 15 min, the ice bath was removed, and the reaction warmed to rt and stirred for a total of 70 h. The reaction mixture was then pipetted into a stirred 0.5 M HCl solution (10 mL). A white precipitate was formed upon the addition to the reaction mixture, which was then filtered and the filtrate was extracted with DCM. The organic layers were combined and dried over Na_2_SO_4_ and the solvent was removed under reduced pressure. The crude product was purified using 0–30% acetone/hexane to obtain the desired product compound **13** as a white solid 187.6 mg (96.1%). *R*_f_ = 0.3 in 30% acetone/hexane. ^1^H NMR (400 MHz, CDCl_3_) δ 7.74 (s, 2H), 2.87 (s, 8H), 2.81 (t, *J* = 7.5, 4H), 2.1–2.0 (m, 2H), 1.99 (s, 6H); ^13^C NMR (100 MHz, CDCl_3_) δ 169.1, 157.0, 146.7, 137.3, 137.2, 134.8, 122.9, 38.8, 25.6, 22.8, 15.2.

#### Synthesis of Compound **5**

Compound **13** (150 mg, 0.28 mmol, 1 equiv) was added to a 50 mL round-bottom flask. Then, a degassed DI water–THF solution (v1:1, 5 mL) was added, followed by NaSH (62 mg, 1.1 mmol, 6 equiv). The flask was sealed with a septum and equipped with a nitrogen balloon. The reaction mixture turned dark purple within 20 min of the addition of NaSH. The reaction was monitored via TLC and ^1^H NMR. At 3 h, the starting material was no longer observed on TLC, and the ^1^H NMR spectra indicated full conversion to the desired product. The reaction was stopped, and the solvent was removed under reduced pressure. The remaining aqueous solution was acidified using a 2 M HCl solution, which produced a precipitation of a bright blue solid. The precipitate was filtered and the aqueous layer extracted with DCM. The organic layers were combined and dried over Na_2_SO_4_ and the solvent was removed under reduced pressure to obtain the desired product thioacid **5** as a blue solid of 101 mg (94.8% yield). ^1^H NMR (400 MHz, CDCl_3_) δ 7.40 (s, 2H), 2.81 (t, *J* = 7.5, 4H), 2.11–2.02 (m, 2H), 2.00 (s, 6H); ^13^C NMR (100 MHz, CDCl_3_) δ 180.8, 145.2, 137.7, 136.9, 135.0, 133.8, 38.5, 22.9, 15.1. HRMS (ESI+) *m*/*z* calcd for [C_17_H_16_O_2_S_4_Na]^+^ [M + Na]^+^: 402.9925, found 402.9896.

### Preparation of Potassium Salt of Compound **5**

Compound **5** (12 mg, 0.032 mmol, 1 equiv) was dissolved in 1 mL of EtOH in a scintillation vial and KOH (3.6 mg, 0.064 mmol, 2 equiv) was added. Then, the solution was sonicated for 15 min. Then, the solvent was removed under reduced pressure to afford a purple solid in quantitative amount. ^1^H NMR (400 MHz, H_2_O) δ 7.51 (s, 2H), 2.84 (t, *J* = 7.5, 4H), 2.16–2.06 (m, 2H), 1.94 (s, 6H); ^13^C NMR (100 MHz, D_2_O) δ 205.1, 146.2, 143.4, 137.5, 135.5, 131.5, 38.6, 23.2, 14.4.

The sample was treated with 302 nm UV light to afford the closed form: ^1^H NMR (400 MHz, H_2_O) δ 6.77 (s, 2H), 2.52 (t, *J* = 7.5, 4H), 2.04 (s, 6H), 1.96–1.86 (m, 2H); ^13^C NMR (100 MHz, H_2_O) δ 207.1, 151.9, 143.3, 142.3, 124.9, 66.5, 30.0, 27.3, 25.2.

### Synthesis of Intermediate **14** and Compound **6**

#### Synthesis of Compound **14**

The crude compound **14** was purified by column chromatography using 0–30% acetone/hexanes to afford light blue solid as the desired product **14** in 113 mg (79.2%) yield. *R*_f_ = 0.2 in 30% acetone/hexanes. ^1^H NMR (400 MHz, CDCl_3_) δ 7.98 (s, 2H), 2.90 (s, 8H), 2.06 (s, 6H); ^13^C NMR (100 MHz, CDCl_3_) δ 168.8, 156.3, 152.0, 136.0, 125.9, 125.6, 25.6, 15.3.

#### Synthesis of Compound **6**

Compound **14** (53 mg, 0.08 mmol, 1 equiv), THF–water solution (3 mL), and NaSH (18 mg, 0.32 mmol, 4 equiv) were used. The product compound **6** was obtained as a greenish solid in 36 mg yield (92.3%). ^1^H NMR (400 MHz, CDCl_3_) δ 7.65 (s, 2H), 2.02 (s, 6H); ^13^C NMR (100 MHz, CDCl_3_) δ 180.6, 150.4, 140.0, 132.0, 125.6, 15.2; HRMS (ESI+) *m*/*z* calcd for [C_17_H_10_F_6_O_2_S_4_Na]^+^ [M + Na]^+^: 510.9366, found 510.9357.

### Synthesis of Intermediate **17** and Compound **7**

#### Synthesis of Compound **17**

The synthesis of compound **3** from compound **9a** and the detailed procedures for the preparation of compound **7** are provided in the ESI. The crude product **17** was purified by flash chromatography using 50–60% acetone/hexane, and the pure intermediate **17** was obtained as a blue solid in 168 mg (83%) yield. *R*_f_ = 0.2 in 30% acetone/hexane. ^1^H NMR (400 MHz, CDCl_3_) δ 8.08 (d, *J* = 8.6 Hz, 4H), 7.69 (d, *J* = 8.6 Hz, 4H), 7.21 (s, 2H), 2.90 (s, 8H), 2.86 (t, *J* = 7.6 Hz, 4H), 2.15–2.07 (m, 2H), 2.04 (s, 6H); ^13^C NMR (100 MHz, CDCl_3_) δ 169.3, 161.5, 140.5, 137.9, 137.3, 137.2, 134.8, 131.2, 126.2, 125.0, 122.9, 38.4, 25.7, 23.0, 14.6.

#### Synthesis of Compound **7**

Compound **17** (100 mg, 0.14 mmol, 1 equiv), THF–water solution (5 mL), and NaSH (32 mg, 0.58 mmol, 4 equiv) were used. The product compound **7** was obtained as a bright blue solid (72 mg), 96.5%. ^1^H NMR (400 MHz, CDCl_3_) δ ppm, 7.85 (d, *J* = 8.5 Hz, 4H), 7.54 (d, *J* = 8.5 Hz, 4H), 7.14 (s, 2H), 2.85 (t, *J* = 7.4 Hz, 4H), 2.15–2.06 (m, 2H), 2.03 (s, 6H). ^13^C NMR (100 MHz, CDCl_3_) δ 189.1, 139.7, 138.0, 137.2, 137.0, 134.9, 134.7, 128.7, 126.0, 125.1, 38.4, 23.0, 14.6; HRMS (ESI+) *m*/*z* calcd for [C_29_H_24_O_2_S_4_Na]^+^ [M + Na]^+^: 555.0551, found 555.0521.

### Synthesis of Intermediate **18** and Compound **8**

#### Synthesis of Compound **18**

The perfluorinated diarylethene thioacid **8** was prepared using compound **9b**. The synthesis of compound **4** from compound **9b** and the detailed procedures for the preparation of compound **8** are provided in the ESI. The crude product **18** was purified by using column chromatography using 0–30% acetone/hexane to obtain a greenish solid in 110 mg yield (83.3%). *R*_f_ = 0.3 in 30% acetone/hexane. ^1^H NMR (400 MHz, CDCl_3_) δ 8.14 (d, *J* = 8.7, 4H), 7.66 (d, *J* = 8.7, 4H), 7.43 (s, 2H), 2.91 (s, 8H), 2.03 (s, 6H). ^13^C NMR (100 MHz, CDCl_3_) δ: 169.1, 161.4, 143.6, 140.5, 139.3, 131.4, 126.3, 125.5, 124.6, 124.1, 25.7, 14.7.

#### Synthesis of Compound **8**

Compound **18** (75 mg, 0.09 mmol, 1 equiv), THF–water solution (5 mL), and NaSH (21 mg, 0.37 mmol, 4 equiv) were used. Compound **8** was obtained as a blue solid in 55 mg yield (94.8%). ^1^H NMR (400 MHz, CDCl_3_) δ 7.91 (d, *J* = 8.5, 4H), 7.62 (d, *J* = 8.5, 4H), 7.39 (s, 2H), 2.01 (s, 6H); ^13^C NMR (100 MHz, CDCl_3_) δ 189.1, 143.3, 140.6, 138.4, 135.6, 131.0, 128.8, 126.2, 125.5, 124.2, 14.7; HRMS (ESI+) *m*/*z* calcd for [C_29_H_18_F_6_O_2_S_4_Na]^+^ [M + Na]^+^: 662.9986, found 662.9944.

## Supporting Information Available

Experimental procedure and characterization data; copies of ^1^H and ^13^C NMR spectra of compounds **3**–**8** and **11**–**18**; the UV–vis spectra of compounds **5**–**8** at different UV irradiation times; UV–vis spectra of the potassium salt of compound **5**; fatigue resistance study; additional NMR spectra for the stability studies; and images of agarose hydrogels formed in the presence of the thioacids (PDF)
