# Supplementary material for: Synthesis of Bis-Thioacid Derivatives of Diarylethene and Their Photochromic Properties
Source: ACS Omega. 2024 Nov 18;9(48):47489–99. doi: 10.1021/acsomega.4c05945 (PMC11618401; doi:10.1021/acsomega.4c05945)
Supplement: Supplementary file 1 — ao4c05945_si_001.pdf [file ao4c05945_si_001.pdf]

Supplementary Information

Synthesis of Bis-thioacid Derivatives of Diarylethene and Their Photochromic Properties

Pramod Aryal[a], Jonathan Bietsch[a], Gowri Sankar Grandhi[a], Richard Chen[a], Surya B. Adhikari[a], Ephraiem S. Sarabamoun[b], Joshua J. Choi[b], and Guijun Wang[a]*

[a.] Department of Chemistry and Biochemistry, Old Dominion University, Norfolk, VA 23529.

[b.] Department of Chemical Engineering, University of Virginia, Charlottesville, VA, United States.

Content | Page

	Content	Page
I.	Synthesis and characterization of compounds **3-8** and **11-18**	S2
II.	NMR spectra for compounds **3-8** and **11-18**	S10
	• ^{1}H and ^{13}C NMR spectra of **3-8**, **11-18**	S10
	• 2D NMR spectra of compounds **5-8**	S26
III.	Photo switching experiments for compounds **5-8**	S29
IV.	Fatigue resistance studies for compounds **5** and **6**	S34
V.	Stability of thioacids analyzed by NMR spectroscopy	S39
VI.	The UV-Vis spectra of compound **5** with different bases	S45
VII.	Comparison of photoswitching of compounds **5** and **9b**	S49
VIII.	Photochromic hydrogels prepared using compounds **5-8**	S55

I. Synthesis and characterization of compounds 3-8, 11-18

1. Synthesis of compound **11**

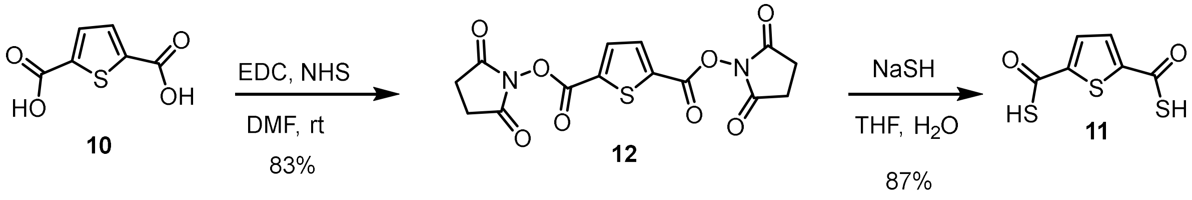

Scheme S1. Synthesis of diarylethene thioacid **11**

*Synthesis of Compound **12*** Compound **10** (400 mg, 2.32 mmol, 1.0 equiv.) was dissolved in anhydrous DMF (5 mL) in a 50 mL round bottomed flask. The reaction mixture was reduced to 0 °C via an ice bath and N-hydroxy succinimide (NHS) (801 mg, 6.96 mmol, 3.0 equiv.) and EDC·HCl (1.334 g, 6.96 mmol, 3.0 equiv.) was added to the reaction mixture. After 15 minutes, the ice bath was removed, and the reaction warmed to room temperature (rt) and stirred for a total of 70 hours. The reaction mixture was then pipetted into a stirring 0.5 M HCl solution (20 mL). A white precipitate formed upon the addition to the reaction mixture, which was then filtered, and 10 mL acetone was added to the solid making slurry which was filtered to get white solid as desired product compound **12** 703 mg (83%). ^{1}H NMR (400 MHz, $CDCl_3$) δ ppm 8.01 (s, 2H), 2.91 (s, 8H); ^{13}C NMR (100 MHz, $CDCl_3$) δ ppm 168.5, 156.5, 135.9, 134.8, 25.6.

*Synthesis of Compound **11*** Compound **12** (200 mg, 0.55 mmol, 1.0 equiv.) was added to a 50 mL round bottomed flask. Then Degassed DI water (5 mL) was added, followed by NaSH (189 mg, 3.36 mmol, 6.0 equiv.). The flask was sealed with a septum and equipped with a nitrogen balloon. The reaction was monitored by ^{1}H NMR. At 3 hours, the starting material was no longer observed on ^{1}H NMR spectra indicated full conversion to the desired product. The reaction was stopped,

and acidified using 2M HCl solution, which produced a precipitation of a yellow solid. The precipitate was filtered, and the aqueous layer was then extracted with DCM. The organic layers were combined, dried over Na_2SO_4 filtered and the solvent was removed under reduced pressure to obtain desired product thioacid **11** as a yellow solid of 97 mg, (87% yield).; ^{1}H NMR (400 MHz, $CDCl_3$) δ ppm 7.68 (s, 2H); ^{13}C NMR (100 MHz, $CDCl_3$) δ ppm 181.7, 147.1, 132.0.

2. Synthesis of compound **6**

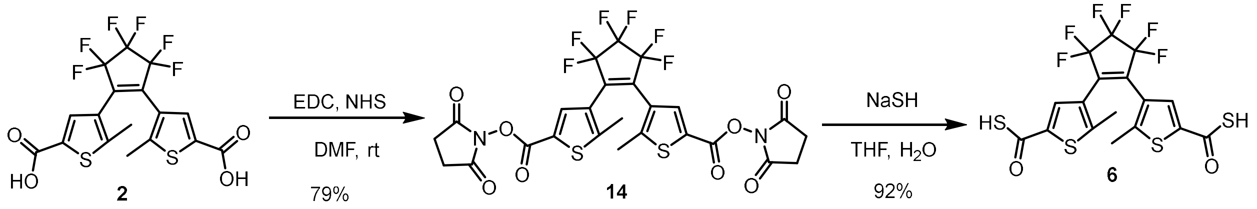

Scheme S2. Synthesis of perfluorinated diarylethene thioacid **6**

*Synthesis of Compound **14*** Compound **2** (100 mg, 0.22 mmol, 1.0 equiv.) was dissolved in anhydrous DMF (3 mL) in a 50 mL round bottomed flask. The reaction mixture was cooled to 0 °C via an ice bath, NHS (76 mg, 0.66 mmol) and EDC·HCl (127 mg, 0.66 mmol, 3.0 equiv.) was added to the reaction mixture. The reaction was allowed to stir for 72 hours. Then the mixture was quenched with 0.5 M HCl 5 mL ice water and extracted with ethyl acetate. The organic layers were combined, dried over Na_2SO_4, filtered, and the solvent was removed under reduced pressure. The crude was further purified by column chromatography using 0-30% Acetone/Hexanes to afford light blue solid as a desired product **14**, in 113 mg (79%) yield. $R_f = 0.2$ in 30% acetone/hexanes. ^{1}H NMR (400 MHz, $CDCl_3$) δ 7.98 (s, 2H), 2.90 (s, 8H), 2.07 (s, 6H); ^{13}C NMR (100 MHz, $CDCl_3$) δ 168.8, 156.3, 152.0, 136.0, 125.9, 125.6, 25.6, 15.3.

*Synthesis of Compound **6*** Compound **14** (53 mg, 0.08 mmol, 1.0 equiv.) was added to a 50 mL round-bottomed flask. Degassed DI water-THF solution (3 mL) was added, followed by NaSH (18 mg, 0.32 mmol, 4.0 equiv.). The flask was sealed with a septum and equipped with a nitrogen balloon. At 3 hours, the starting material was no longer observed. The reaction was stopped, and solvent was removed under reduced pressure to remove THF and the remaining aqueous solution was acidified using 3M HCl. A greenish solid precipitated and it was filtered. The aqueous layer was further extracted with DCM, the organic phase was dried over Na_2SO_4, filtered, and the solvent was removed under reduced pressure to give additional product as a greenish solid, the total product **6** obtained was 36 mg (92%). ^{1}H NMR (400 MHz, $CDCl_3$) δ 7.65 (s, 2H), 2.01 (s, 6H); ^{13}C NMR (100 MHz, $CDCl_3$) δ 180.6, 150.4, 140.0, 132.0, 125.6, 15.2; HRMS (ESI+) *m/z* calcd for $[C_{17}H_{10}F_6O_2S_4Na]^+$ $[M + Na]^+$: 510.9366, found 510.9357.

3. Synthesis of compound **7**

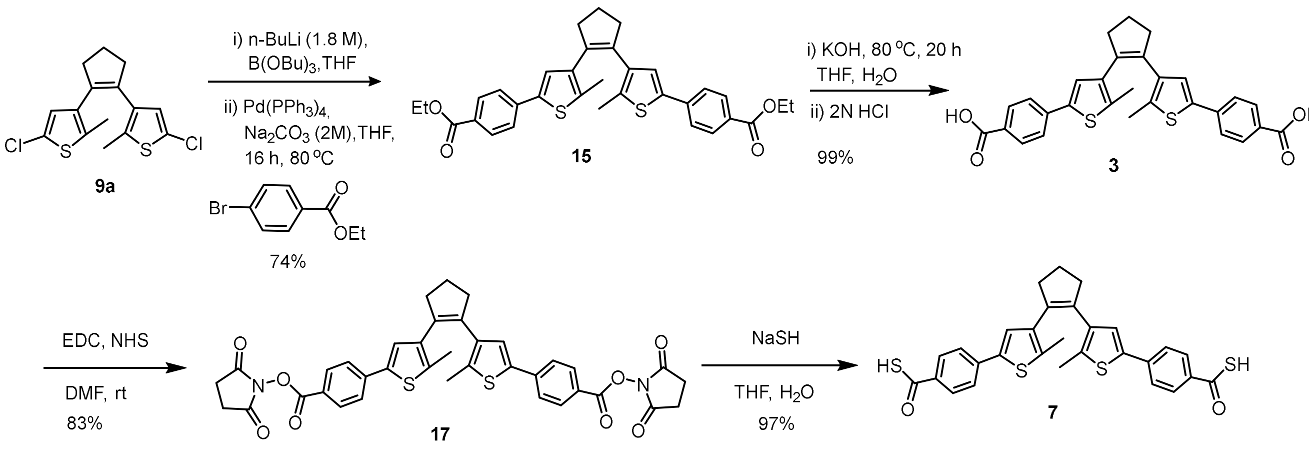

Scheme S3. Synthesis of diarylethene thioacid **7**

*Synthesis of Compound **15*** Compound **9a** (600 mg, 1.82 mmol, 1.0 equiv.) was dissolved in THF (10 mL) in a 50 mL RBF under nitrogen, n-BuLi (2.7 mL, 4.92 mmol, 2.7 equiv.) was added

dropwise at 0 °C. The solution was stirred at r.t. for 30 min., $B(OBu)_3$ (1.57 mL, 5.46 mmol, 3.0 equiv.) was added to the flask and stirred at rt for another 1 h. In another flask, ethyl 4-bromobenzoate (1.25 g, 5.47 mmol, 3.0 equiv.) was dissolved in THF (5 mL) and $Pd(PPh_3)_4$ (210 mg, 0.18 mmol, 0.1 equiv.) was added. The reaction was stirred at rt for 15 min then heated to 80 °C for 30 min. Finally, 15 mL of an aqueous solution of 2M Na_2CO_3 and the previously prepared borylated solution were added to the flask, and the reaction mixture was stirred at 80 °C for 16 h. TLC was used to monitor the reaction and indicated completion of the reaction. The mixture was quenched with water and extracted with EtOAc. The organic layer was dried over Na_2SO_4. The crude product was purified by column chromatography using 2-3% EtOAc/Hexane and 750 mg (74% yield) product **15** was obtained, R_f = 0.3 in 5% EtOAc/Hexanes. ^{1}H NMR (400 MHz, $CDCl_3$) δ 7.99 (d, J = 8.5 Hz , 4H), 7.53 (d, J = 8.5 Hz, 4H), 7.12 (s, 2H), 4.35 (q, J = 7.1 Hz, 4H), 2.85 (t, J = 7.4 Hz, 4H), 2.15-2.05 (m, 2H), 2.02 (s, 6H), 1.39 (t, J = 7.1 Hz, 6H) ; ^{13}C NMR (100 MHz, $CDCl_3$) δ 166.3, 138.58, 138.56, 137.0, 136.2, 134.8, 130.2, 128.7, 125.4, 124.8, 60.9, 38.5, 23.0, 14.5, 14.3.

*Synthesis of Compound **3*** Compound **15** (100 mg, 0.18 mmol) was dissolved in THF (5 mL) and H_2O (5 mL) in a 50 mL round bottom flask, then KOH (100 mg) was added. This reaction mixture was stirred at 80 °C for 12 hours. After which the hydrolysis was completed as indicated by TLC. The reaction mixture was acidified with 2N HCl and the precipitate was filtered through a Buchner funnel and dried to obtain a white solid as the product **3**, yield 80 mg (99%). ^{1}H NMR (400 MHz, DMSO-d_6) δ 12.88 (s, 2H), 7.91 (d, J = 8.5 Hz, 4H), 7.64 (d, J = 8.5 Hz, 4H), 7.44 (s, 2H), 2.86 (t, J = 7.4 Hz, 4H), 2.11-2.01 (m, 2H), 1.94 (s, 6H). ^{13}C NMR (100 MHz, DMSO-d_6) 167.3, 138.3, 138.1, 137.5, 135.8, 134.8, 130.6, 129.5, 126.5, 125.1, 38.6, 22.8, 14.6.

Synthesis of Compound 17 Compound **3** (145 mg, 0.29 mmol, 1.0 equiv.) in DMF (3 mL), NHS (100 mg, 0.87 mmol, 3.0 equiv.) and EDC·HCl (166 mg, 0.87 mmol, 3.0 equiv.) was added and the reaction mixture was stirred at RT for 72 hours. The reaction mixture was quenched with ice cold water and extracted with ethyl acetate. The organic layer was dried over Na_2SO_4, and the solvent was removed to afford the crude product. The crude product was purified by flash chromatography using 50-60% acetone/hexane, the intermediate **17** was obtained as a blue solid in 168 mg 83% yield, $R_f = 0.2$ in 30% Acetone/Hexane. ^1H NMR (400MHz, $CDCl_3$) δ 8.08 (d, J = 8.6 Hz , 4H), 7.69 (d, J = 8.6 Hz, 4H), 7.21 (s, 2H), 2.90 (s, 8H), 2.86 (t, J = 7.6 Hz, 4H), 2.15-2.07 (m, 2H), 2.04 (s, 6H) ; ^{13}C NMR (100 MHz, $CDCl_3$) δ 169.3, 161.5, 140.5, 137.9, 137.3, 137.2, 134.8, 131.2, 126.2, 125.0, 122.9, 38.4, 25.7, 23.0, 14.6.

Synthesis of Compound 7 Compound **17** (100 mg, 0.14 mmol, 1.0 equiv.) was added to a 50 mL round-bottomed flask. Degassed DI water-THF solution (5 mL) was added, followed by NaSH (32 mg, 0.58 mmol, 4.0 equiv.). The flask was sealed with a septum and equipped with a nitrogen balloon. The reaction turned dark purple within 20 minutes of the addition of NaSH. At 3 hours, the starting material was no longer observed on TLC and ^1H NMR. The flask was placed on the rotovap at 30 °C for 30 minutes to remove THF and then the remaining aqueous solution was acidified using 2 M HCl causing the precipitation of a bright blue solid. The solid precipitate was filtered, and the aqueous layer was then extracted with DCM (10 mL x 3). The organic layers were combined, dried over Na_2SO_4 filtered and the solvent was removed under reduced pressure to afford compound **7**, 72 mg, 97%. ^1H NMR (400 MHz, $CDCl_3$) δ ppm, 7.85 (d, J = 8.5 Hz, 4H), 7.54 (d, J = 8.5 Hz, 4H), 7.14 (s, 2H), 2.85 (t, J = 7.4 Hz, 4H), 2.15-2.06 (m, 2H), 2.03 (s, 6H). ^{13}C

NMR (100 MHz, $CDCl_3$) δ 189.1, 139.7, 138.0, 137.2, 137.0, 134.9, 134.7, 128.7, 126.0, 125.1, 38.4, 23.0, 14.6; HRMS (ESI+) *m/z* calcd for $[C_{29}H_{24}O_2S_4Na]^+$ $[M + Na]^+$: 555.0551, found 555.0521.

4. Synthesis of compound **8**

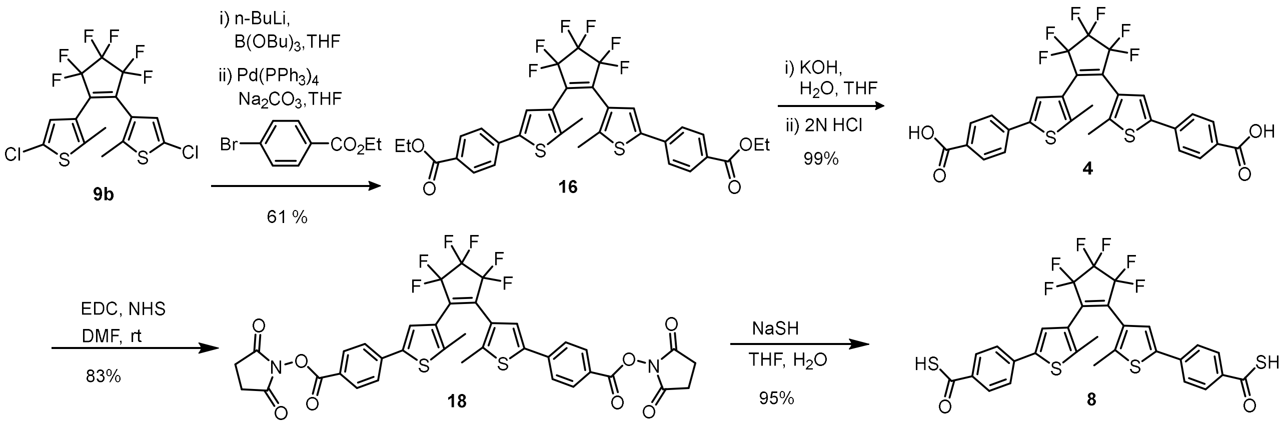

Scheme S4. Synthesis of perfluorinated diarylethene thioacid **8**

*Synthesis of Compound **16*** Compound **9b** (50 mg, 0.11 mmol) was dissolved in Et_2O (5 mL) in a 50 mL RBF under nitrogen, n-BuLi (0.17 mL, 0.1 mmol) was added dropwise at 0 °C. The solution was stirred at rt for 30 minutes. Then, $B(OBu)_3$ (0.01 mL, 0.34 mmol) was added in one portion and the reaction stirred at rt for 1h. In another flask, ethyl 4-bromobenzoate (78 mg, 0.34 mmol) was dissolved in THF (5 mL) and $Pd(PPh_3)_4$ (13 mg, 0.01 mmol) was added and reaction was stirred at rt for 15 min then heated to 80 °C. Finally, an aqueous solution of Na_2CO_3 (2.5 mL 2M) and previously prepared borylated solution were added and the reaction mixture was stirred at 80 °C for 16 h. The reaction was quenched with water and extracted with EtOAc. The organic layer was dried over Na_2SO_4. The crude product was purified by column chromatography using 2-3% ethyl acetate/hexane to obtain compound **16**, 46 mg, 61% (R_f = 0.2 in 5% EtOAc/Hexane). ^{1}H

NMR (400 MHz, $CDCl_3$) δ 8.04 (d, J = 8.5 Hz, 4H), 7.58 (d, J = 8.5 Hz, 4H), 7.37 (s, 2H), 4.39 (q, J = 7.1 Hz, 4H), 2.00 (s, 6H), 1.40 (t, J = 7.1 Hz, 6H); ^{13}C NMR (100 MHz, $CDCl_3$) δ 166.1, 142.7, 141.1, 137.3, 130.3, 129.7, 126.1, 125.2, 123.8, 61.1, 14.7, 14.3.

*Synthesis of Compound **4*** Compound **16** (275 mg, 0.41 mmol) was added to a flask, then THF (5 mL), H_2O (5 mL), and KOH (300 mg) were added. This reaction was stirred at 80 °C for 16 hours. After 16 hours, progress of reaction was monitored by TLC, TLC showed consumption of ester starting material. The reaction mixture was acidified with 2N HCl and product filtered to obtain compound **4** in 250 mg of desired product (99%). ^{1}H NMR (400 MHz, DMSO-d_6) δ 13.00 (s, 2H), 7.97 (d, J = 8.6, 4H), 7.77 (d, J = 8.6, 4H), 7.67 (s, 2H), 2.02 (s, 6H). ^{13}C NMR (100 MHz, DMSO-d_6) δ 166.7, 142.7, 140.5, 136.4, 136.2, 130.2, 129.9, 125.2, 124.2, 14.1.

*Synthesis of Compound **18*** Compound **4** (100 mg, 0.16 mmol, 1.0 equiv.) was dissolved in anhydrous DMF (3 mL) in a 50 mL round bottomed flask. The reaction mixture was cooled to 0 °C via an ice bath, NHS (56 mg, 0.49 mmol, 3.0 equiv.) and EDC·HCl (94 mg, 0.49 mmol, 3.0 equiv.) were added to the mixture. The reaction was allowed to stir for 70 hours at rt. The reaction mixture was then pipetted into a stirring 0.5 M HCl solution (10 mL). A precipitate was formed upon the addition of the reaction mixture, forming a slurry. The aqueous slurry was extracted with EtOAC (10 mL x3). The organic layers were combined, dried over Na_2SO_4, filtered, and the solvent was removed under reduced pressure. Crude product was purified by using column chromatography using 0-30% Acetone/Hexane to obtain a greenish solid as the product **18**, 110 mg (83%). R_f = 0.3 in 30% acetone/hexanes. ^{1}H NMR (400 MHz, $CDCl_3$) δ 8.14 (d, J = 8.7, 4H),

7.66 (d, $J = 8.7$, 4H), 7.43 (s, 2H), 2.91 (s, 8H), 2.03 (s, 6H). ^{13}C NMR (100 MHz, $CDCl_3$) δ 169.1, 161.4, 143.6, 140.5, 139.3, 131.4, 126.3, 125.5, 124.6, 124.1, 25.7, 14.7.

*Synthesis of Compound **8*** Compound **18** (75 mg, 0.09 mmol, 1.0 equiv.) was added to a 50 mL round-bottomed flask. Degassed DI water-THF solution (5 mL) was added, followed by NaSH (21 mg, 0.37 mmol, 4.0 equiv.). The flask was sealed with a septum and equipped with a nitrogen balloon. The reaction was monitored via TLC and ^{1}H NMR. At 3 hours, the starting material was no longer observed on TLC and ^{1}H NMR. The solvent was then removed under reduced pressure and remaining aqueous solution was acidified using 2 M HCl causing the precipitation of a bright blue solid. The solid precipitate was filtered, and the aqueous layer was then extracted with DCM (10 mL x 3). The organic layers were combined, dried over Na_2SO_4 filtered and the solvent was removed under reduced pressure to obtain compound **8** in total 55 mg (95%) blue solid. ^{1}H NMR (400 MHz, $CDCl_3$) δ 7.91 (d, $J = 8.5$, 4H), 7.62 (d, $J = 8.5$, 4H), 7.39 (s, 2H), 2.01 (s, 6H); ^{13}C NMR (100 MHz, $CDCl_3$) δ 189.1, 143.3, 140.6, 138.4, 135.6, 131.0, 128.8, 126.2, 125.5, 124.2, 14.7; HRMS (ESI+) *m/z* calcd for $[C_{29}H_{18}F_6O_2S_4Na]^+$ $[M + Na]^+$: 662.9986, found 662.9944.

II. NMR spectra for compounds **3-8** and **11-18**

All done using Bruker 400 MHz NMR machine, 400 MHz for ^{1}H, and 100 MHz for ^{13}C

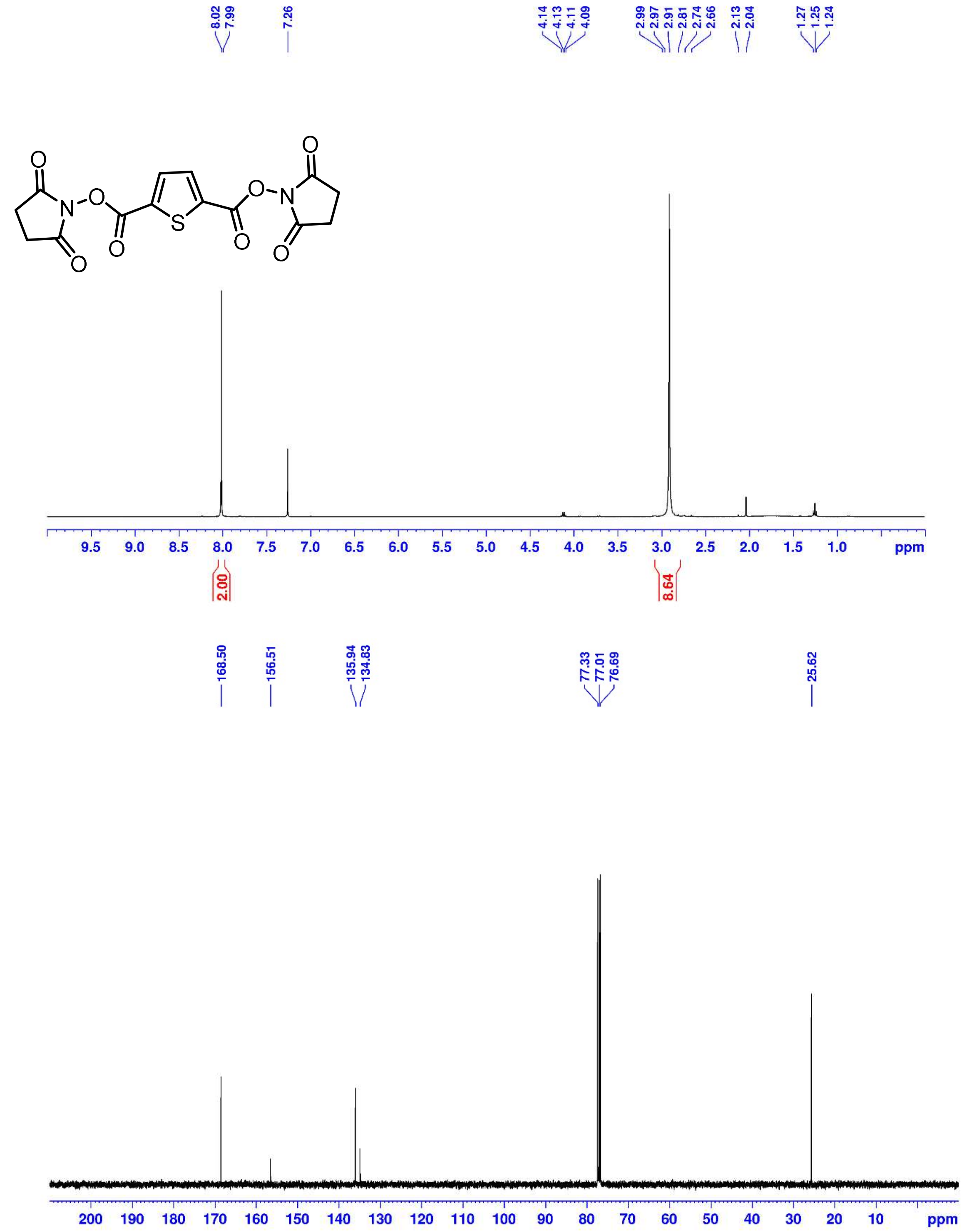

Figure S1. ^{1}H and ^{13}C NMR spectra of compound **12** in $CDCl_3$

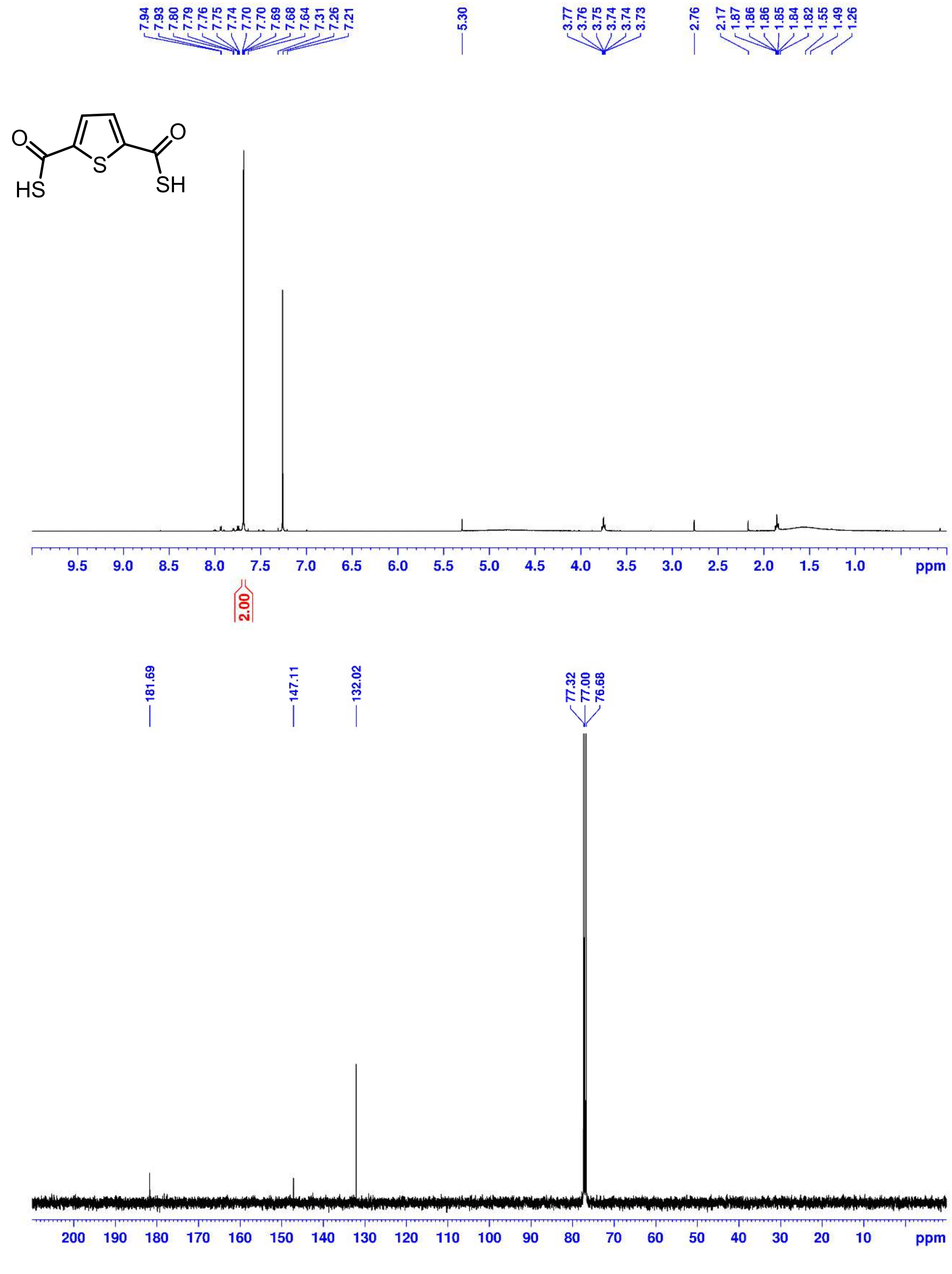

Figure S2. ^{1}H and ^{13}C NMR spectra of compound **11** in $CDCl_3$

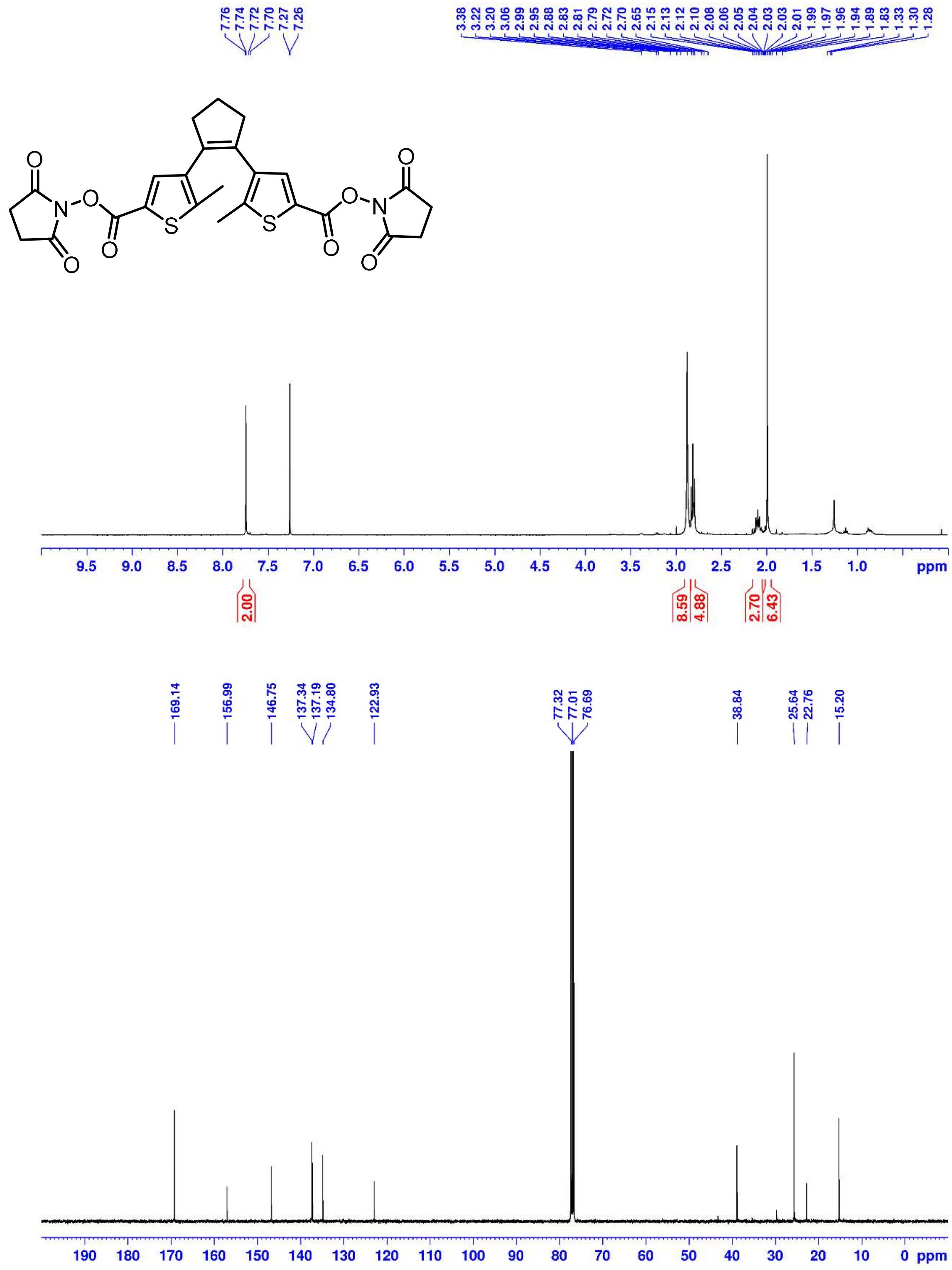

Figure S3. ^{1}H and ^{13}C NMR spectra of compound **13** in $CDCl_3$

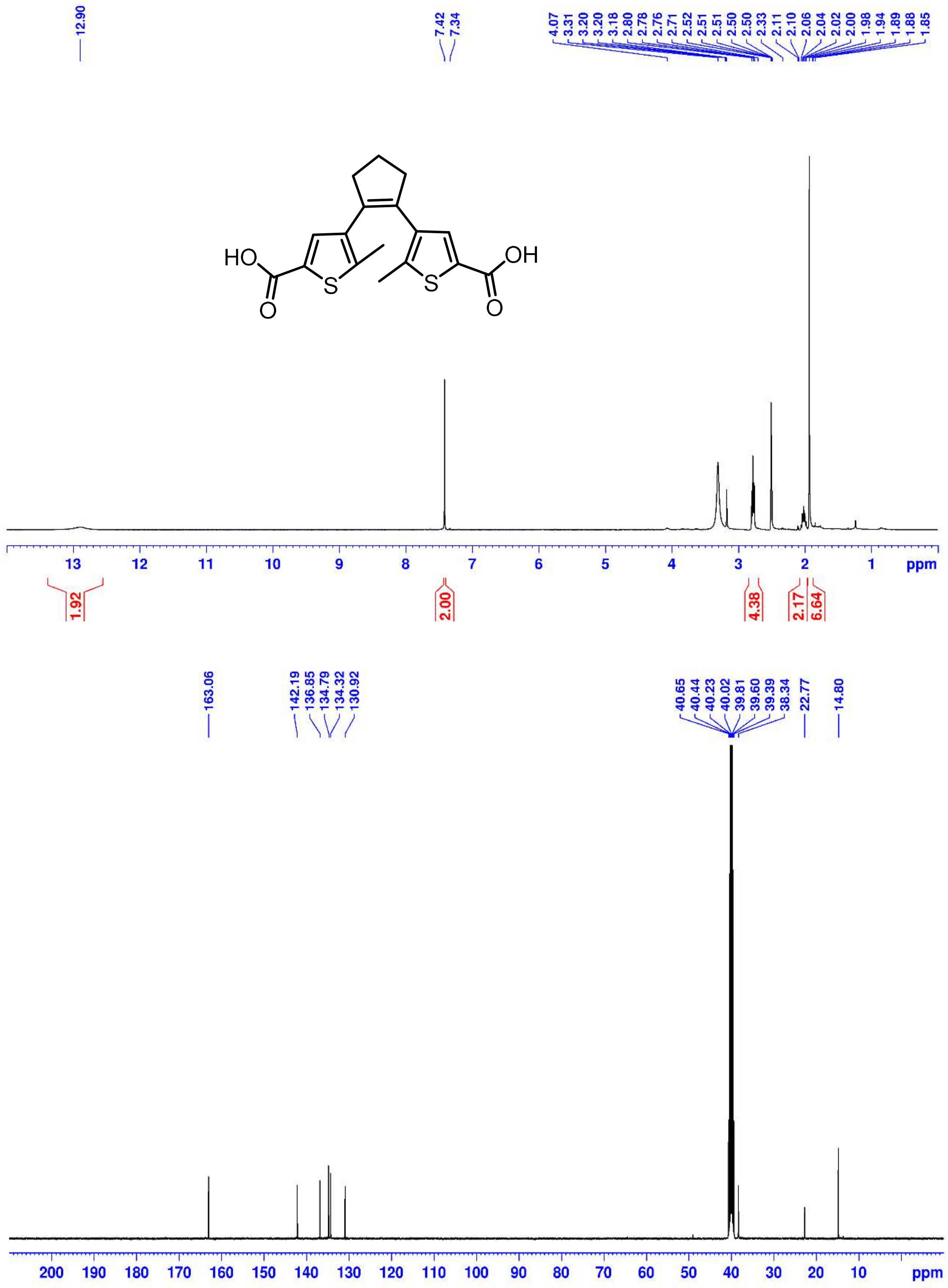

Figure S4. ^{1}H and ^{13}C NMR spectra of compound **1** in DMSO-d_6

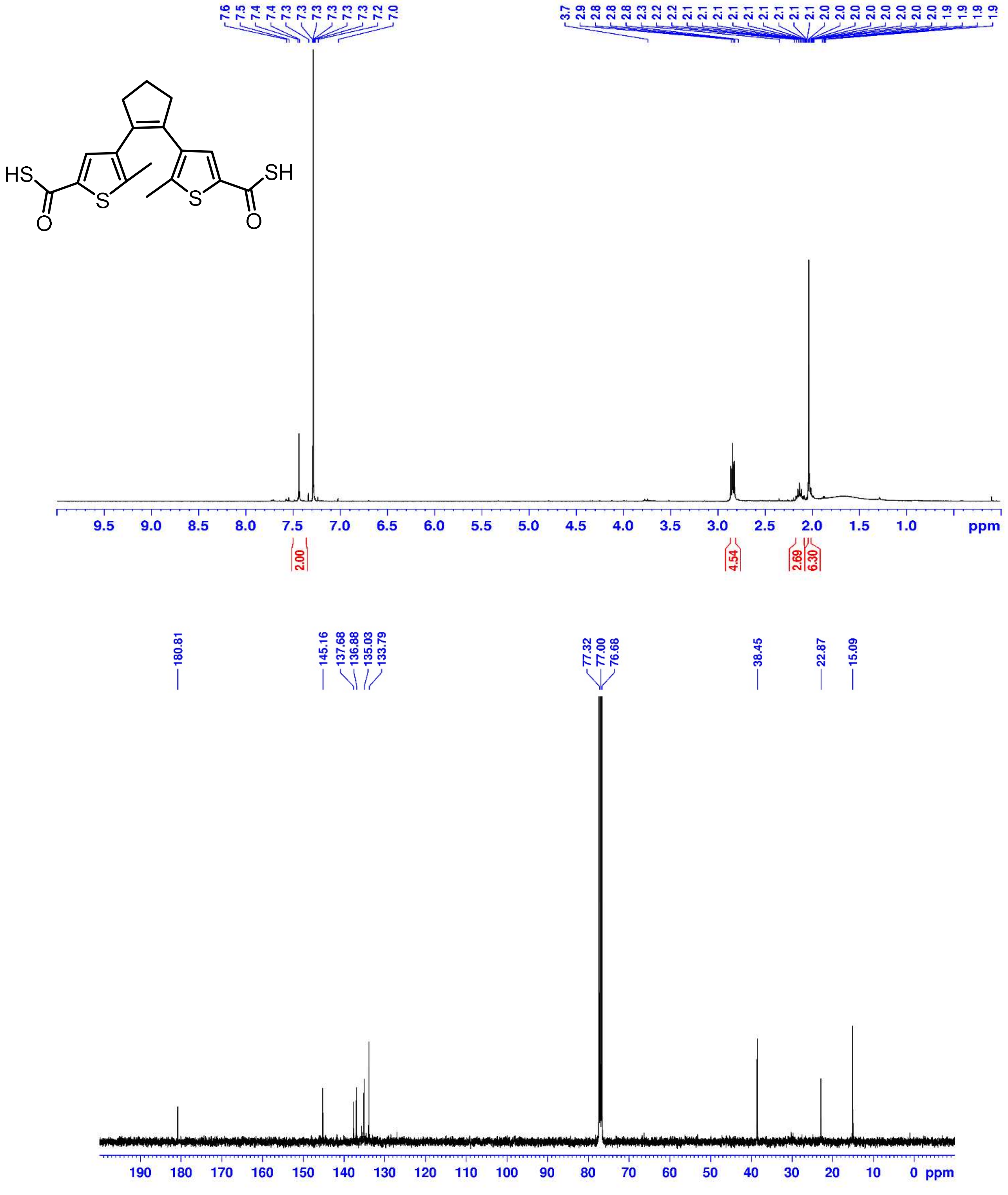

Figure S5. ^{1}H and ^{13}C NMR spectra of compound **5** in $CDCl_3$

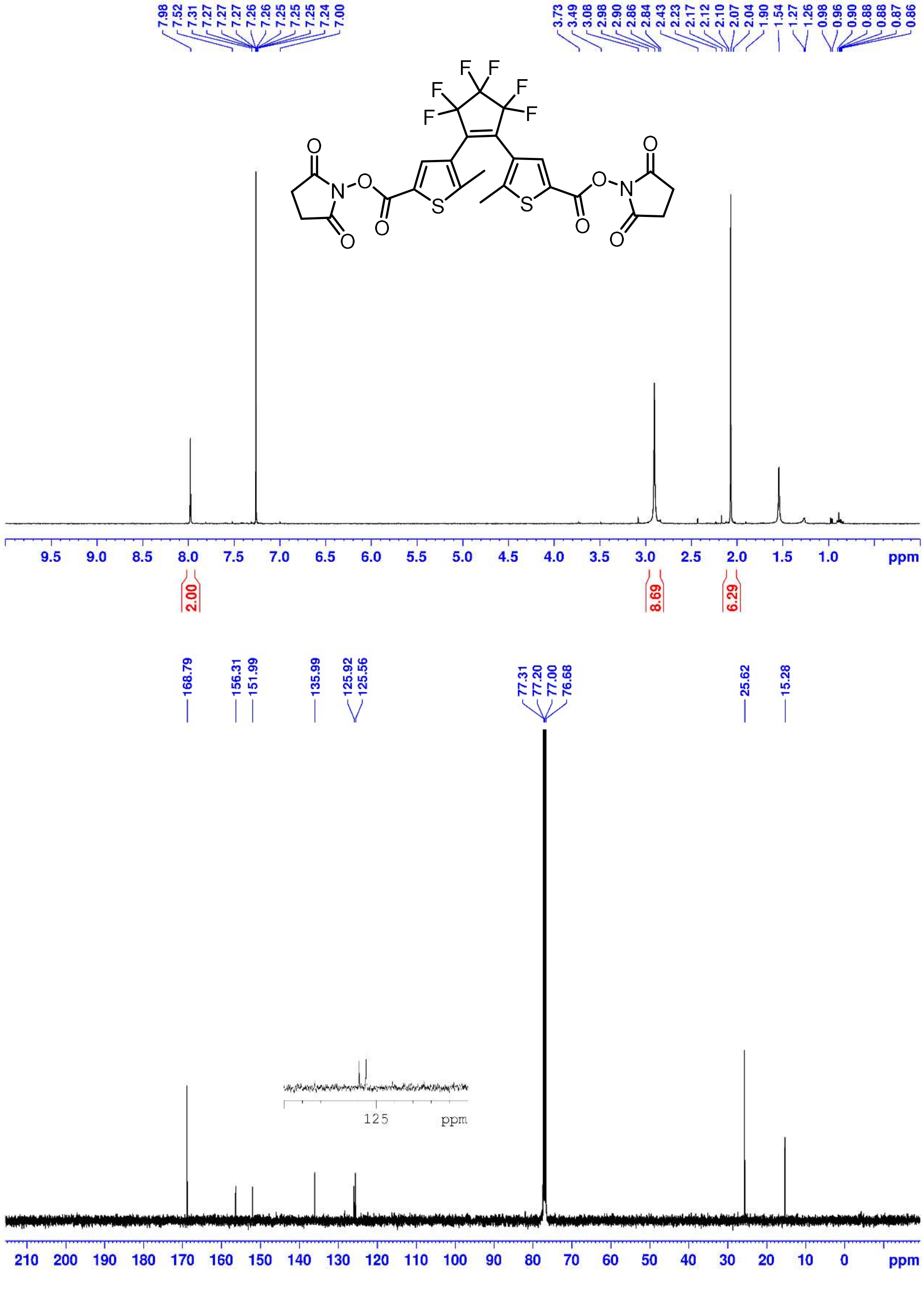

Figure S6. ^{1}H and ^{13}C NMR spectra of compound **14** in $CDCl_3$

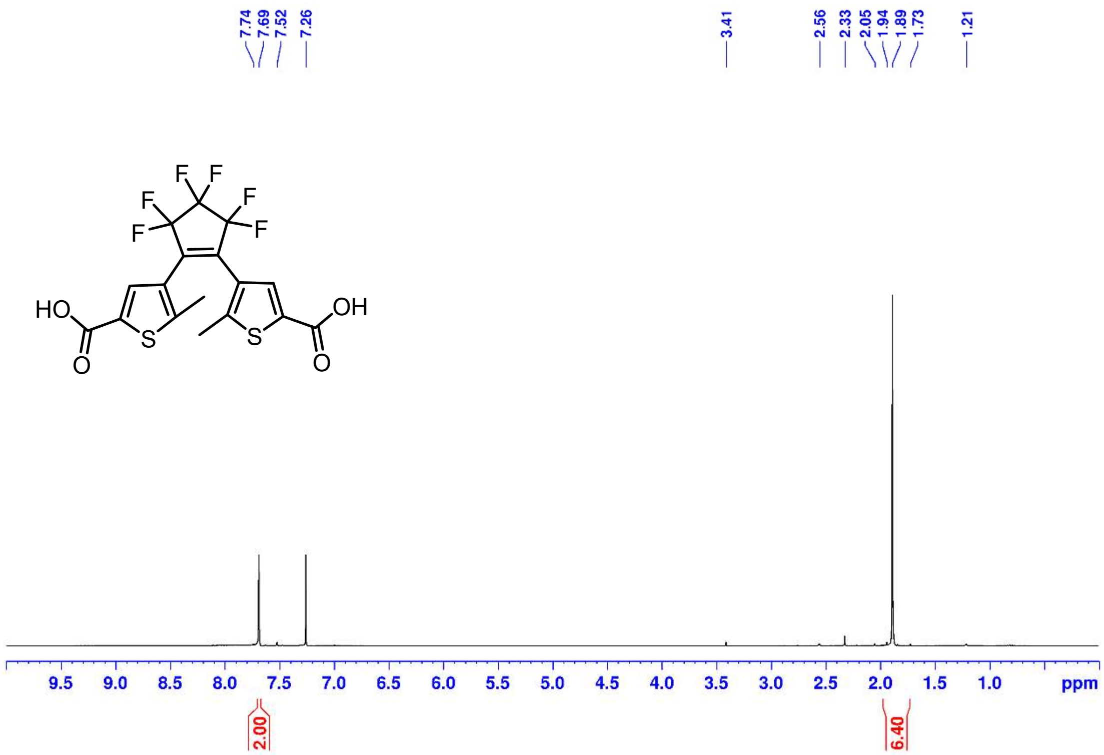

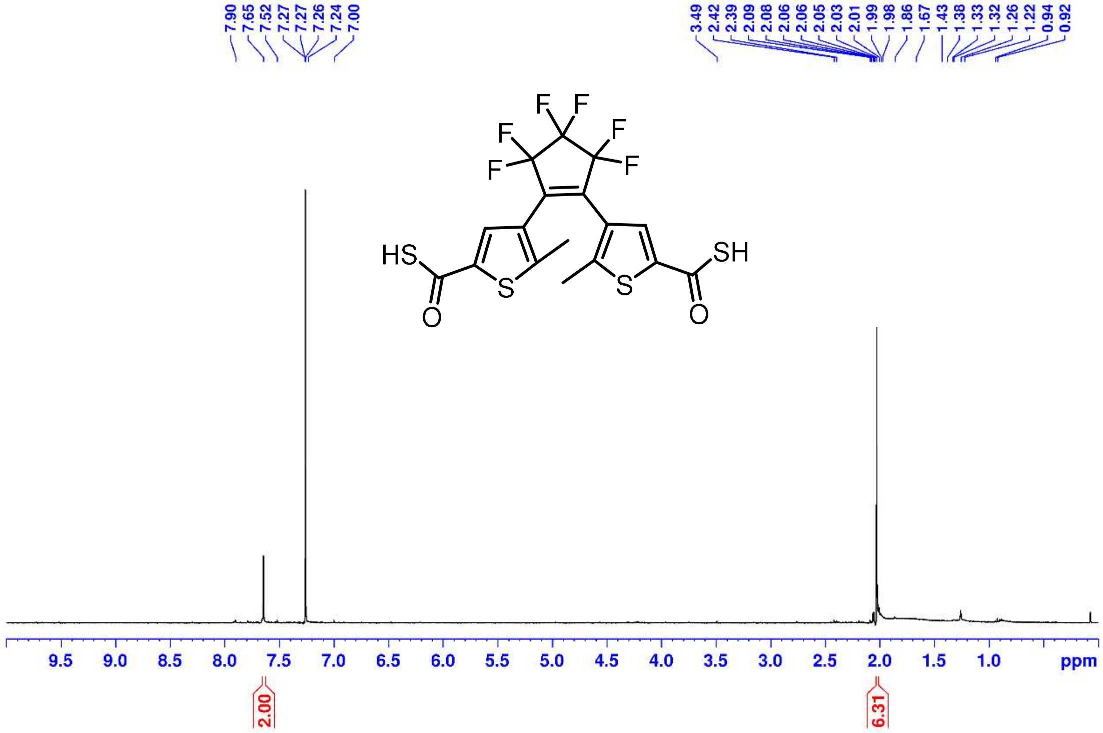

Figure S7. ^{1}H spectra of compound **2** in $CDCl_3$ with a drop of D_4-Methanol (Top) and compound **6** in $CDCl_3$

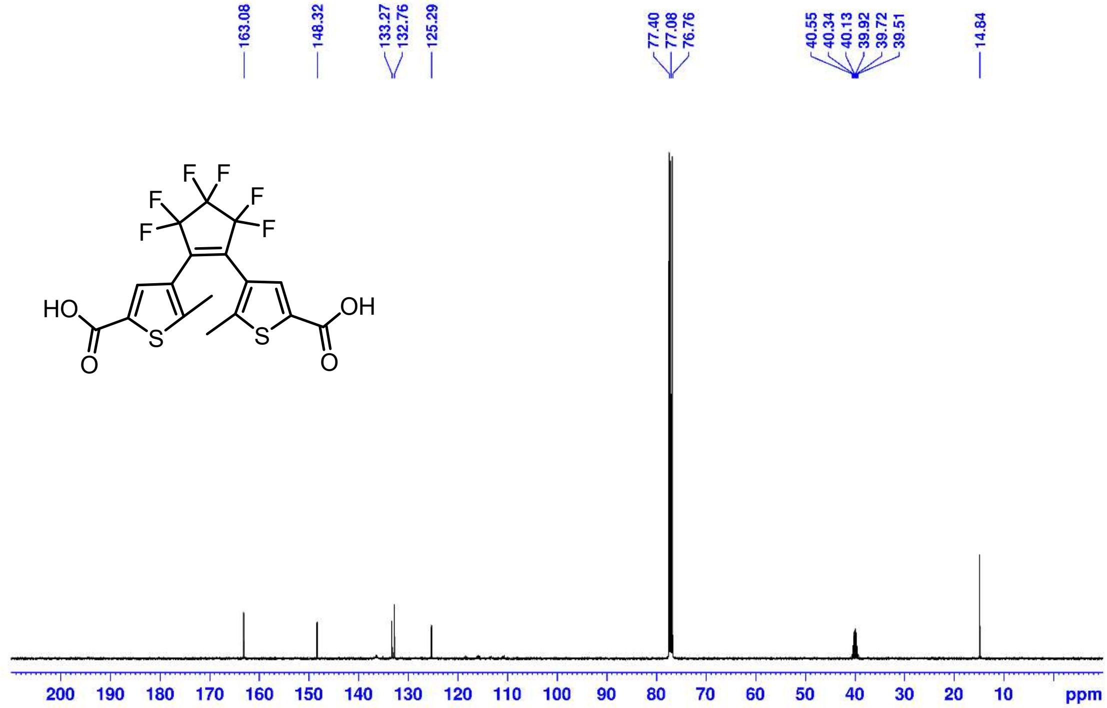

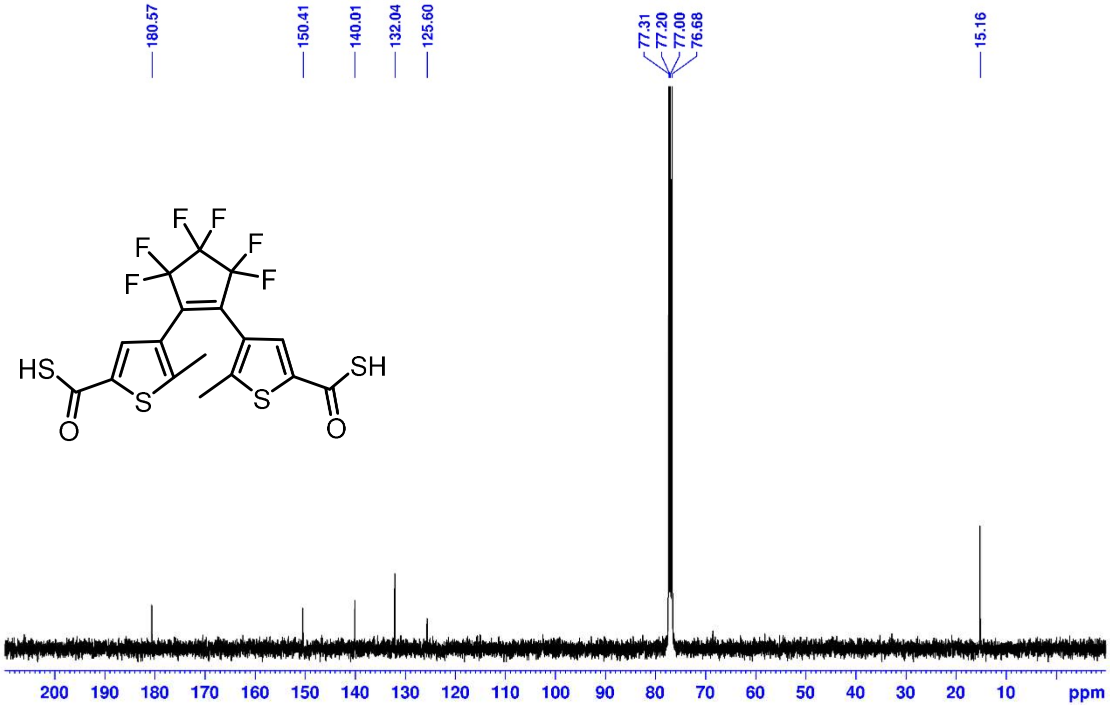

Figure S8. ^{13}C spectra of compound **2** in $CDCl_3$ with a drop of D_4-Methanol (Top) and compound **6** in $CDCl_3$

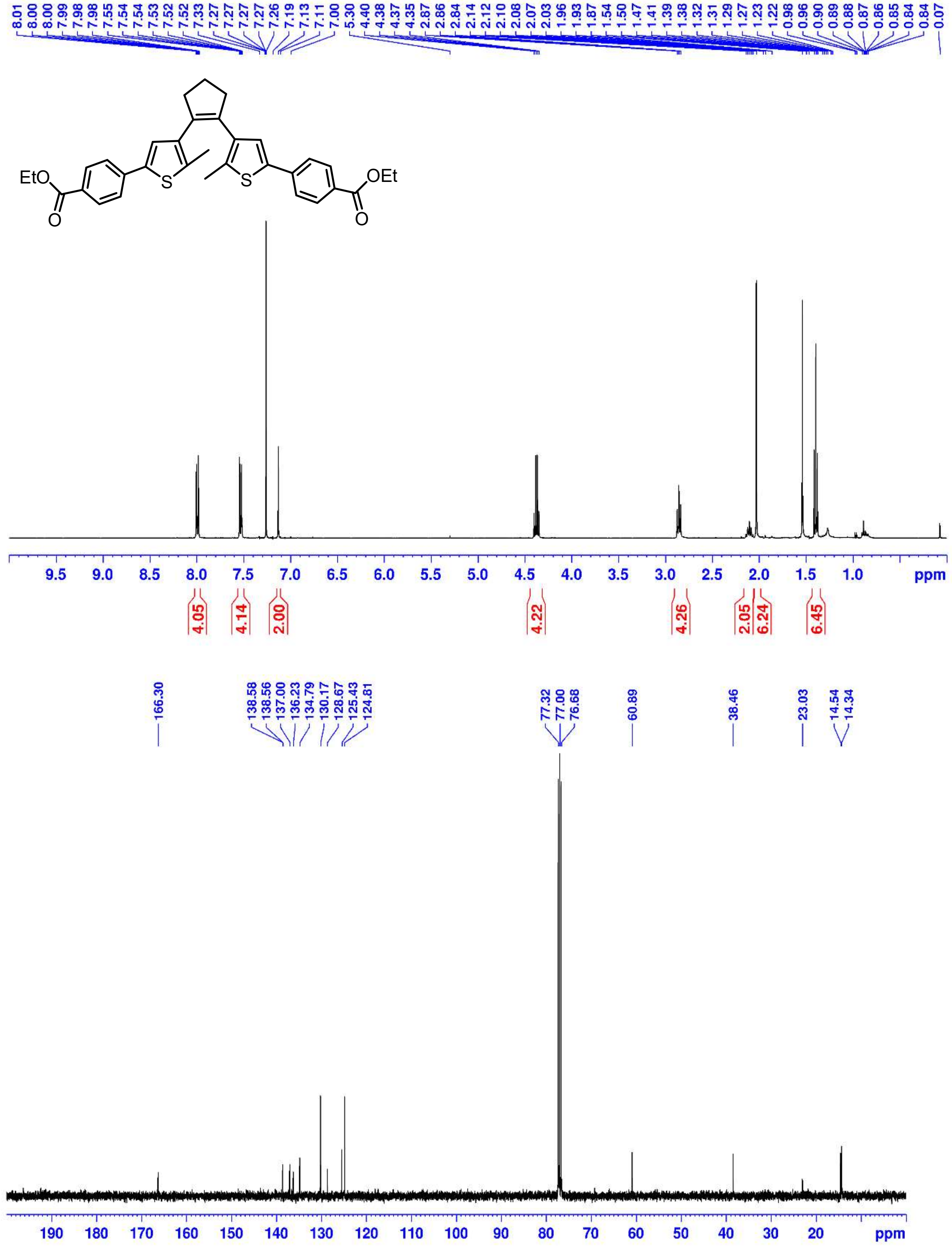

Figure S9. ^{1}H and ^{13}C NMR spectra of compound **15** in $CDCl_3$

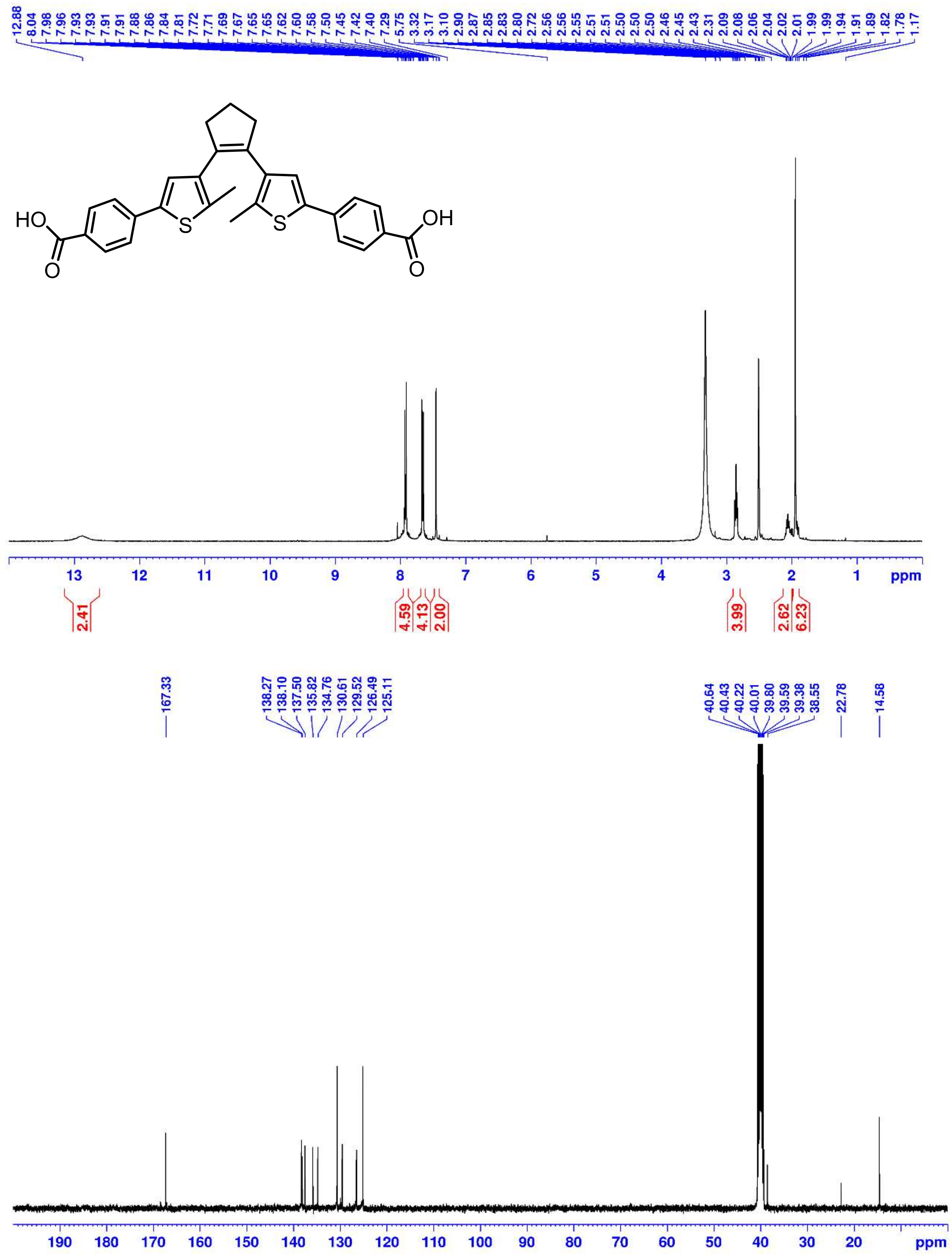

Figure S10. ^{1}H and ^{13}C NMR spectra of compound **3** in DMSO-d_6

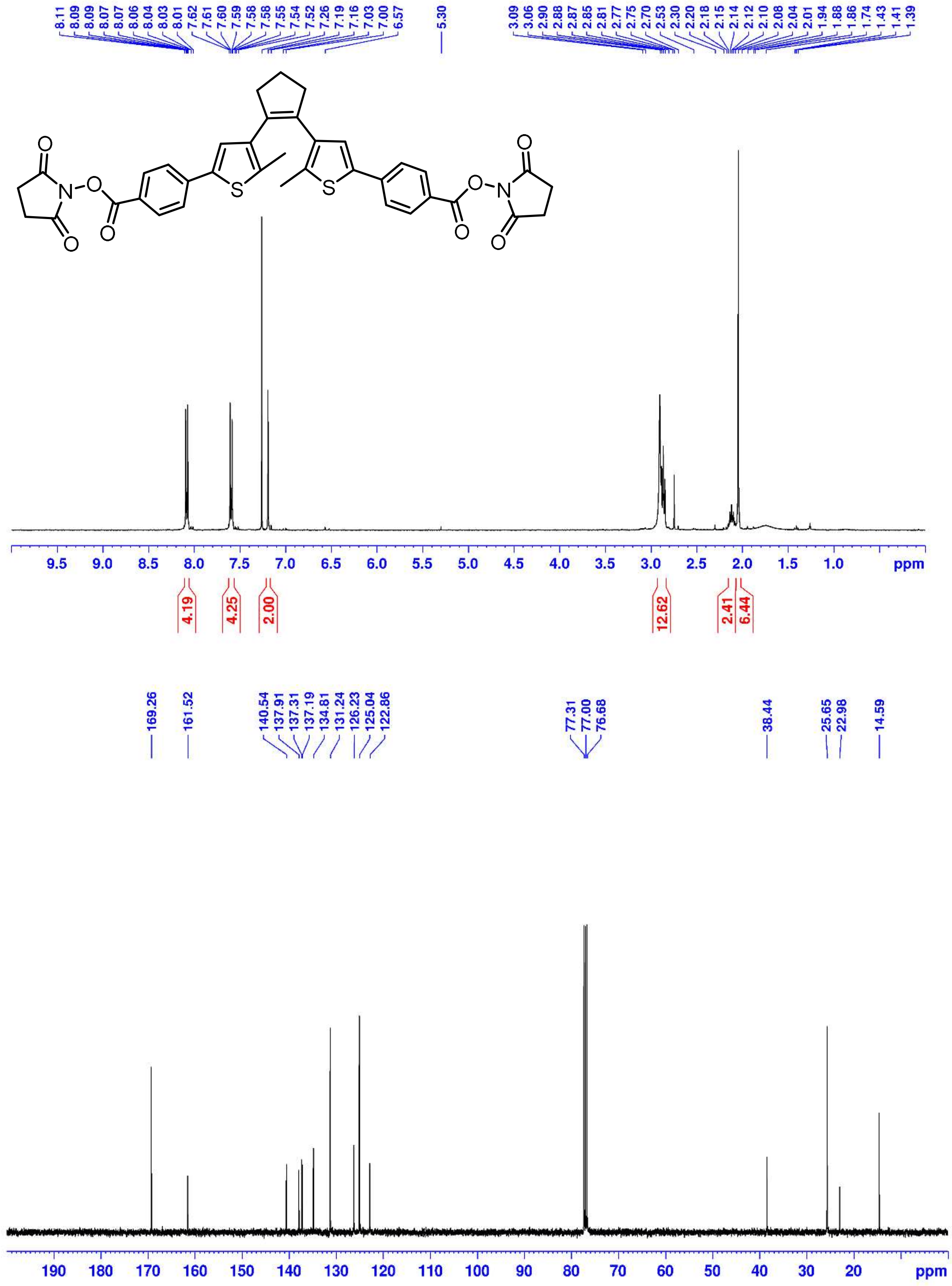

Figure S11. ^{1}H and ^{13}C NMR spectra of compound **17** in $CDCl_3$

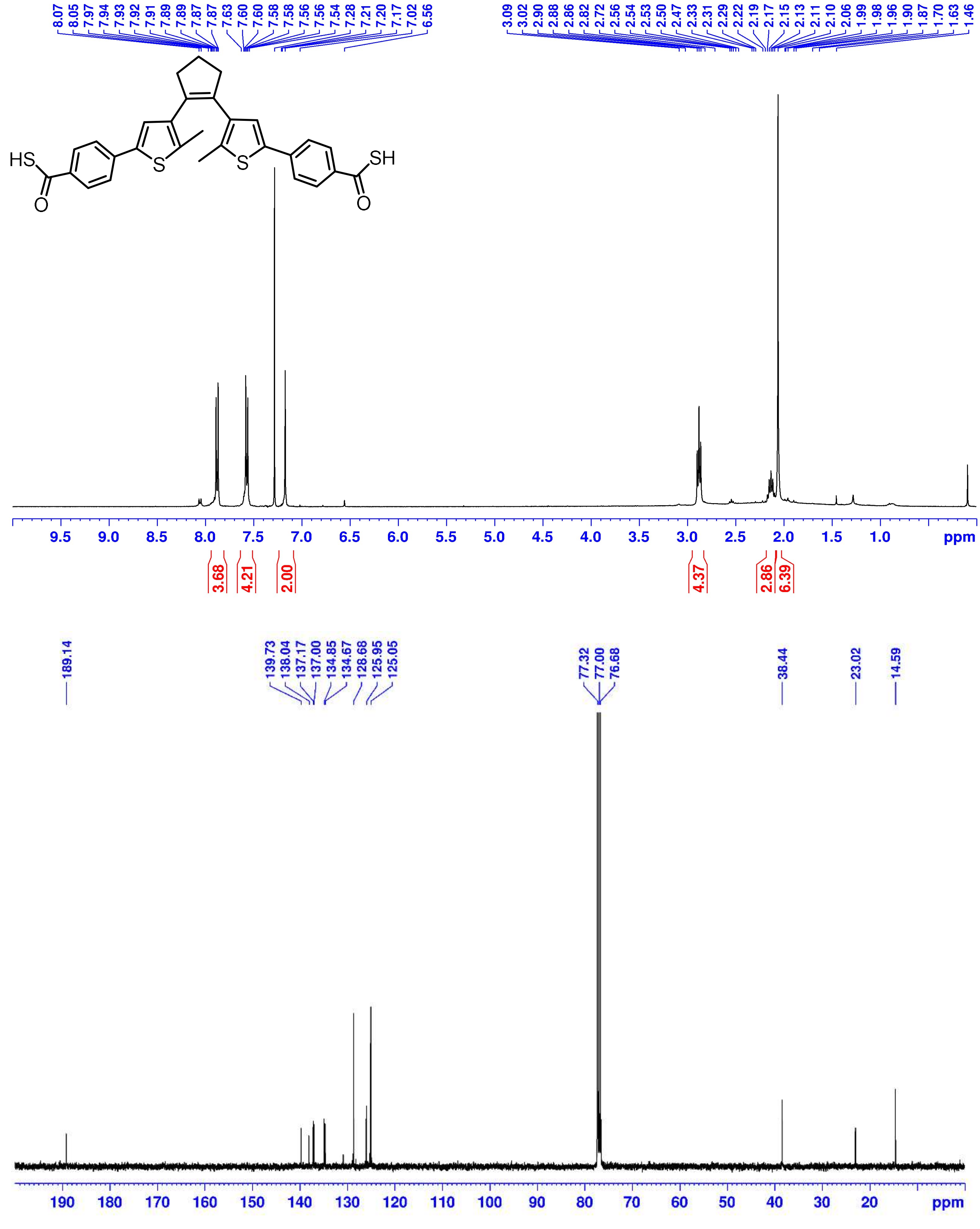

Figure S12. ^{1}H and ^{13}C NMR spectra of compound **7** in $CDCl_3$

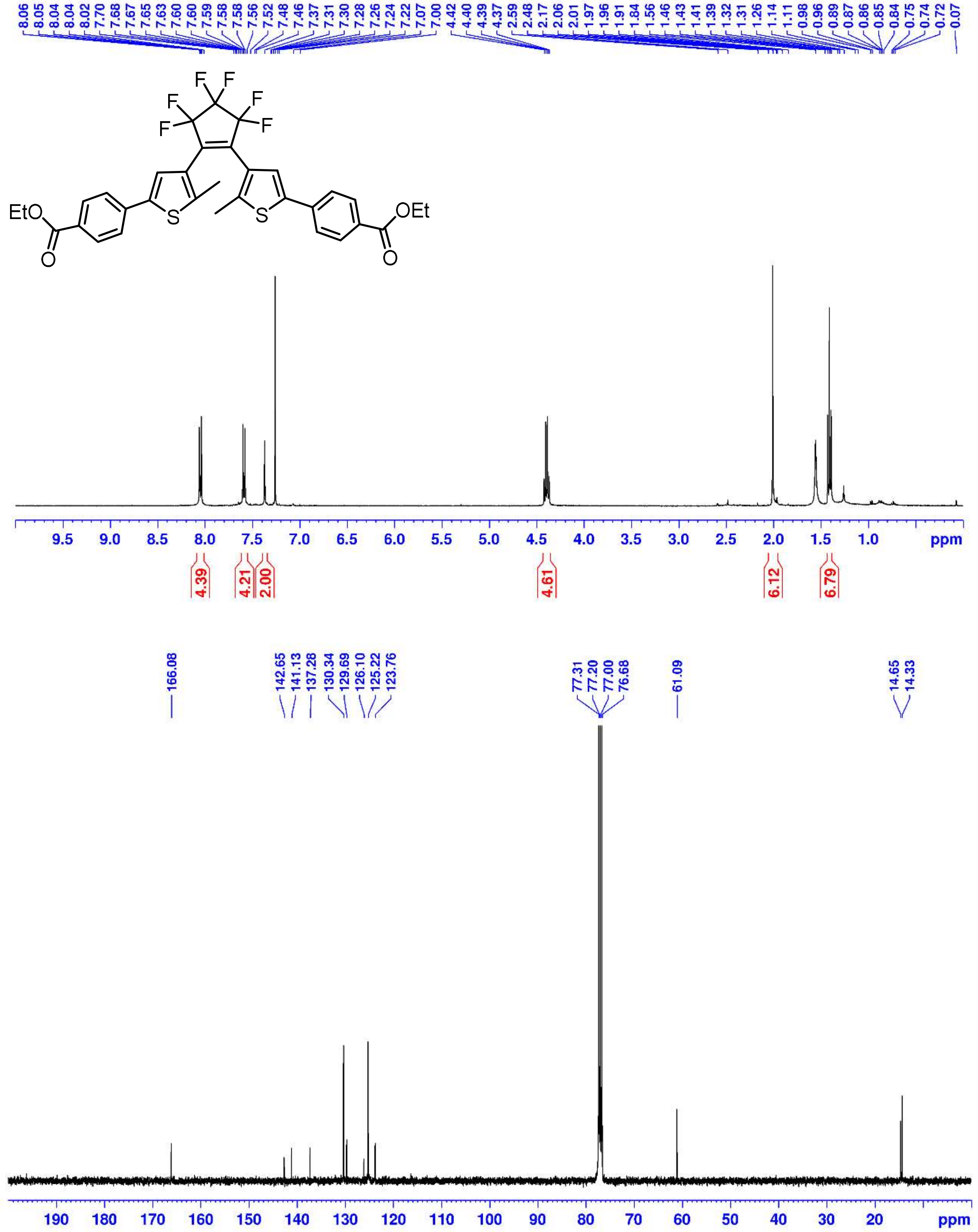

Figure S13. ^{1}H and ^{13}C NMR spectra of compound **16** in $CDCl_3$

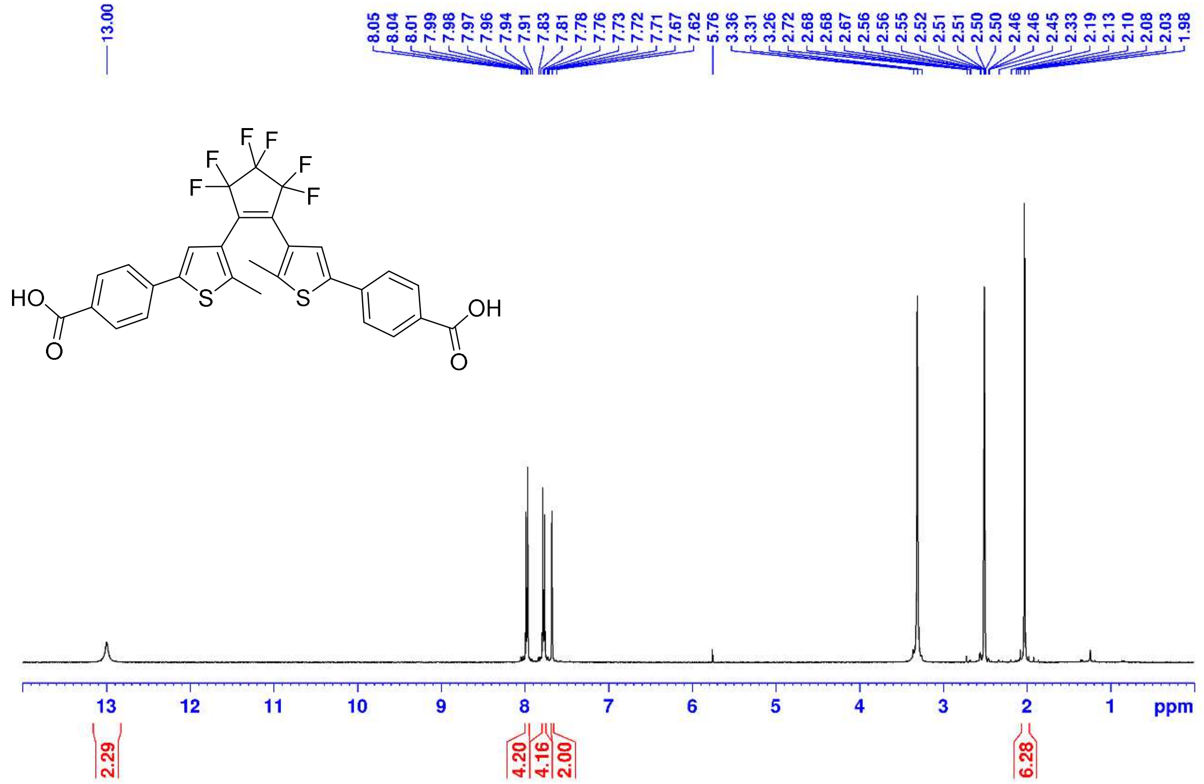

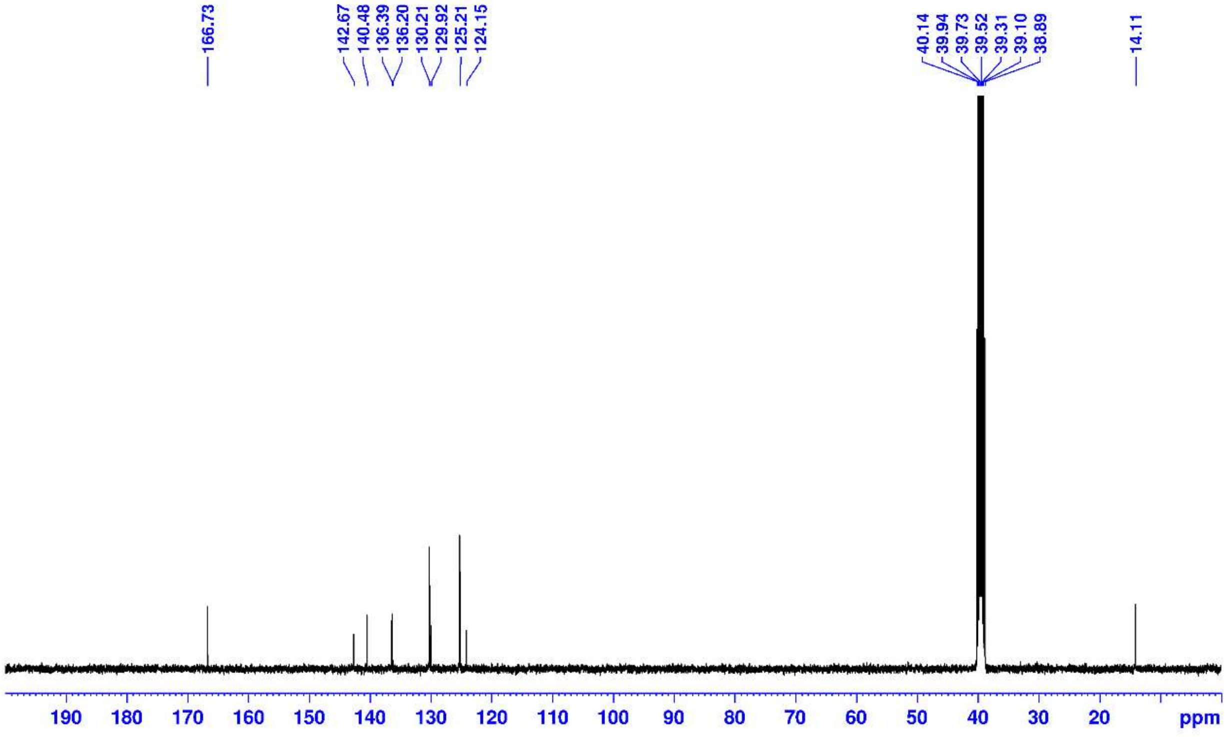

Figure S14. ^{1}H and ^{13}C NMR spectra of compound **4** in DMSO-d_6

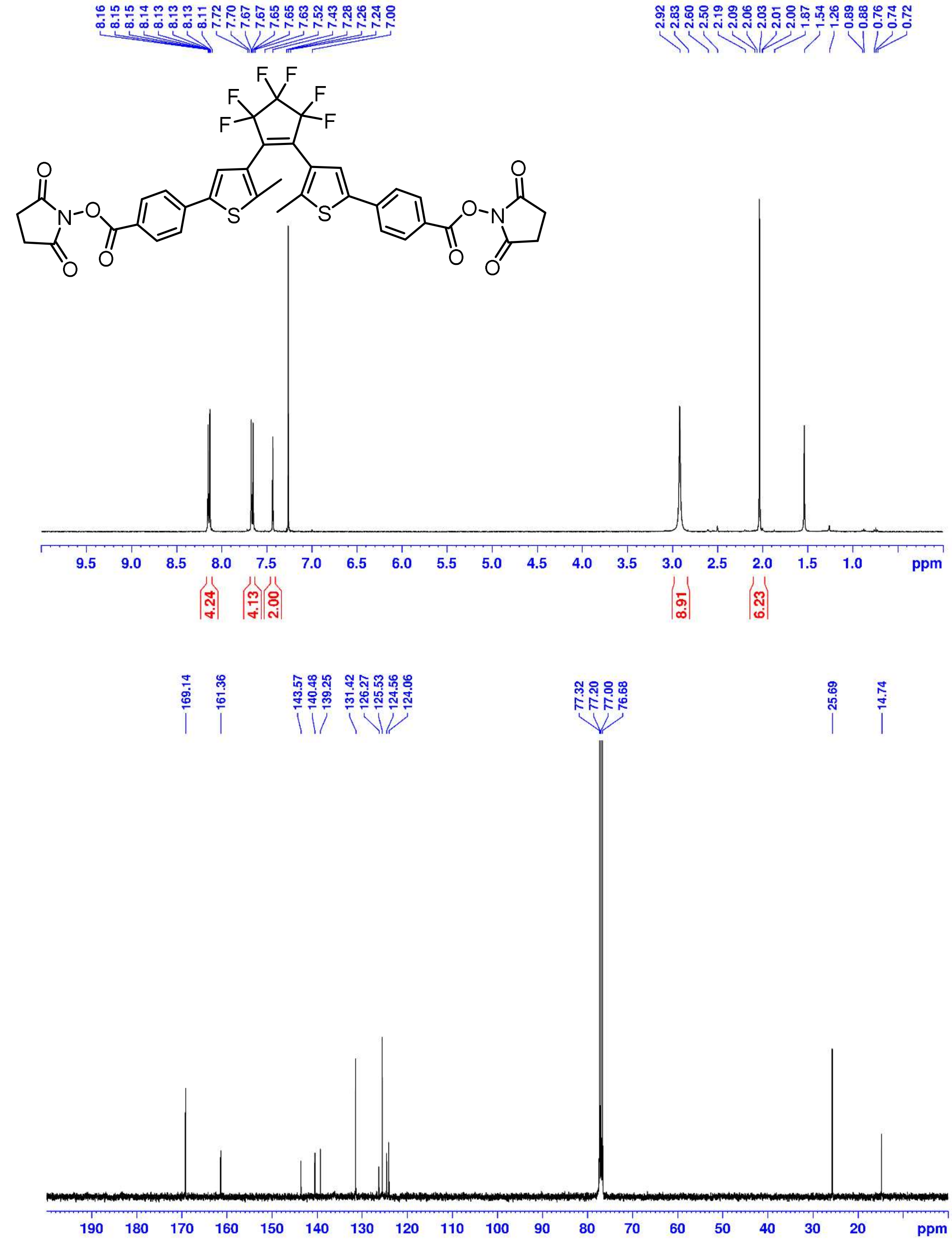

Figure S15. ^{1}H and ^{13}C NMR spectra of compound **18** in $CDCl_3$

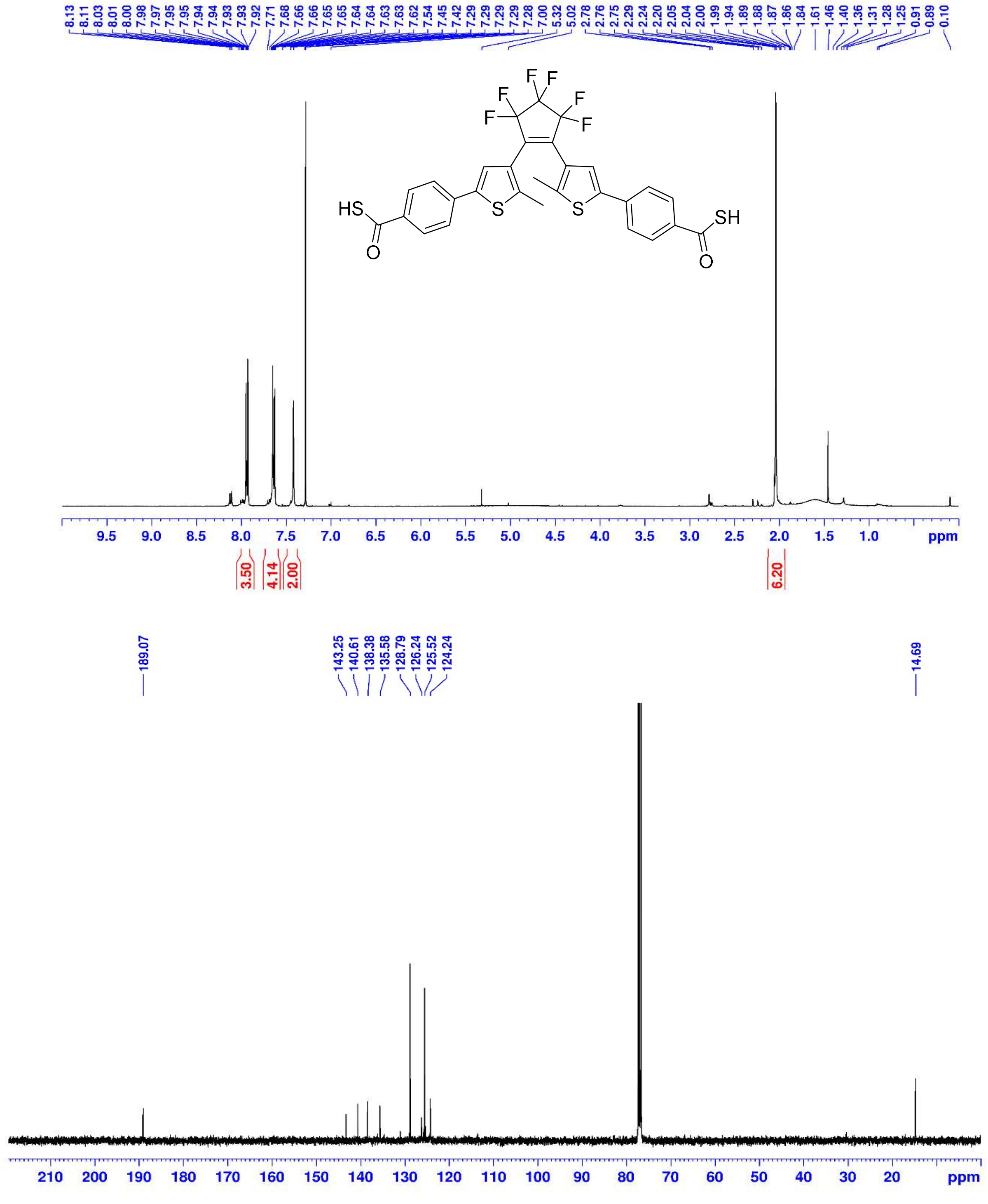

Figure S16. ^{1}H and ^{13}C NMR spectra of compound **8** in $CDCl_3$

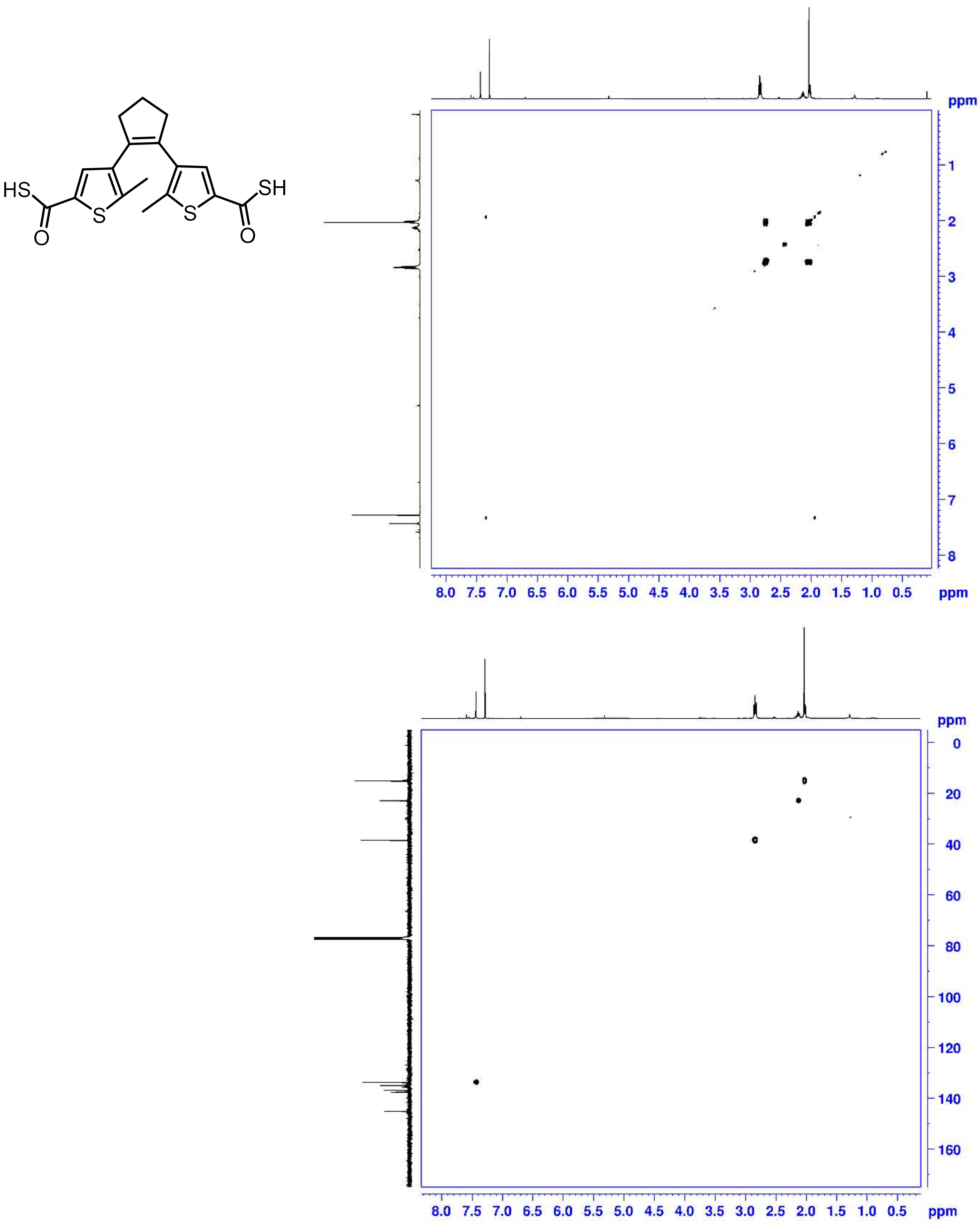

Figure S17. 2D-COSY and HSQC NMR spectra of compound **5** in $CDCl_3$

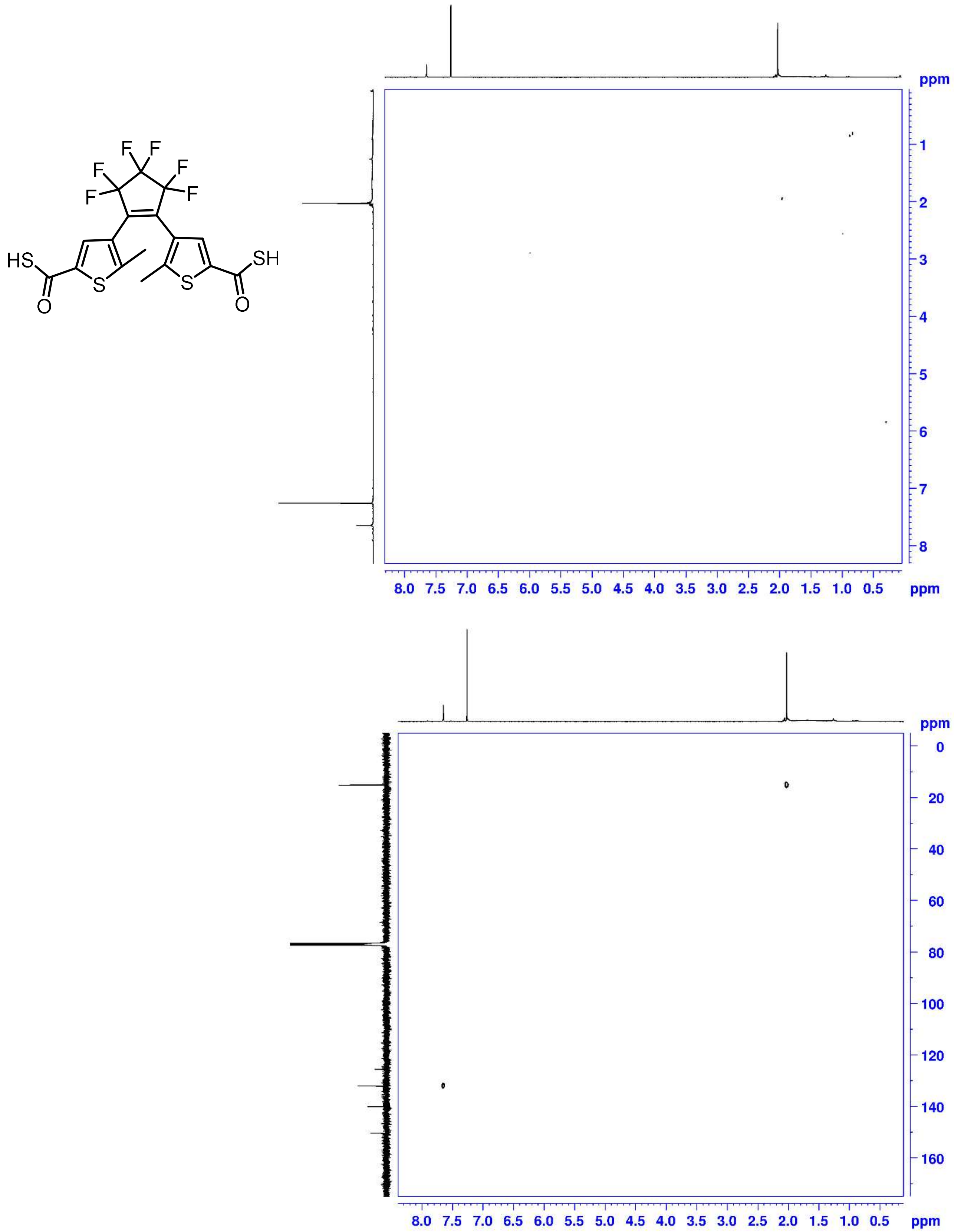

Figure S18. 2D-COSY and HSQC NMR spectra of compound **6** in $CDCl_3$

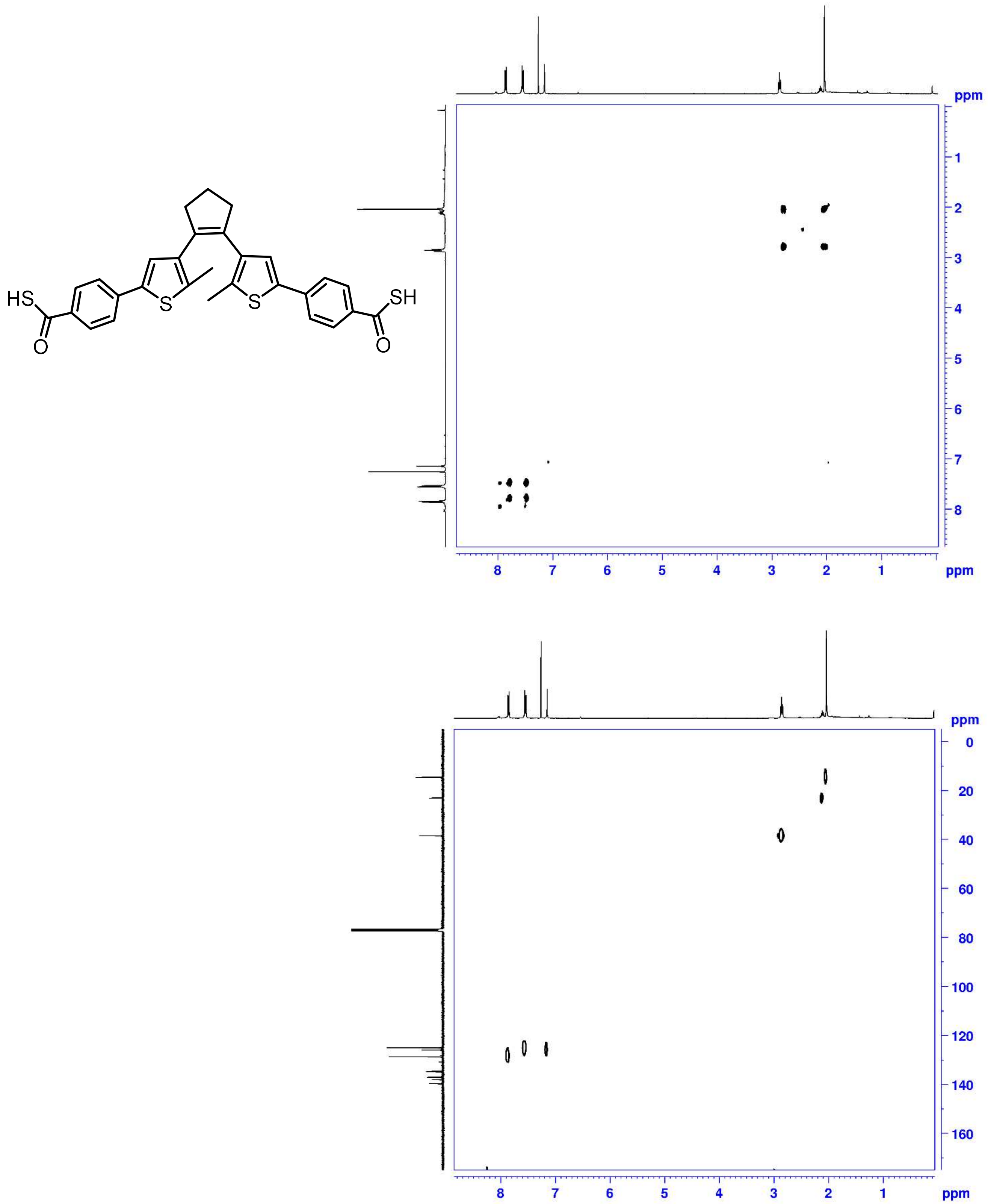

Figure S19. 2D-COSY and HSQC NMR spectra of compound **7** in $CDCl_3$

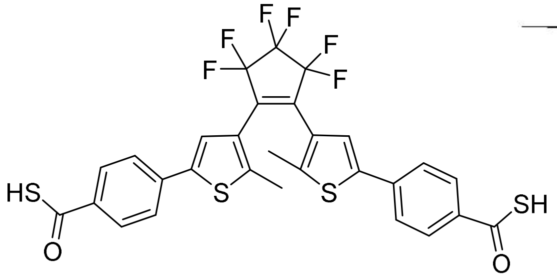

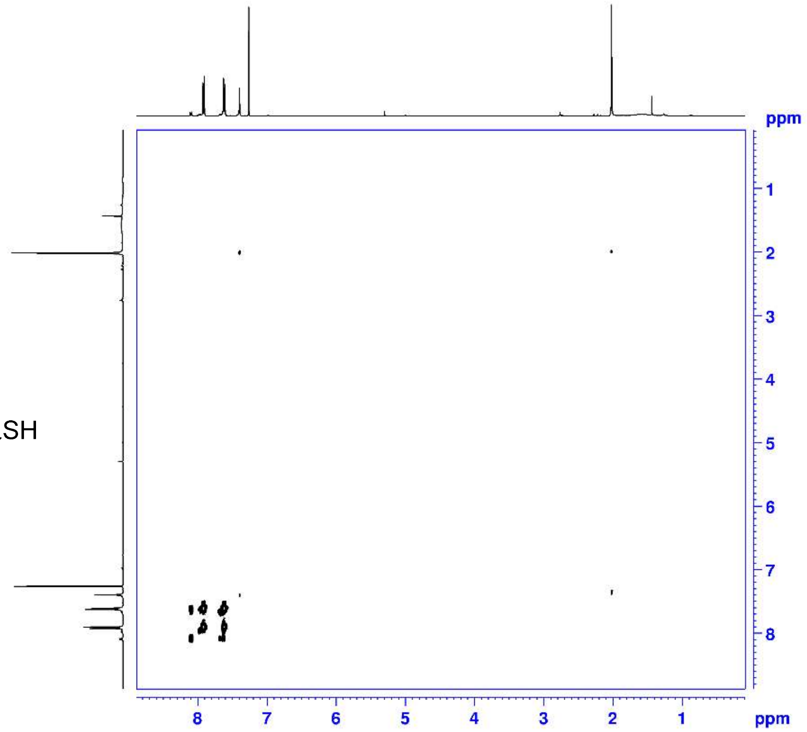

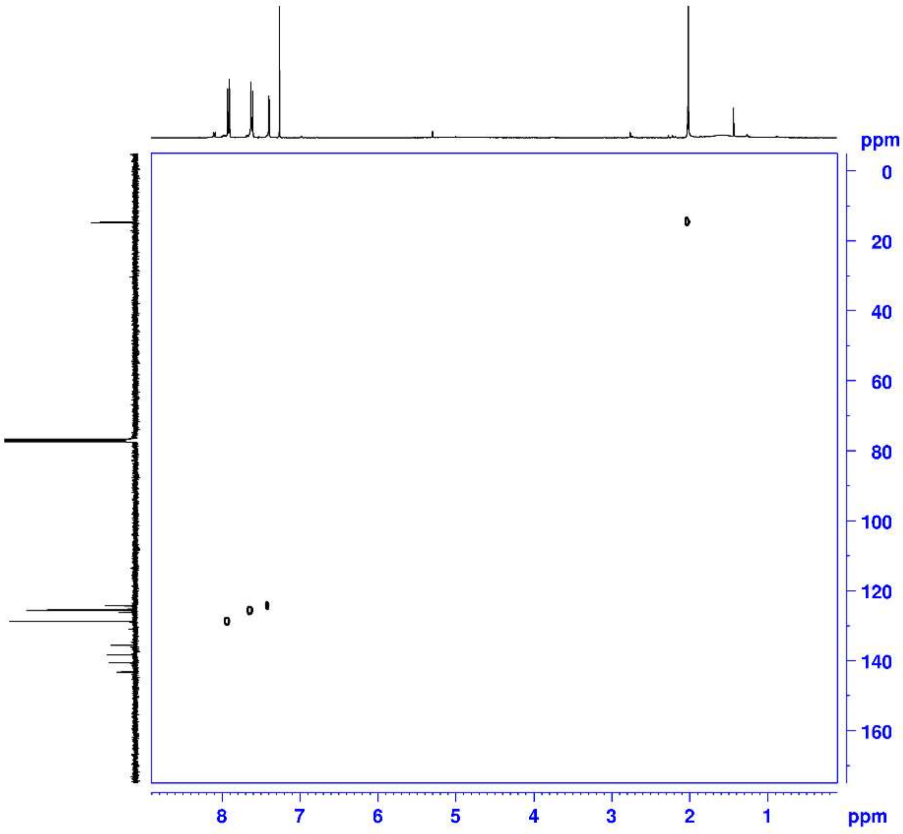

Figure S20. 2D-COSY and HSQC NMR spectra of compound **8** in $CDCl_3$

III. Additional UV-Vis absorbance spectra for thioacid compounds **5-8** under UV irradiation

The UV-Vis absorbance was recorded after exposing the solution to 302 nm at different time points for all compounds. The UV irradiation with 302 nm for all samples was done using a 6W handheld TLC lamp, model UVM-16 EL Series, with UV 302 nm and white light, which are referred as 6W UV light or white light. The procedures are the same for all compounds.

1. UV-Vis spectra for compound **5** in methanol upon irradiation at 302 nm

Compound **5** (1 mg) was added to a 2 mL volumetric flask and then methanol was added to make the solution at 1.31 mM concentration. This solution was diluted to make a 2.0 mL solution at 0.131 mM concentration for the UV experiment.

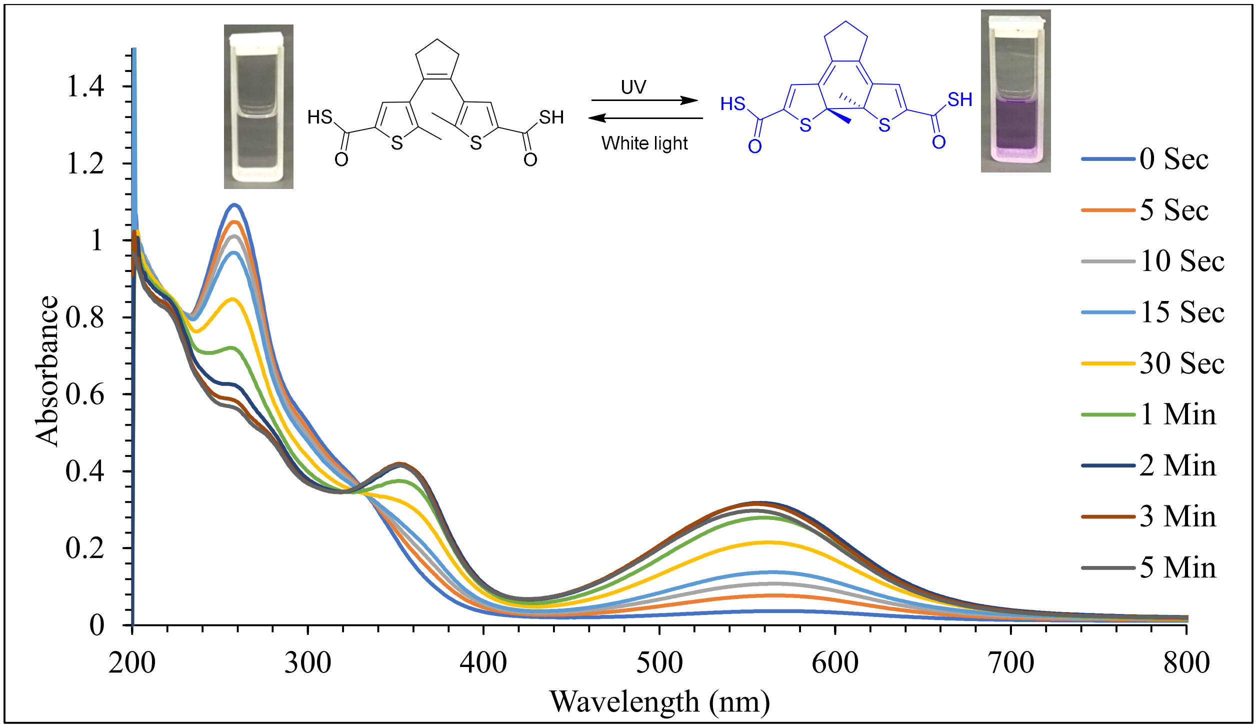

Figure S21. UV-Vis spectra of compound **5** under UV irradiation (302 nm) at different times, the concentration was 0.13 mM in methanol.

2. UV-Vis absorption spectra for compound **6** upon irradiation at 302 nm

The UV-vis absorption spectra of compound **6** after prolonged irradiation is shown in Figure S22.

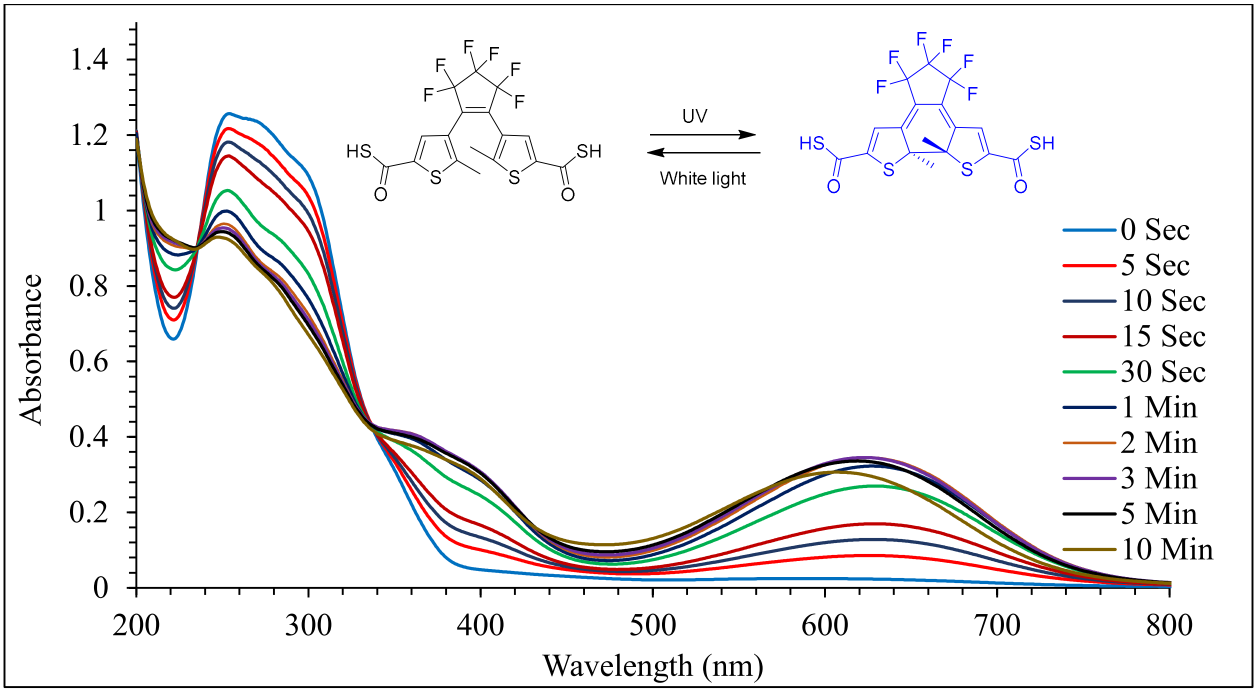

Figure S22. UV-Vis absorption spectra of 0.10 mM compound **6** in acetonitrile. Compound **6** (1.0 mg) was dissolved in 2 mL acetonitrile to prepare 1.02 mM solution in a volumetric flask. Then 0.2 mL solution was diluted to 2 mL to make 0.10 mM solution.

Compound **6** (1.6 mg) was dissolved in 4 mL acetonitrile using volumetric flask to prepare 0.82 mM solution. Then 0.2 mL solution was diluted to 2 mL to make 0.082 mM solution. UV absorbance was recorded after exposing the solution to 302 nm at different time.

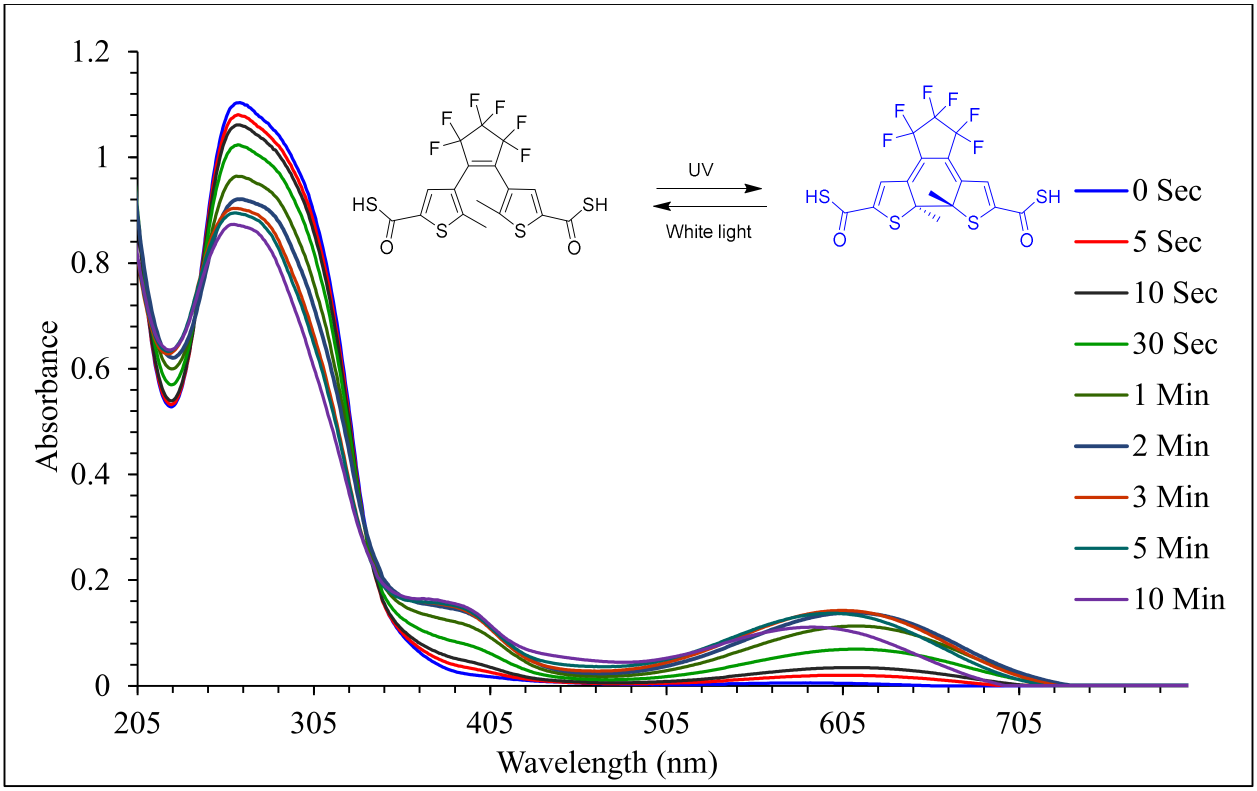

Figure S23. UV absorption spectra of compound **6** in acetonitrile, 0.082 mM.

3. UV-Vis absorption spectra of compound **7** under irradiation at 302 nm

Compound **7** (1.1 mg) was dissolved in 2 mL acetonitrile to prepare 1.03 mM solution in a volumetric flask. Then 0.2 mL solution was diluted to 2 mL acetonitrile solution to make 0.10 mM solution.

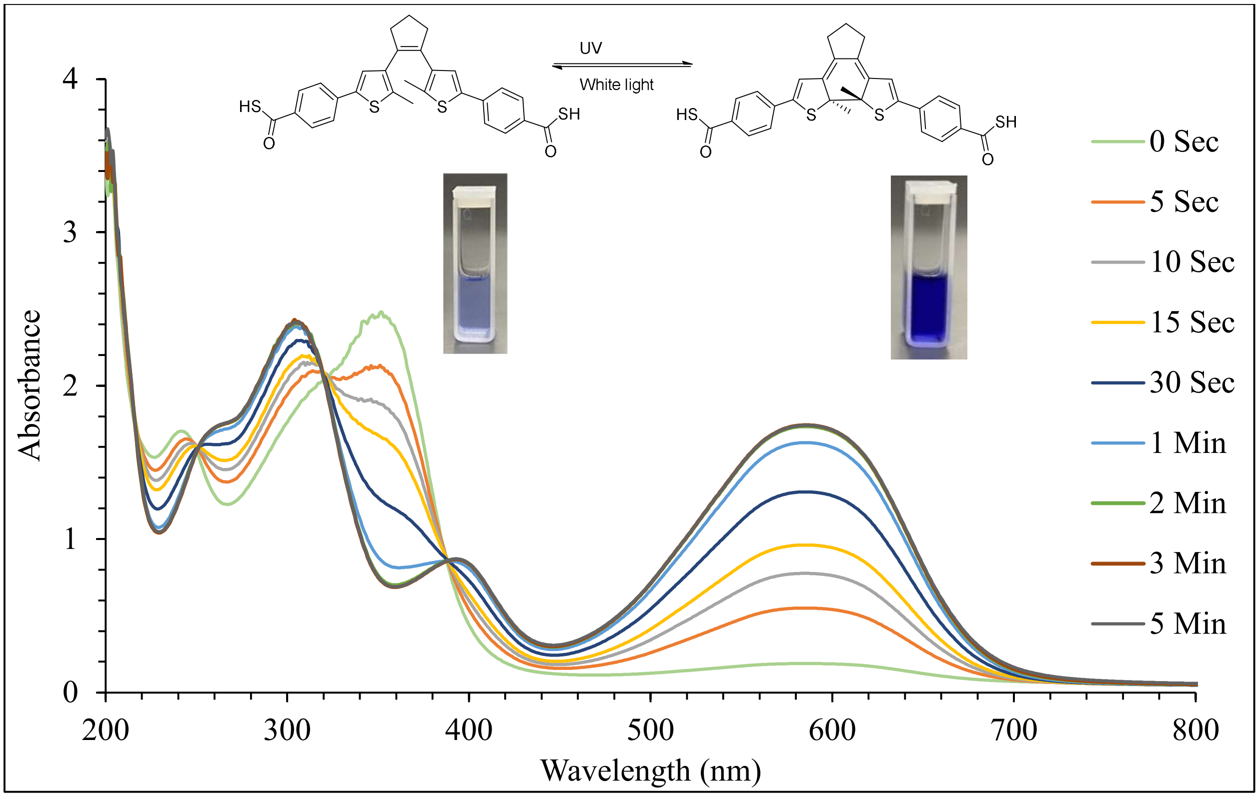

Figure S24. UV-Vis absorption spectra of compound **7** in acetonitrile, 0.10 mM.

4. UV-Vis absorption spectra of compound **8** under irradiation at 302 nm

Compound **8** (1.3 mg) was dissolved in 2.0 mL acetonitrile to make a 1.01 mM solution, then 0.1 mL solution was diluted to 1 mL acetonitrile solution to make a 0.10 mM solution.

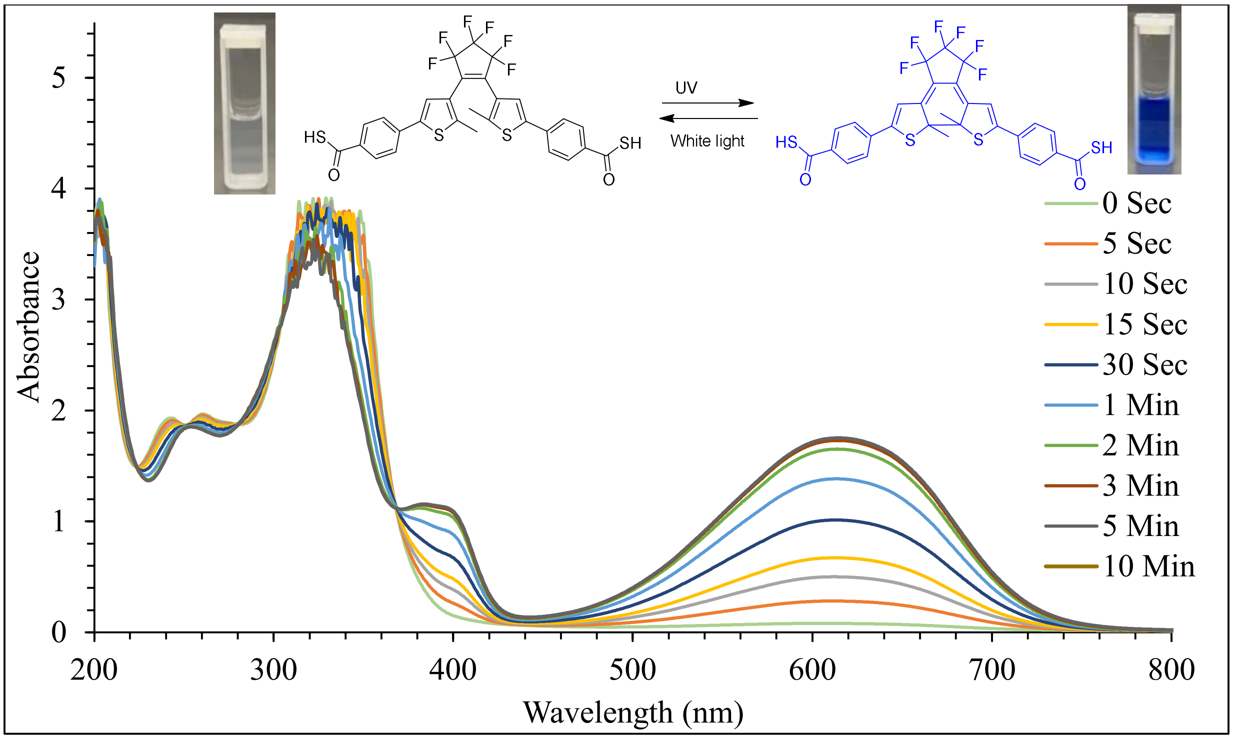

Figure S25. UV-Vis absorption spectra of compound **8** in acetonitrile, at 0.10 mM concentration.

IV. Fatigue resistance studies for compounds **5** and **6**

1. Photoswitching reversibility of compound **5**

The UV irradiation with 302 nm for all samples was done using a 6W handheld TLC lamp, model UVM-16 EL Series, with UV 302 nm and white light, which are referred as 6W UV light or white light. For the reverse reaction of cyclized to open form, the white light from the same lamp was used for the hydrogel switching experiments. For all other visible light irradiation experiments, a white LED spotlight lamp was used, 30W and 200000 Lumen for white light, this is referred to as 30W white LED lamp.

Compound **5** (1.4 mg) was dissolved in 2ml methanol in a volumetric flask to make 1.84 mM solution. Then 0.05 mL of this solution was diluted to 2.0 mL in a volumetric flask to make a

solution of 0.046 mM concentration. This solution was transferred to a cuvette and the UV absorbance was recorded using a UV-vis spectrophotometer. The sample was then irradiated with UV light (302 nm, 6W) for 3 minutes and the UV spectrum of the sample was taken, the same cuvette was then treated with white light (LED 30W) for 5 minutes to convert the cyclized form back to the open form, and the UV spectrum was recorded again, this is considered the first cycle. The same cycle of open and close was repeated for 11 cycles and the absorbance at 570 nm was used to calculate the fatigue resistance, these are shown in Figure S26.

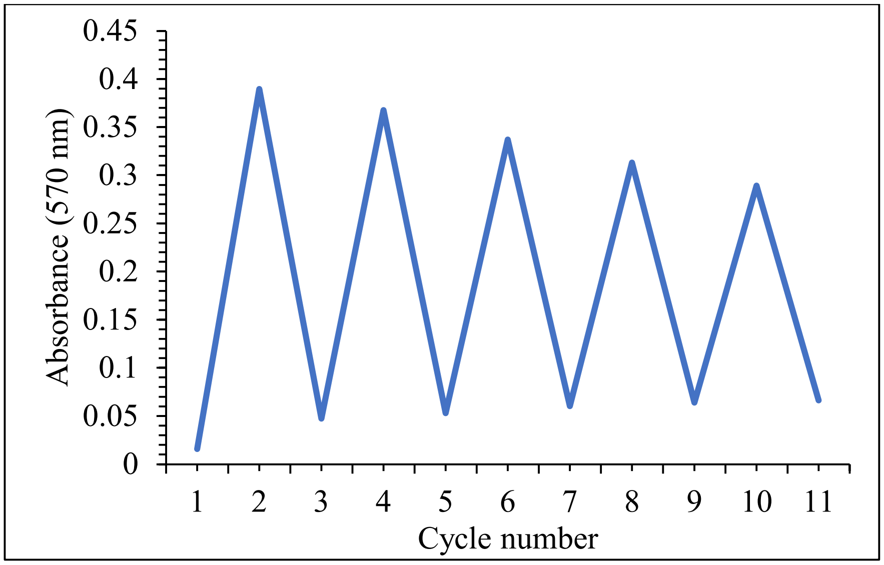

Figure S26. Photoswitching reversibility cycles of compound **5** in methanol (0.046 mM).

2. Photoswitching reversibility of compound **6**

The fatigue resistant study for compound **6** was carried out similarly and the result is shown in Figure S27. Compound **6** (1.3 mg) with 1.33 mM concentration was prepared in a 2 mL volumetric flask with acetonitrile. Then the solution was serial diluted to obtain a 0.066 mM solution. The sample was then irradiated with UV light (302 nm, 6W) for 3 minutes and the UV spectrum of the sample was taken, the same cuvette was then treated with white light (LED, 30W) for 5 minutes

to convert the cyclized form back to the open form. This cycle of opening and closing was repeated for 11 cycles and the absorbance at 630 nm was used to calculate the fatigue resistance.

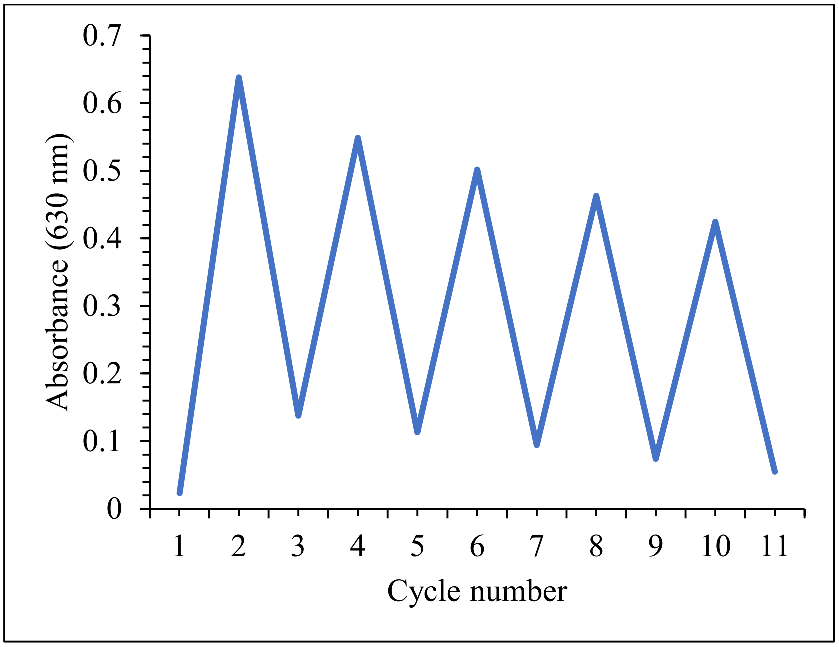

Figure S27. Photoswitching reversibility cycles of compound **6** in acetonitrile (0.066 mM)

3. Thermal stability of cyclized forms of compounds **5** and **6**

Compound **5** (1.4 mg) was dissolved in 2 mL methanol to prepare 1.84 mM solution in a volumetric flask. Then, 0.1 mL solution was diluted to 2 mL to make 0.92 mM solution. Then the solution was exposed under 302 nm UV light for 3 min and the UV absorbance was measured. The spectrum is included in Figure S28, the sample was transferred to a 1-dram sealed vial and heated at 80 °C for 5 min, the vial was cooled to room temperature and the UV absorbance was recorded again and also included in Figure S28. There was almost no change in the absorptions of the two samples, indicating that the cyclized form of compound **5** is thermally stable.

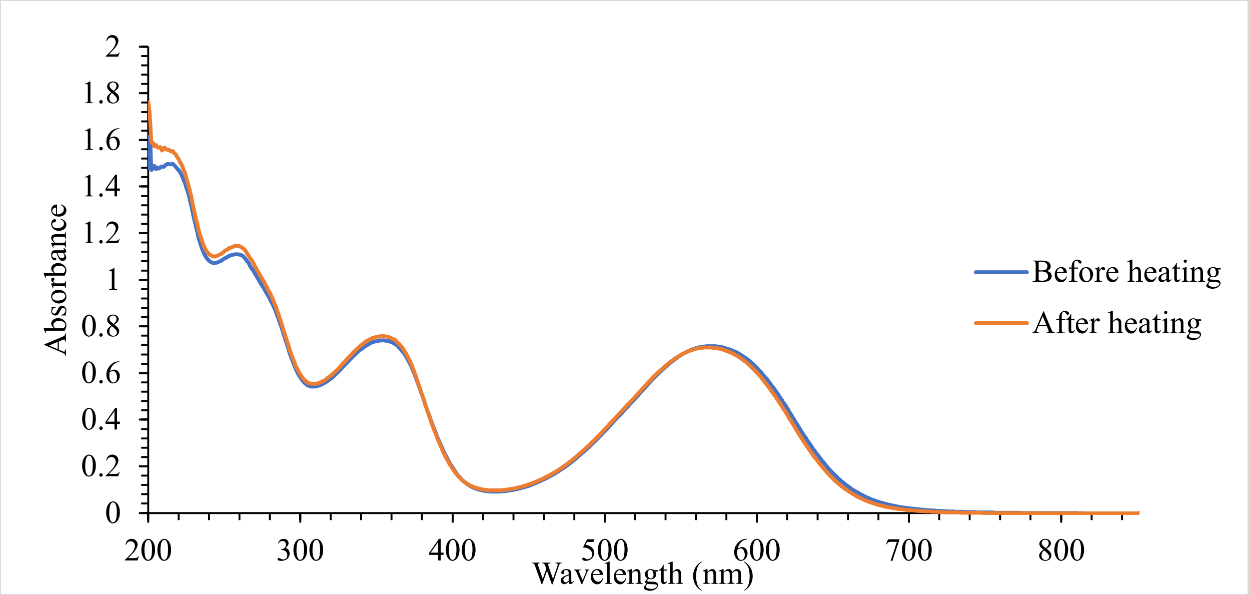

Figure S28. UV absorption spectrum of compound **5** closed form before and after heating at 80 °C for 5 minutes, the solvent is methanol at 0.92 mM concentration.

The same method was used for compound **6**, compound **6** (1.3 mg) was dissolved in 2 mL acetonitrile using a volumetric flask to prepare 1.31 mM solution. Then 0.2 mL solution was diluted to 2 mL to make 0.13 mM solution. Then the solution was exposed under 302 nm light for 3 min and UV absorbance was measured. After that the solution was heated at 80 °C for 5 min before taking UV measurement. The results are shown in Figure S29, it is interesting to note that below 400 nm, there was no significant change in the absorption signals, almost overlapping completed, however about 420 nm, the sample after heating showed significant blue shift of the λmax at 590 nm before heating shifted to 568 nm after heating, with approximately 22 nm shift.

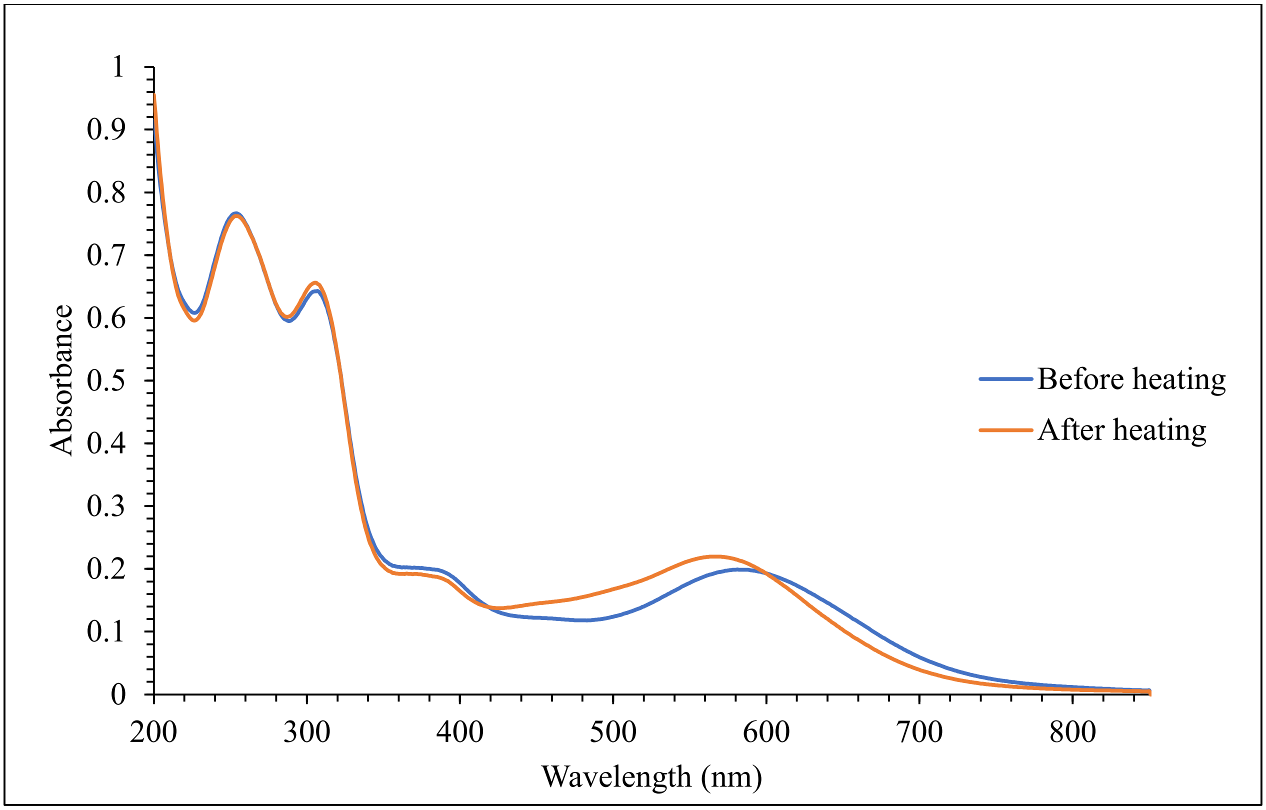

Figure S29. UV absorption spectrum of compound **6** closed form before and after heating at 80 °C for 5 minutes. The solvent is acetonitrile and 0.13 mM concentration. The peak at 590 nm redshifted to 568 nm after heating.

V. Stability of thioacids analyzed by NMR spectroscopy

1. Stability of thioacid under UV irradiation

Using compound **5** as an example, the stability of the thioacid moiety was analyzed using NMR. The NMR sample of compound **5** was prepared using 4.0 mg compound **5** in 0.5 mL $CDCl_3$. Then the NMR tube was exposed under UV light (6W, 302 nm) for 30 minutes and the ^{1}H NMR was recorded again. The NMR tube is high throughput glass tube and not UV transparent, after UV irradiation, the compound had partially cyclized to form the closed form and the NMR spectrum

exhibited two sets of signals, corresponding to the open and the closed forms. The ^{13}C NMR spectrum revealed the new of carbonyl peak at 183.4 ppm for the closed form. The distinct two sets of signals and the further downfield shifted carbonyl signal from 180.8 ppm for the open form indicated that the thioacid functional group (COSH) was preserved during the photoirradiation.

The NMR data for the open and closed forms are:

Open form: ^{1}H NMR (400 MHz, $CDCl_3$) δ 7.40 (s, 2H), 2.81 (t, J = 7.5, 4H), 2.15-2.05 (m, 2H), 2.00 (s, 6H), ^{13}C NMR (100 MHz, $CDCl_3$) δ 180.8, 145.1, 137.6, 136.8, 135.0, 133.7, 38.4, 22.8, 15.0.

Closed form: ^{1}H NMR (400 MHz, $CDCl_3$) δ 6.67 (s, 2H), 2.49 (t, J = 7.5, 4H), 1.98-1.88 (m, 2H), 2.01 (s, 6H) ^{13}C NMR (100 MHz, $CDCl_3$) 183.4, 146.0, 143.3, 142.5, 126.9, 66.6, 30.1, 27.5, 24.7. (67.9, 25.6 are from THF)

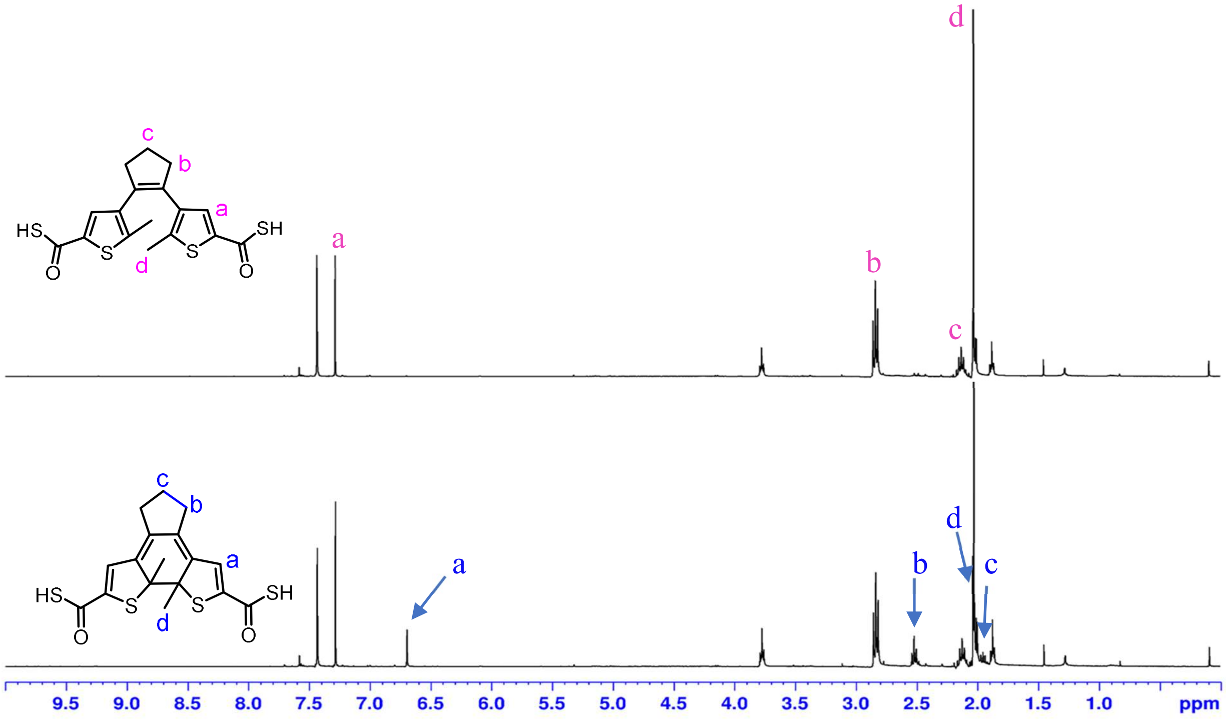

Figure S30. ^{1}H NMR ($CDCl_3$, 400 MHz) spectra, open form (top) and close form (bottom). The sample contains small amount of THF.

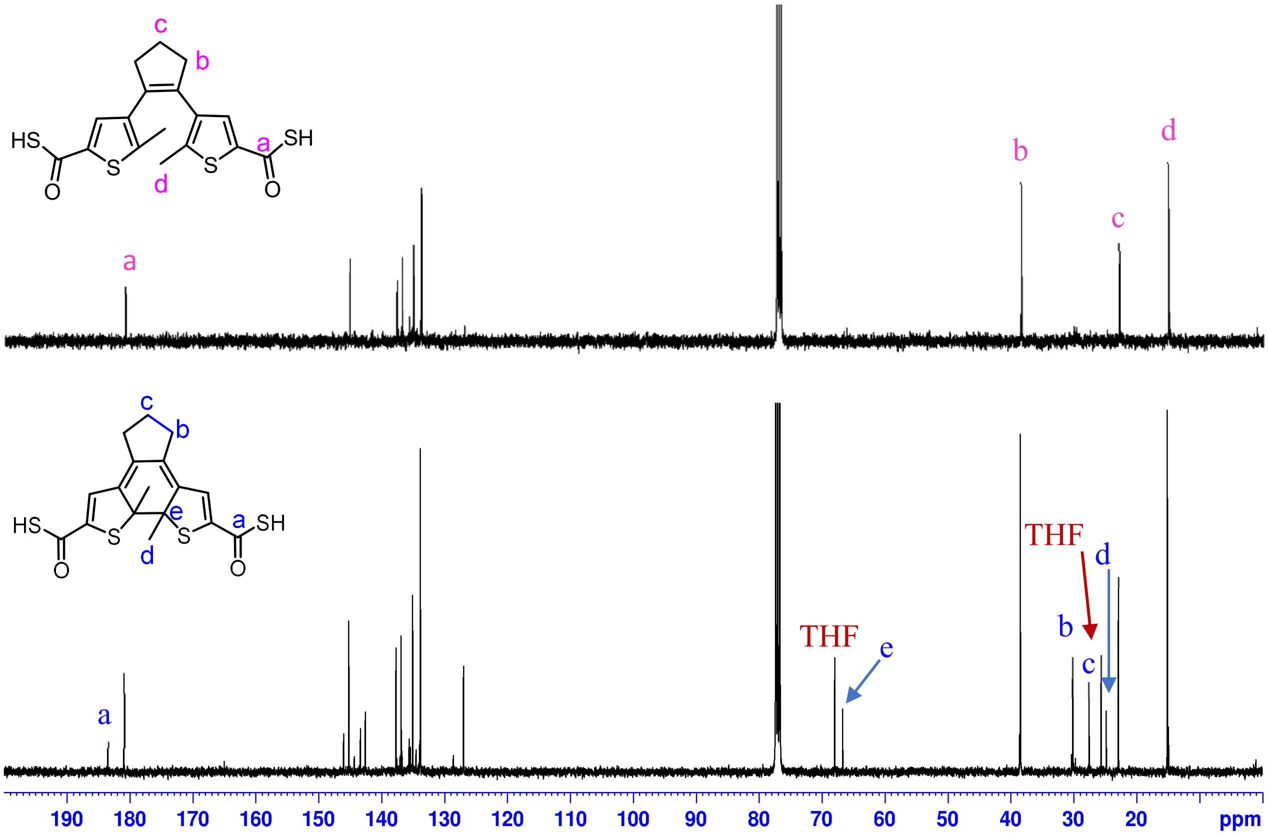

Figure S31. ^{13}C NMR ($CDCl_3$, 100 MHz) spectra of compound **5**, the top spectrum is the open form from a different sample. The bottom spectrum is corresponding to the sample in Figure S30 after UV irradiation to form partially closed form.

2. Stability of compound **5** in DMSO

Free thioacids are not stable in DMSO, they are converted to the carboxylic acid. Compound **5** (4 mg) was dissolved in 0.5 mL d_6-DMSO and after heating it changed to red color, the ^{1}H NMR was taken and observed that the thioacid acid was converted to dicarboxylic acid.

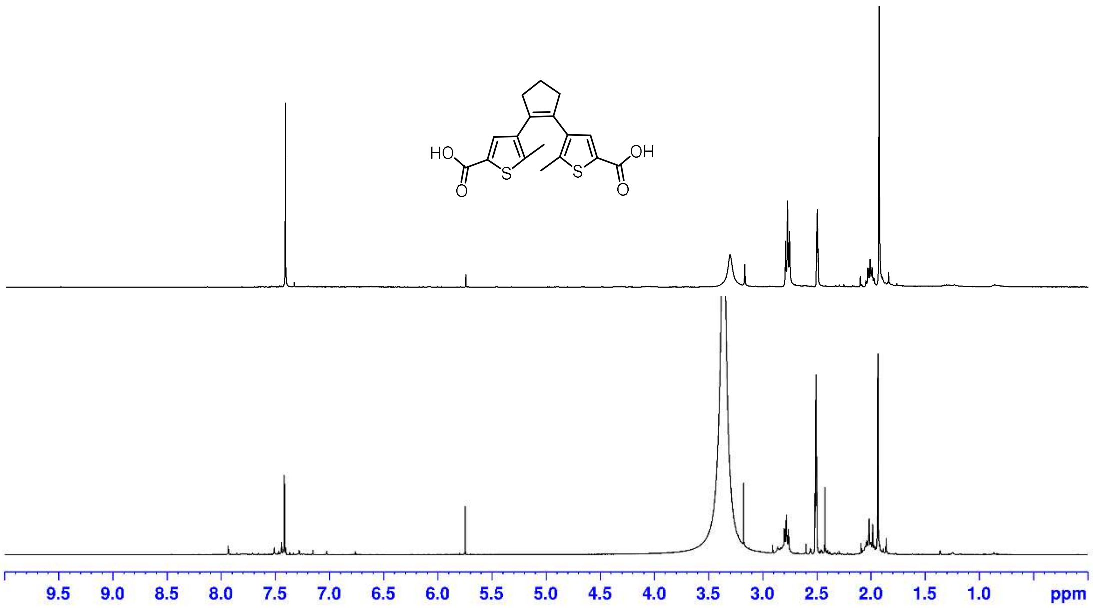

Figure S32. ^{1}H NMR spectra of compounds **1** (top) and **5** (bottom) in D_6-DMSO, 400 MHz.

3. Stability of the potassium salt of compound **5**

The anion form of the thioacid was prepared and analyzed. The results are shown in Figure S33 and S34. Compound **5** (12 mg, 0.032 mmol, 1 eq) was taken in scintillation vial dissolved in EtOH and KOH (3.6 mg, 0.064 mmol, 2 eq) was added and sonicated for 15 min. Then the solvent was removed and the residue solid is the corresponding potassium salt of compound **5**. To prepare NMR samples, 6 mg of the salt was dissolved in D_6-DMSO and ^{1}H NMR and ^{13}C NMR was measured. The same sample was also checked one week later and the NMR spectra were recorded. As shown in Figures S33 and S34, the thioacid salt is stable and didn't change in one week.

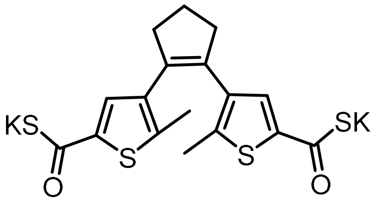

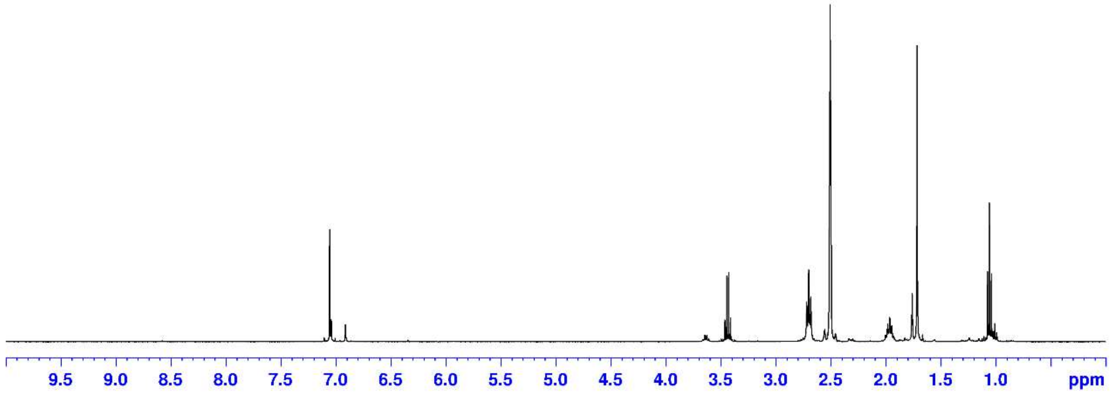

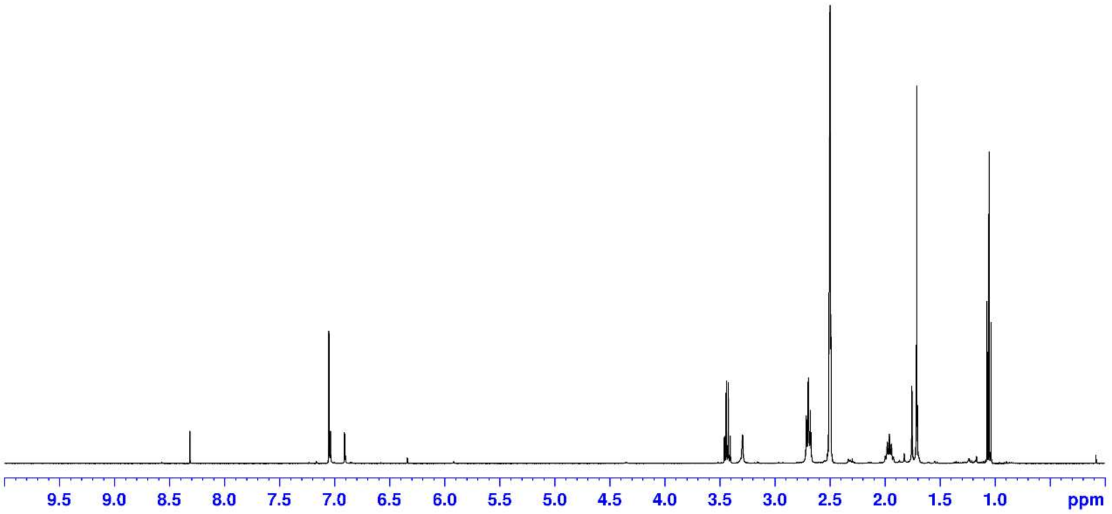

Figure S33. The ^{1}H NMR (D_6-DMSO, 400 MHz) of potassium salt of compound **5**, top is freshly prepared sample, the bottom spectrum is obtained after one week. The sample contains a small amount of ethanol.

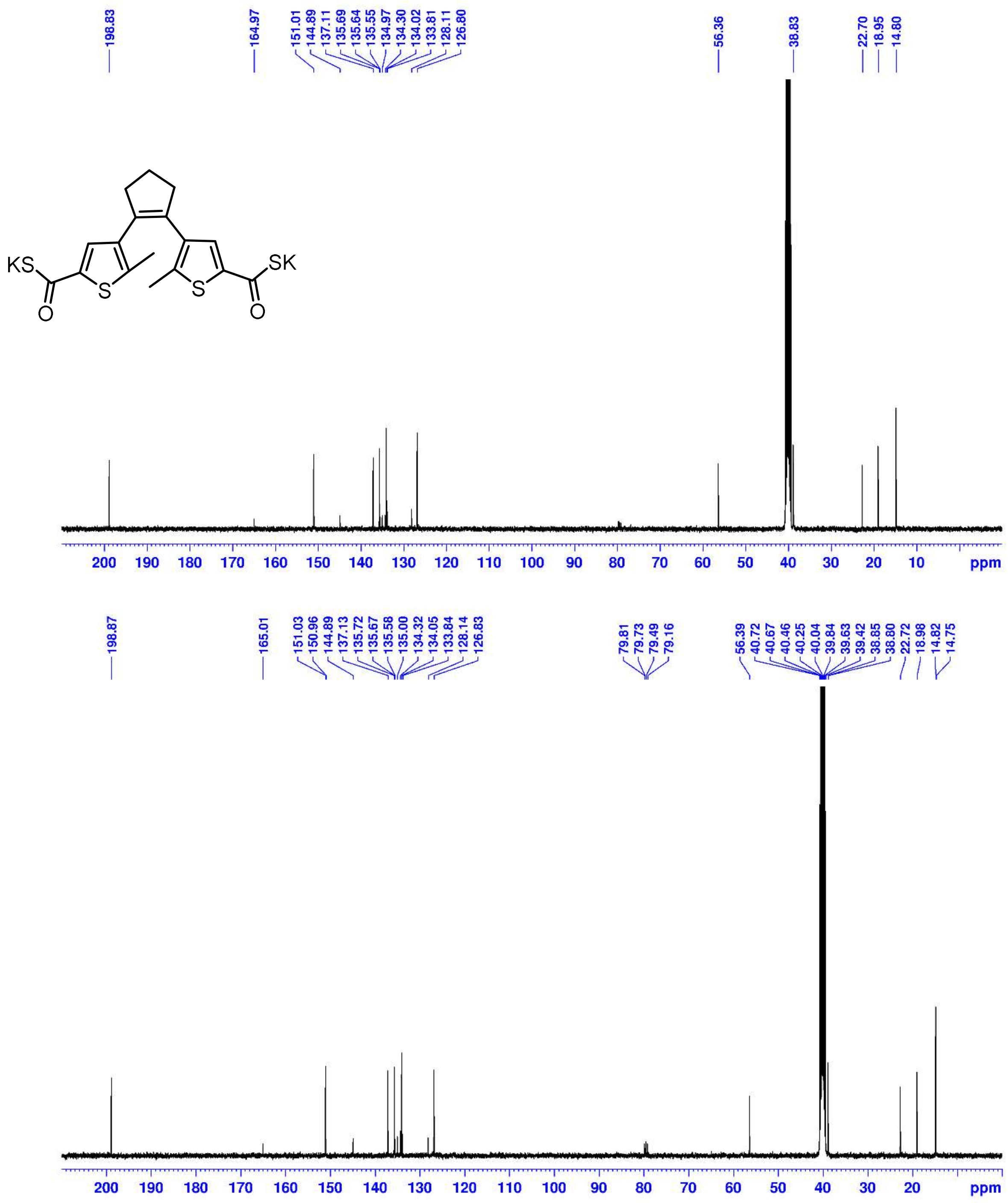

Figure S34. The ^{13}C NMR (D_6-DMSO, 100 MHz) of potassium salt of compound **5**, top is freshly prepared sample, the bottom spectrum is obtained after one week. The sample contains a small amount of ethanol.

VI. The UV-Vis spectra of compound **5** with different bases

1. Potassium salt of compound **5**

Compound **5** (6 mg, 0.016 mmol, 1 eq) was added to a scintillation vial and dissolved in EtOH (2 mL), KOH (1.8 mg, 0.032 mmol, 2 eq) was added and sonicated for 15 min, the solution turned deep purple. Then solvent was removed under reduced pressure to afford a purple solid as the potassium salt. This so prepared potassium salt (weighed 1.0 mg) was dissolved in 2 mL DI water to prepare a solution, the concentration was 1.09 mM. Then 0.2 mL of this solution was diluted to 2 mL aqueous solution to give a solution, the concentration was 0.11 mM. This solution was transferred to a cuvette and UV-Vis spectrum was taken after exposing the solution under 302 nm wavelength for different times.

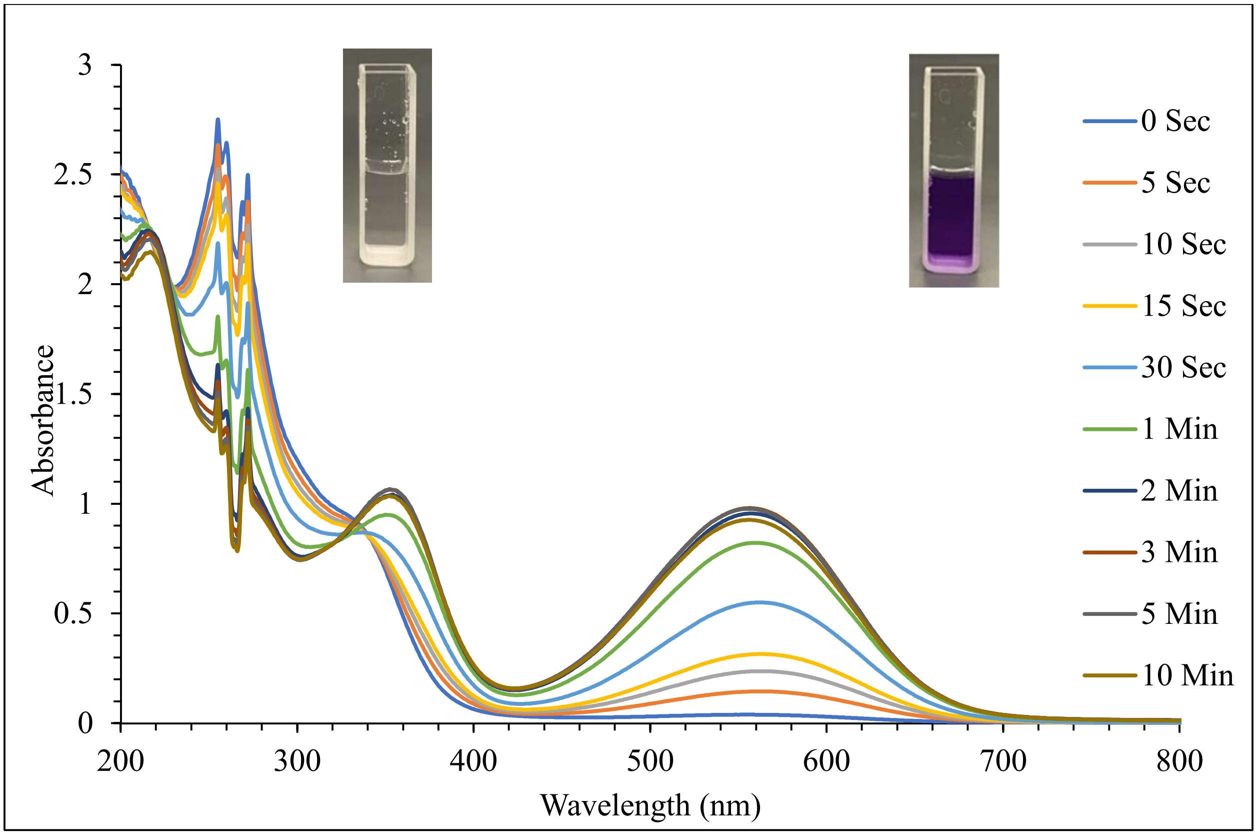

Figure S35. UV-Vis absorption spectra of potassium salt of compound **5**, 0.11 mM in water.

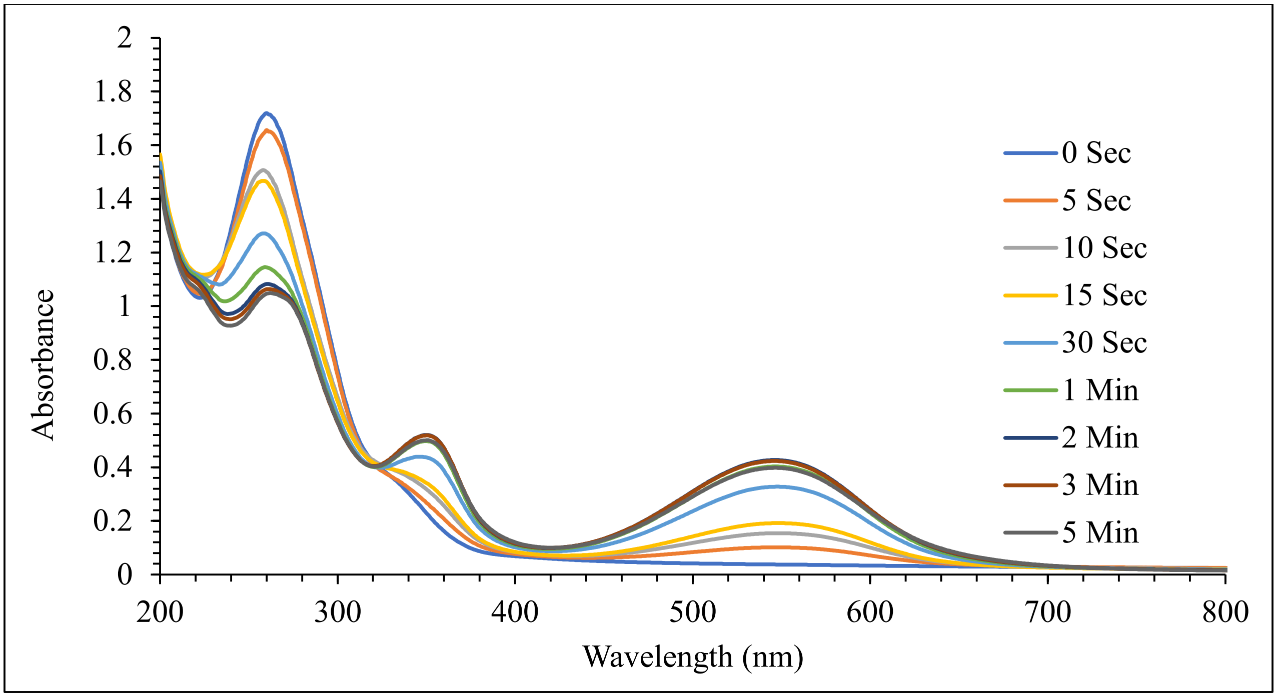

Figure S36. UV-Vis absorption spectra of potassium salt of compound **5**, 0.11 mM in acetonitrile.

2. UV-Vis spectra of the DIEA salt of compound **5**

Compound **5** (6 mg, 0.016 mmol, 1 eq) was added to a 1-dram vial, then EtOH and diisopropyl ethylamine (DIEA) (5 μL, 0.032 mmol, 2 eq) were added and sonicated for 15 min, the solution turned into light purple color. Then solvent was removed under reduced pressure to afford a light purple solid. 1.2 mg solid was dissolved in 2 mL DI water to prepare 1 mM solution. 0.2 mL of this solution was diluted to 2 mL solution to made 0.1 mM aqueous solution and UV-Vis was taken after exposing the solution under 302 nm wavelength for different times.

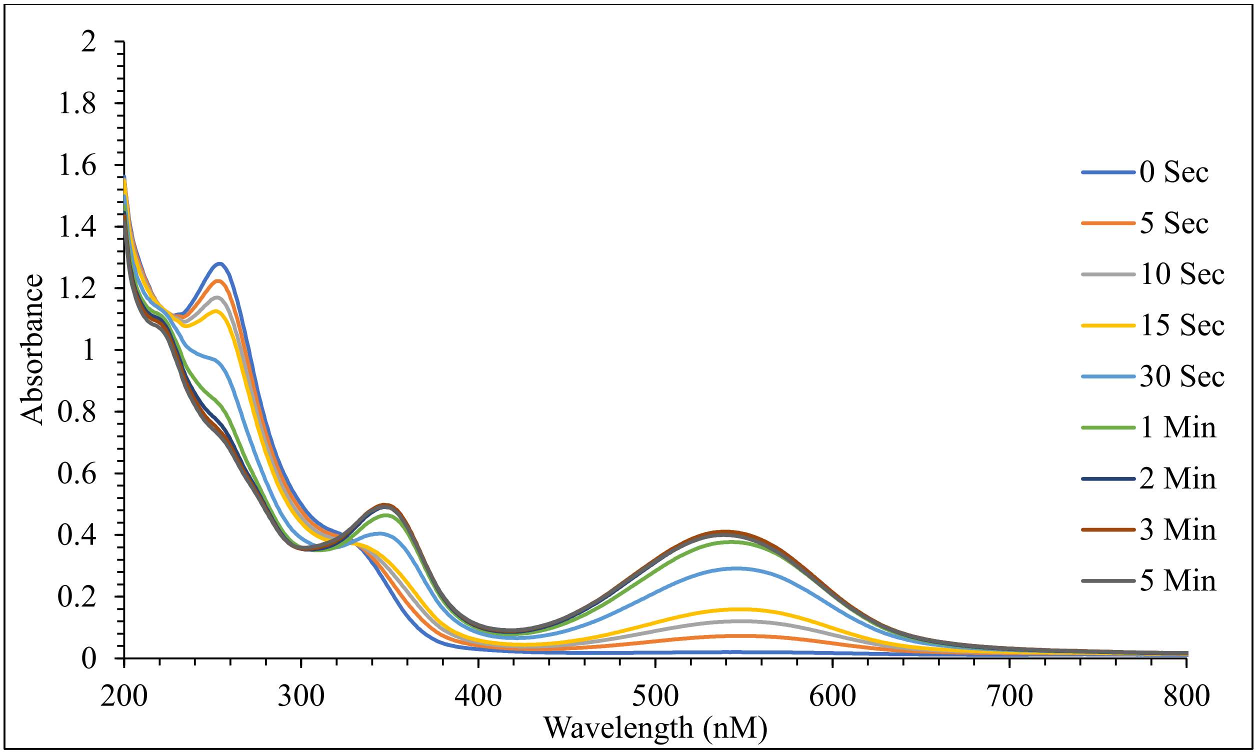

Figure S37. UV-Vis absorption spectra DIEA salt of compound **5**, 0.10 mM in DI water.

The UV-Vis spectra of the complex in acetonitrile were also obtained and are shown in Figure S38. To prepare the sample, 1.2 mg of the solid was dissolved in 2 mL acetonitrile to prepare 1 mM solution. Then 0.2 mL of this solution was diluted to 2 mL solution to made 0.1 mM acetonitrile solution and UV-Vis was taken after exposing the solution under 302 nm wavelength for different times.

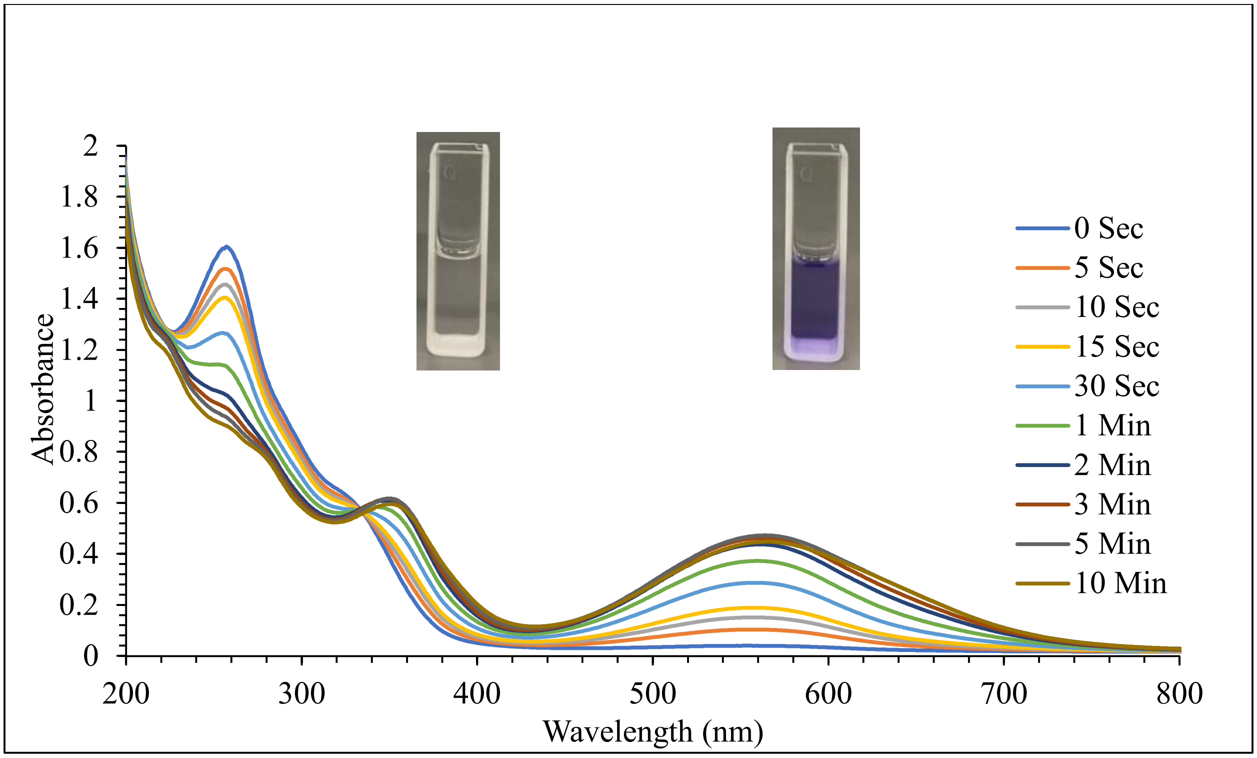

Figure S38. UV-Vis absorption spectra of DIEA salt of compound **5**, 0.10 mM in acetonitrile.

2. UV-Vis spectra of compound **5** with p-methoxy benzylamine

Compound **5** (6 mg, 0.016 mmol, 1 eq) was added to a 1-dram vial, then EtOH and 4-methoxy benzylamine (3.5 μL, 0.026 mmol, 2 eq) were added and sonicated for 15 min, the solution turned into purple color. Then solvent was removed under reduced pressure to afford purple solid. 1.3 mg solid was dissolved in 2 mL DI water to prepare 1 mM solution. 0.2 mL of this solution was diluted to 2 mL solution to made 0.1 mM aqueous solution and UV-Vis was taken after exposing the solution under 302 nm wavelength for different times.

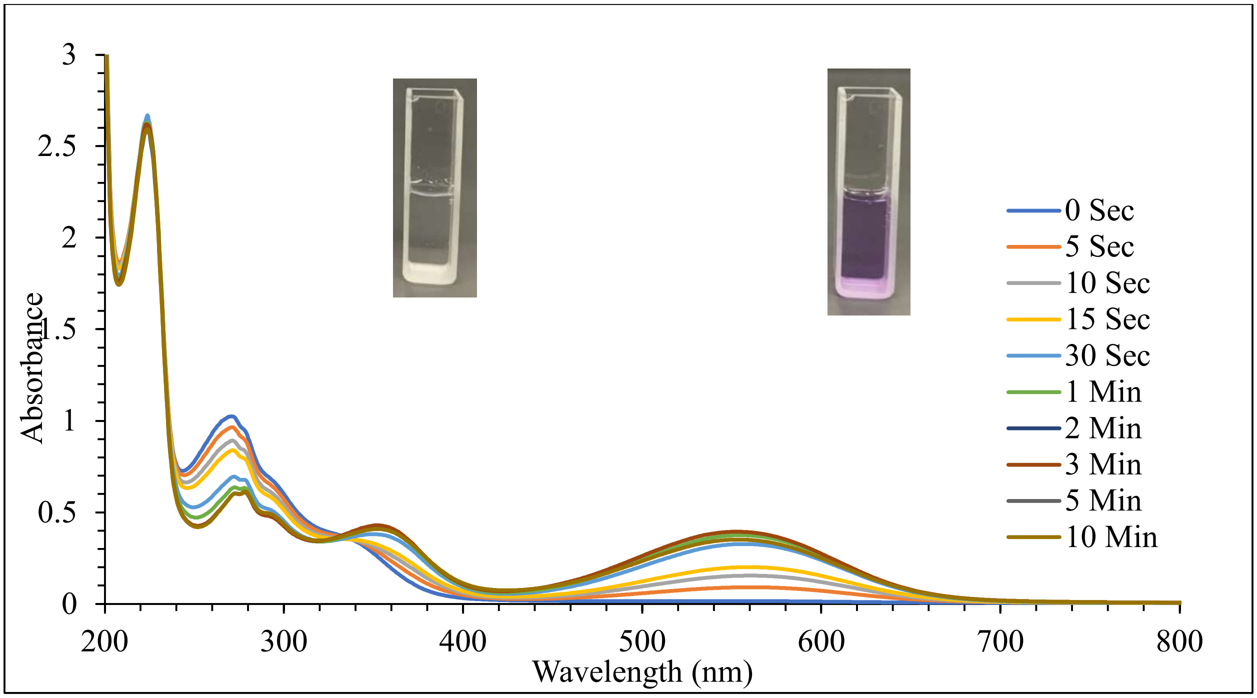

Figure S39. UV-Vis absorption spectra of *p*-methoxy benzylamine compound **5**, 0.10 mM in DI water.

The photoswitching was also studied in acetonitrile. 1.3 mg of the solid was dissolved in 2 mL acetonitrile to prepare 1 mM solution. 0.2 mL of this solution was diluted to 2 mL solution to made 0.1 mM acetonitrile solution and UV-Vis was taken after exposing the solution under 302 nm wavelength for different times.

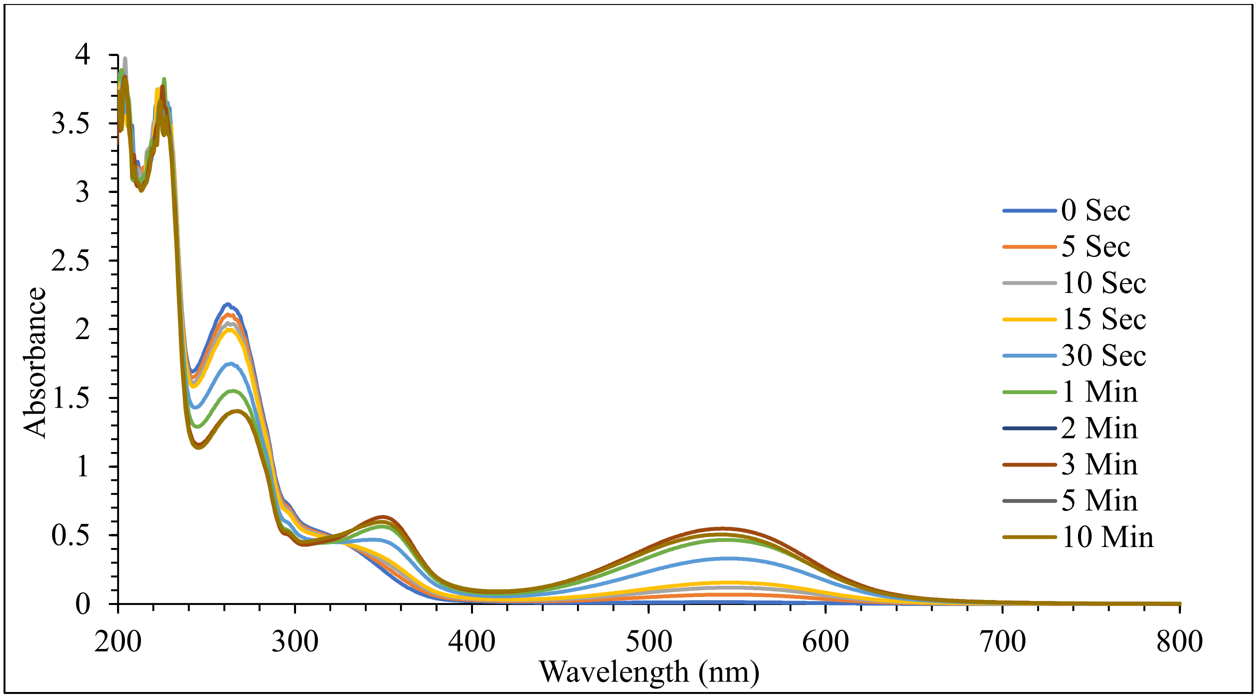

Figure S40. UV-Vis absorption spectra of p-methoxy benzylamine compound **5** 0.10 mM in acetonitrile.

VII. Comparisons of the photoswitching properties of compound **5** with compound **9b**

The photoswitching properties of compounds **9b** and compound **5** were compared and the spectra are included in Figures S41-S45, to estimate of the quantum yield (QY) of the open to the closed forms. Compound **9b** (2.7 mg) was dissolved in 4 mL acetonitrile to obtain a 1.54 mM solution, the solution was serial diluted to obtain two 2 mL acetonitrile solutions at 0.065- and 0.01-mM concentrations. The solutions were transferred to a quartz cuvette (10 mm, 3.5 mL with sealed cap), then the sample was treated with 302 nm UV light (6W), the spectra are included in Figures S41-S42.

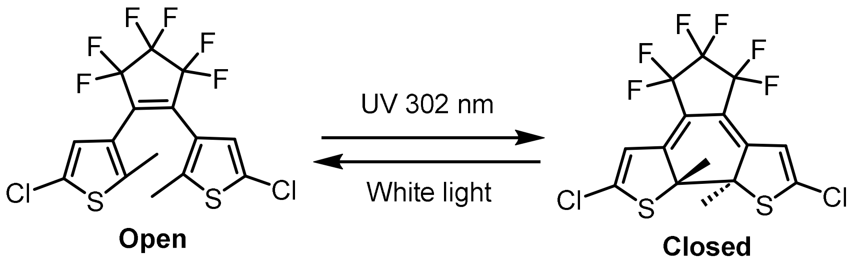

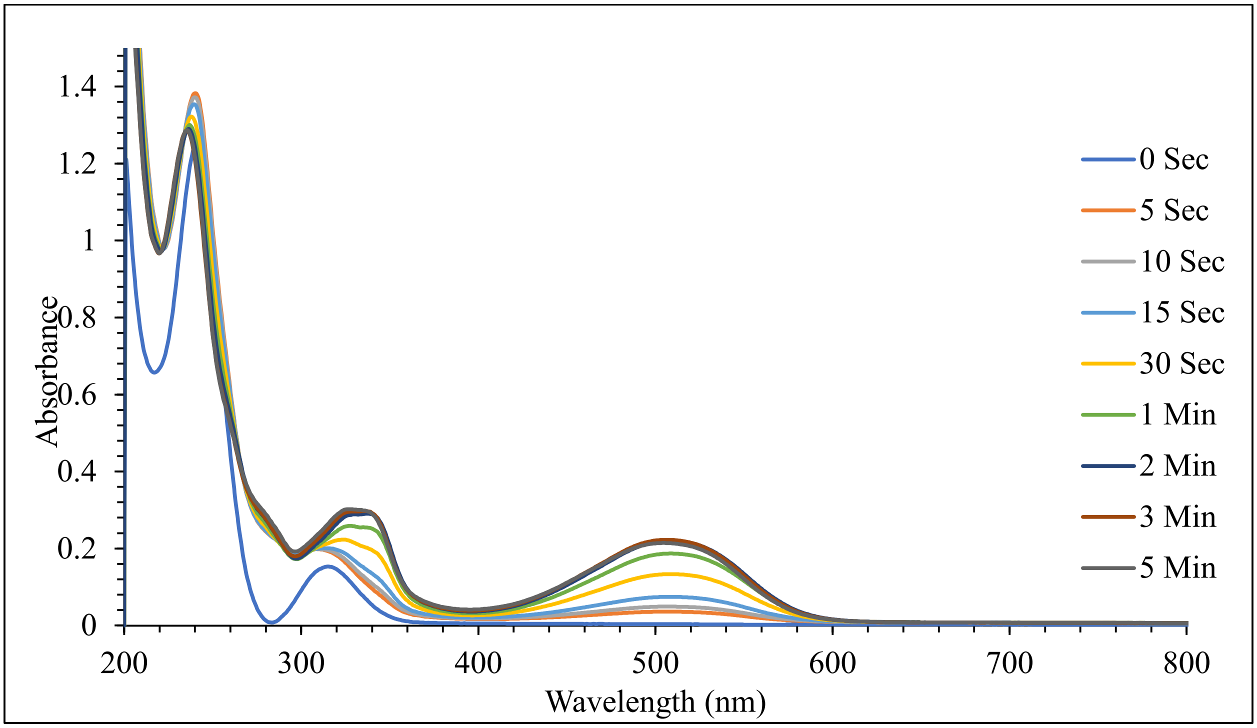

Figure S41. UV-Vis absorption spectra compound **9b** at 0.065 mM concentration in acetonitrile under 302 nm UV light irradiation at different time points.

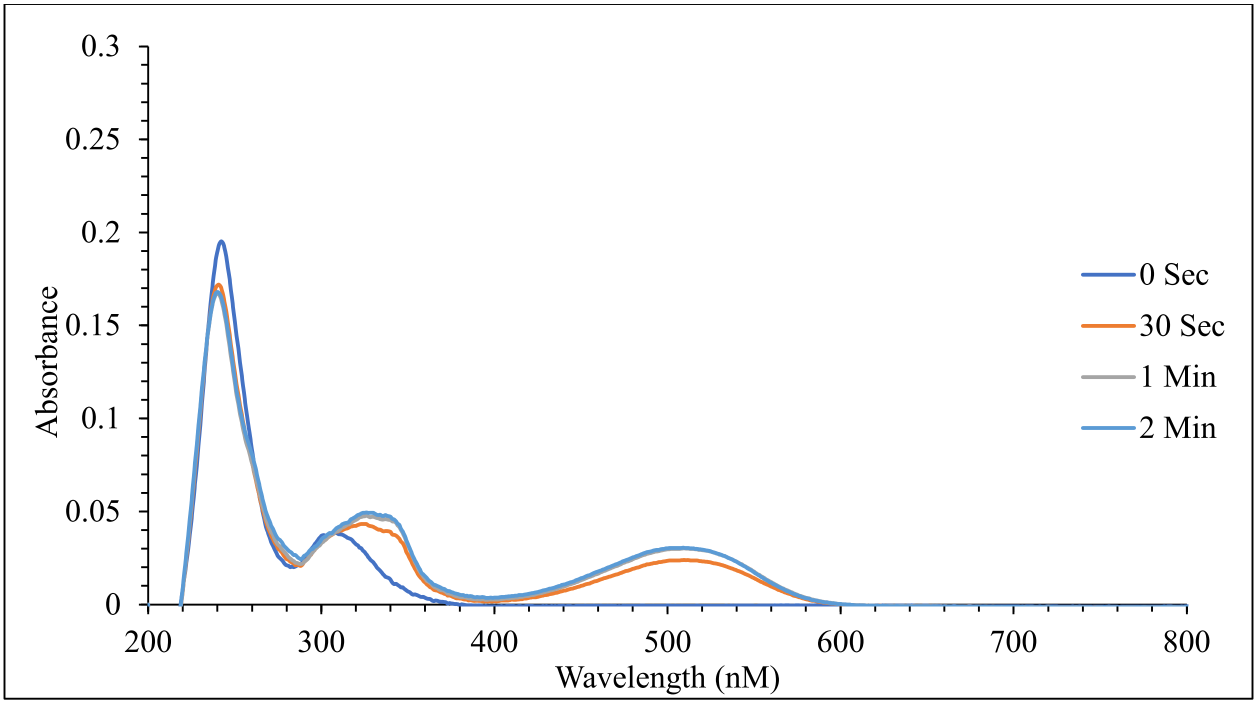

Figure S42. UV-Vis absorption spectra of compound **9b** at 0.01 mM concentration in acetonitrile under 302 nm UV light irradiation at different time points.

To compare the photocyclization reactions, UV-Vis absorption spectra of compound **5** at 0.01 mM in acetonitrile under 302 nm UV light were also acquired and the spectra are included in Figure S43. The closed form of compound **9b** reached maximum absorbance at 508 nm after 1 minute UV irradiation with 302 nm, the QY reported in the literature at 313 nm was 0.47. Compound **5** also reached maximum absorbance (λmax at ~600 nm) after 1 min irradiation with 302 nm light, the assumption is that both compounds have similar ratio of closed form versus the open form when they reach the photo stationary phase, therefore the QY for the compound **5** is at the similar range of compound **9b** at 0.47.

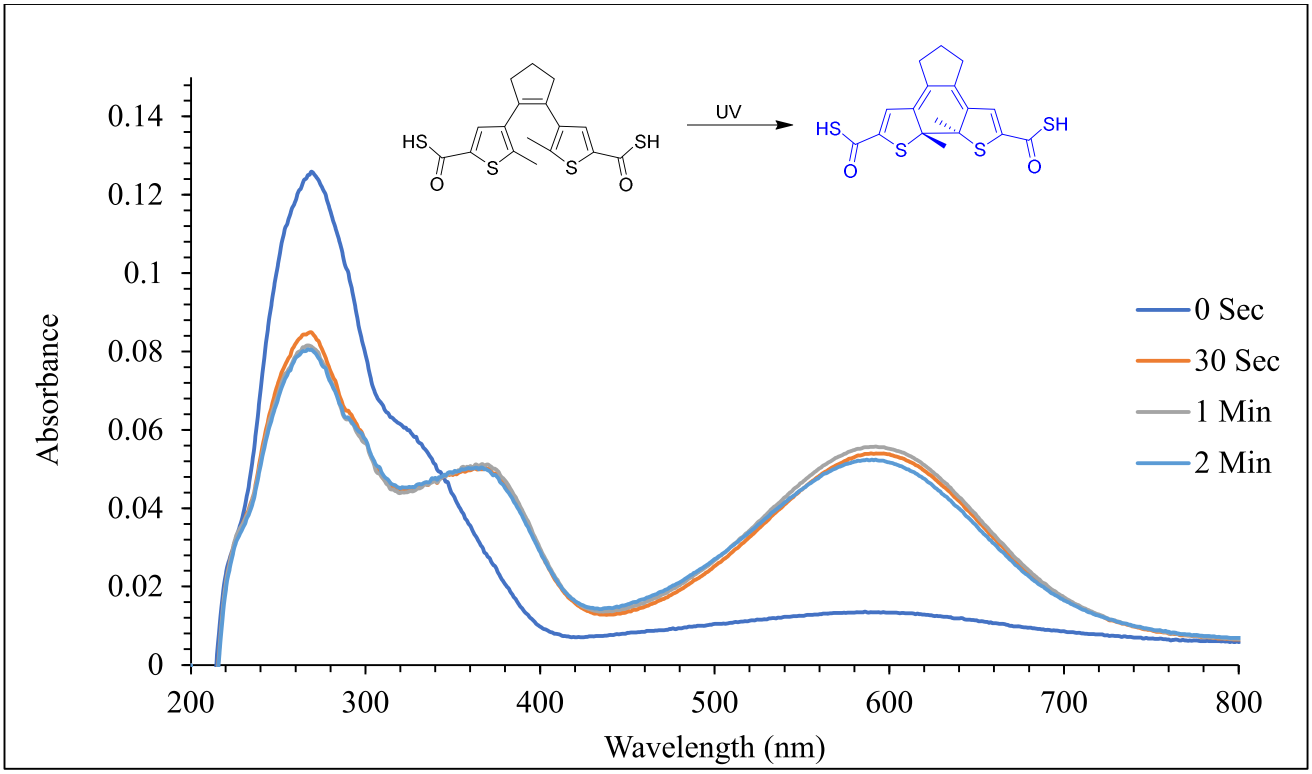

Figure S43. UV-Vis absorption spectra of compound **5** at 0.01 mM in acetonitrile. The solution was prepared by serial dilution of the 1.57 mM solution of compound **5** (1.2 mg in 2 mL acetonitrile), then 0.013 mL solution was diluted to 2 mL acetonitrile solution to make 0.01 mM solution.

The comparison of reversed photoconversion is shown in Figures S44 and S45. The 0.01 mM solution of compound **9b** or compound **5** was exposed under UV light 302 nm for 1 min to obtain the fully closed forms, then white light was passed through the cuvette and for 1 min, the UV-Vis spectra are shown in Figures S44 and S45. The two compounds exhibited similar extend of conversion at 1 minute irradiation, the compound **5** may have similar QY to compound **9b** for the closed to open form near 0.13.

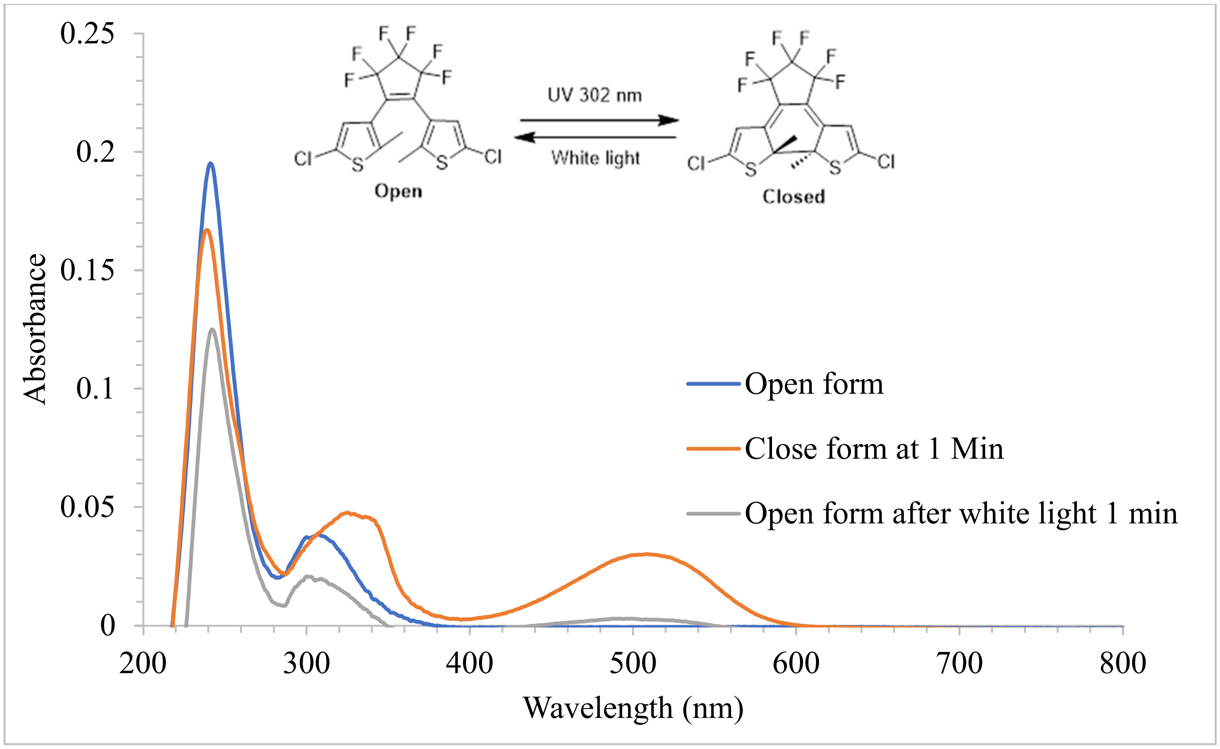

Figure S44. UV-Vis absorption spectra of compound **9b** at 0.01 mM in acetonitrile.

0.01 mM solution was exposed under UV light 302 nm for 1 min to obtain fully closed form then white light was passed to the cuvette for 1 min and absorbance was measured.

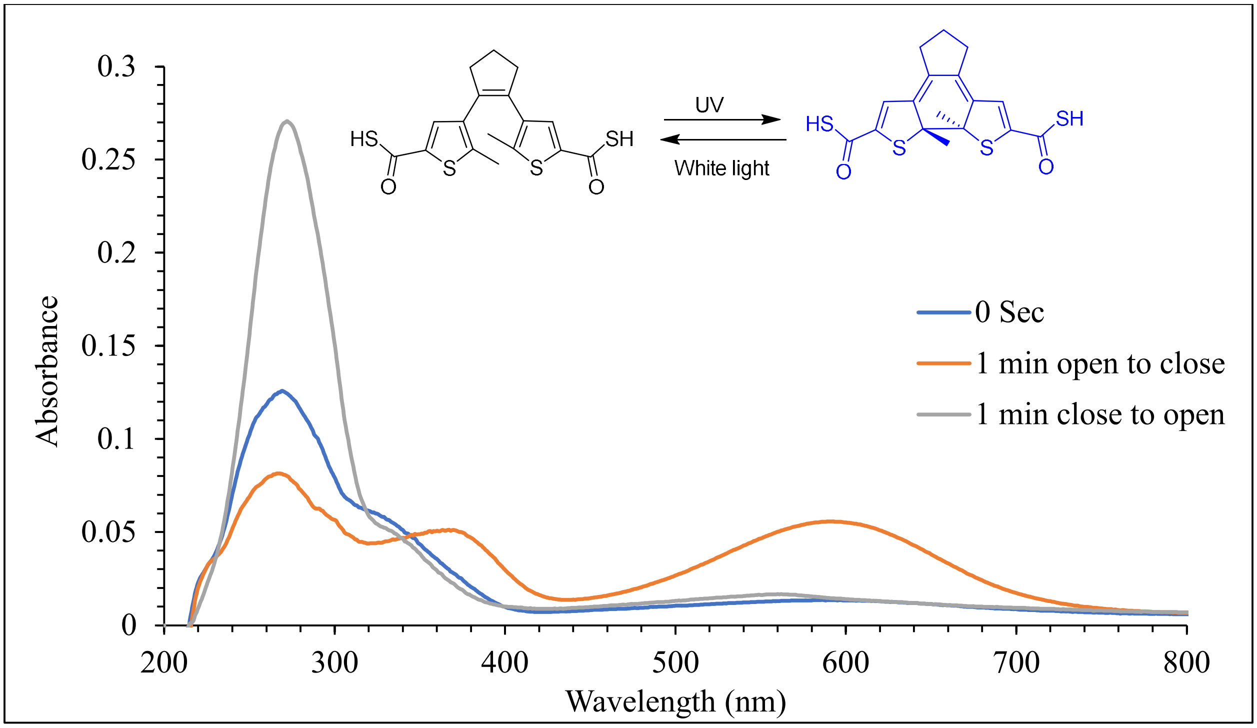

Figure S45. UV-Vis absorption spectra of compound **5** at 0.01 mM in acetonitrile. The 0.01 mM solution was exposed under UV light 302 nm for 1 min to obtain fully closed form then white light was passed to the cuvette for 1 min and absorbance was measured.

VIII. Photochromic hydrogels prepared using compounds **5-8**

1. Hydrogels prepared using compound **5**

Compound **5** (6 mg, 0.016 mmol), EtOH (2 mL) and KOH (1.8 mg, 0.032 mmol) were added and sonicated for 15 min in a scintillation vial, the solution turned deep purple color. Then solvent was removed under reduced pressure to afford a purple solid. A solution was prepared by dissolving the potassium salt of compound **5** (1. 0 mg) in 4.0 mL water to an aqueous solution (0.25 mg/mL). In a two-dram vial, agarose (20 mg), H_2O (4 mL), and 1.0 mL of the solution were added and the mixture was then heated and allowed to cool. Gel formation was evaluated by vial inversion method as shown to the right, the concentration of the potassium salt of compound **5** in the gel is 0.063 mg/mL. The agarose hydrogel was then reheated to form a solution, which was transferred to different shaped molds to form the gel arts. These are shown in Figure S46, the initial wedge and bees are transparent and color less, after irradiation with UV light, the gels turned deep purple color. The purple color gels turned colorless upon white light treatment.

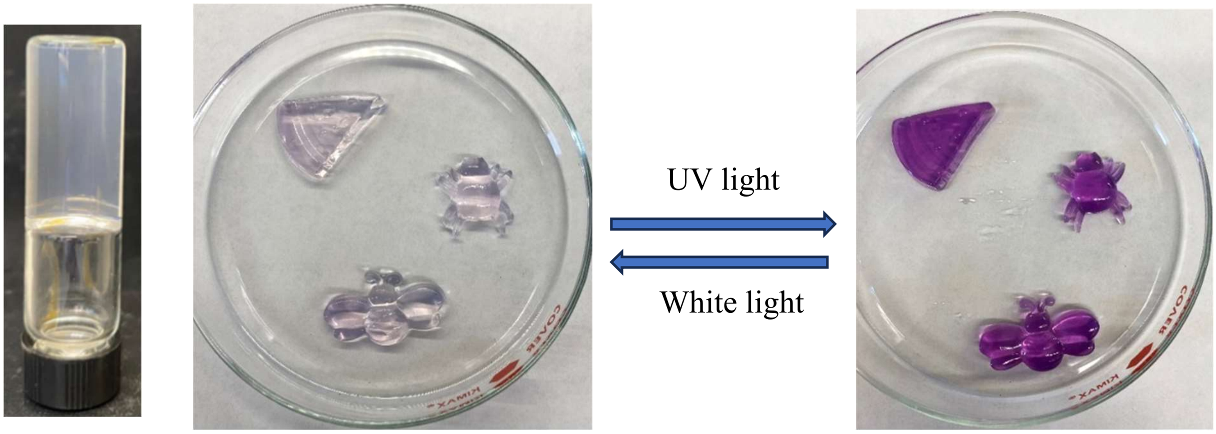

Figure S46. Hydrogels formed by compound **5** and agarose. The left figure shows the gels with the open form, and the right shows the gels after UV light (6W, 302 nm) exposure for 5 minutes.

2. Hydrogels prepared using compound **6**

Compound **6** (5 mg, 0.010 mmol, 1 eq) was taken in scintillation vial dissolved in EtOH and KOH (1.2 mg, 0.020 mmol, 2 eq) was added and sonicated for 15 min and then solvent was removed under reduced pressure to produce the potassium salt as solid. A solution was prepared by dissolving the potassium salt of compound **6** (1. 0 mg) in 4.0 mL water, the solution concentration is 0.25 mg/mL. In a two-dram vial, agarose (20 mg), H_2O (4 mL), and 1.0 mL of the above solution were added and mixed. The mixture was then heated and allowed to cool to form a gel, as shown on the right, the concentration of the potassium salt of compound **6** in the gel is 0.063 mg/mL. The agarose hydrogel was then reheated to form a solution, which was transferred to different shaped molds to form the gel arts. These are shown in Figure S47, the initial gel shapes (cookie, bee, and butterfly) are transparent and color less, after irradiation with UV light, the gels turned blue color. The blue color gels turned colorless upon white light treatment.

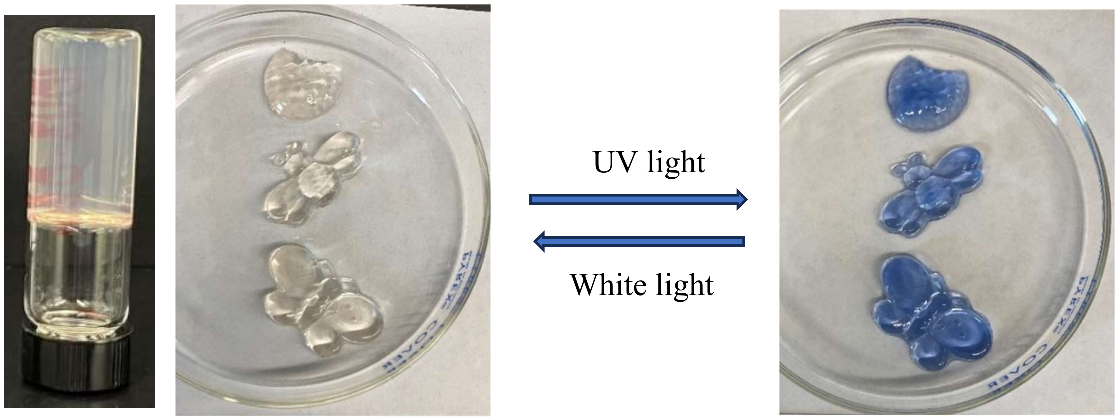

Figure S47. Hydrogels formed by compound **6** and agarose. The left figure shows the gels with the open form, and the right shows the gels after UV light (6W, 302 nm) exposure for 2 minutes.

3. Hydrogels prepared using compound **7**

Compound **7** (5 mg, 0.0094 mmol, 1 eq) was added to a scintillation vial, followed by EtOH (2 mL) and KOH (1.05 mg, 0.019 mmol, 2 eq), the mixture was sonicated for 15 min, and the solution was removed reduced pressure to afford a solid. A solution was prepared by dissolving the potassium salt of compound **7** (1. 0 mg) in 4.0 mL water, the solution concentration is 0.25 mg/mL. In a two-dram vial, agarose (20 mg), H_2O (4 mL), and 1.0 mL of the above solution were added and mixed. The mixture was heated and cooled to form the gel, as shown in the photo. The concentration of the potassium salt of compound **7** in the gel is 0.063 mg/mL. The agarose hydrogel was then reheated to form a solution, which was transferred to different shaped molds to form the gel shapes.

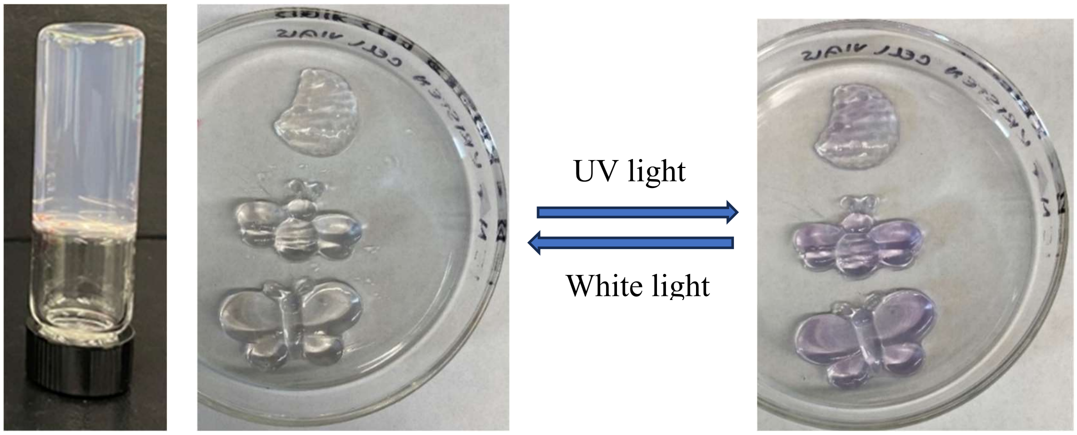

Figure S48. Hydrogels formed by compound **7** and agarose. The left figure shows the gels with the open form, and the right shows the gels after UV light (6W, 302 nm) exposure for 30 seconds.

4. Hydrogels prepared using compound **8**

Compound **8** (5 mg, 0.0078 mmol, 1 eq) was dissolved in EtOH (2 mL) in a scintillation vial, and KOH (0.8 mg, 0.0156 mmol, 2 eq) was added, the mixture was sonicated for 15 min, and the solution was removed reduced pressure to afford a solid. A solution was prepared by dissolving the potassium salt of compound **8** (1. 0 mg) in 4.0 mL water, the solution concentration was 0.25 mg/mL. In a two-dram vial, agarose (20 mg), H_2O (4 mL), and 1.0 mL of the above solution were added and mixed. The mixture was then heated and allowed to cool. Gel formation was evaluated by vial inversion method as shown to the right, the concentration of the potassium salt of compound **8** in the gel was 0.063 mg/mL. The agarose hydrogel was then reheated to form a solution, which was transferred to different shaped molds to form the gel arts and are shown in Figure S49.

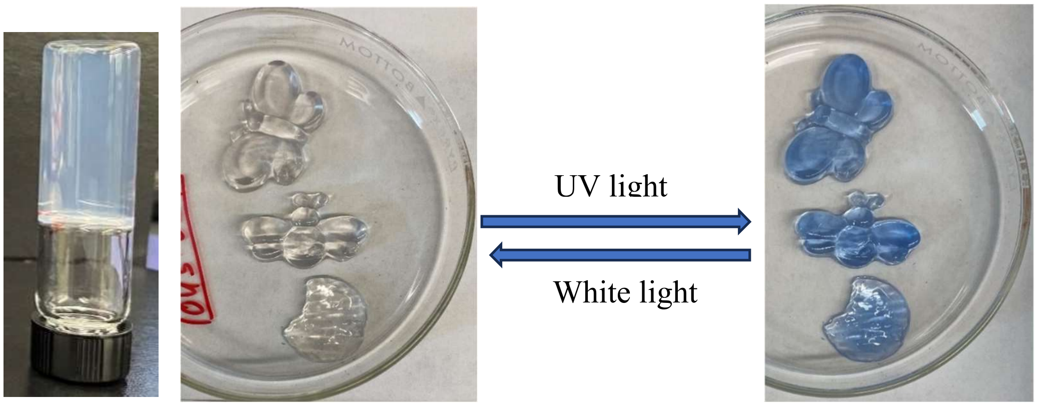

Figure S49. Hydrogels formed by compound **8** and agarose. The left figure shows the gels with the open form, and the right shows the gels after UV light (6W, 302 nm) exposure for 2 minutes.
